# A queen odour mediates reproductive suppression in a eusocial mammal

**DOI:** 10.1038/s41586-026-10772-5

**Published:** 2026-07-15

**Authors:** Mohammed A. Khallaf, Daniel W. Hart, Wenhan Luo, Firdevs Murad, Felipe Cybis Pereira, Daniel Mendez-Aranda, Nicole Hagenah, Alice Rossi, Valérie Bégay, Jan Okrouhlík, Dietmar Krautwurst, Mungo Kisinza Ngalameno, Andre Ganswindt, Alison J. Barker, Radim Šumbera, Markus Knaden, Sophie Pezet, Andrew Woehler, Bill S. Hansson, Nigel C. Bennett, Gary R. Lewin

**Affiliations:** 1 Molecular Physiology of Somatic Sensation Laboratory, Max Delbrück Center for Molecular Medicine in the Helmholtz Association (MDC), Berlin, Germany; 2 Department of Zoology and Entomology, Faculty of Science, Assiut University, Assiut, Egypt; 3 Department of Zoology and Entomology, University of Pretoria, Pretoria, Republic of South Africa; 4 Social Systems and Circuits Group, Max Planck Institute for Brain Research, Frankfurt am Main, Germany; 5 Charité-Universitätsmedizin Berlin, Berlin, Germany; 6 Physics for Medicine Paris, INSERM, ESPCI Paris, CNRS, PSL Research University, Paris, France; 7 Iconeus, Paris, France; 8 Department of Evolution of Sensory and Physiological Systems, Max Planck Institute for Biological Intelligence, Martinsried, Germany; 9 Department of Zoology, Faculty of Science, University of South Bohemia, České Budějovice, Czech Republic; 10 Leibniz Institute for Food Systems Biology at the Technical University of Munich, Freising, Germany; 11 Department of Veterinary Anatomy and Pathology, Sokoine University of Agriculture, Morogoro, Tanzania; 12 Department of Evolutionary Neuroethology, Max Planck Institute for Chemical Ecology, Jena, Germany; 13 Howard Hughes Medical Institute, Janelia Research Campus, Ashburn, VA USA; 14 Mammal Research Institute, Department of Zoology and Entomology, University of Pretoria, Pretoria, Republic of South Africa; 15 German Center for Mental Health (DZPG), partner site Berlin, Berlin, Germany

**Keywords:** Molecular ecology, Social evolution, Olfactory receptors

## Abstract

Eusociality is exceptionally rare in mammals, and its proximate mechanisms remain poorly understood. Naked mole-rats provide a striking example, with reproduction restricted to a single queen, while all other females remain infertile^1^. Here we identify a chemical signal in queens that can regulate this reproductive hierarchy. Isopropyl myristate is a low-volatility ester that is enriched in queens and nearly absent from non-breeding animals. Isopropyl myristate is detected by peripheral and central olfactory neurons and elicits avoidance in high-ranking animals. Exposure alters levels of prolactin and progesterone in non-breeders to suppress reproduction. Daily addition of isopropyl myristate to a queenless colony prevented queen succession, whereas withdrawal triggered aggression and reproductive competition. Isopropyl myristate was also detected in breeding females of several *Fukomys* mole-rat species, but reaches higher levels in naked mole-rats, paralleling their extreme reproductive skew. These findings reveal a chemical cue that can mediate reproductive suppression in a mammal, linking insect and mammalian eusociality.

## Abstract (web summary)

Naked mole-rat (*Heterocephalus glaber*) queens produce isopropyl myristate, a low-volatility ester that high-ranking animals detect and avoid, which alters prolactin and progesterone in other females to suppress reproduction and preserve eusocial hierarchy.

## Main

Naked mole-rats (*Heterocephalus glaber*) are remarkable for their eusocial lifestyle, a rare social structure that is more commonly found in invertebrates such as bees, termites and ants^1,2^. Eusociality is characterized by cooperative care of offspring, overlapping generations and extreme reproductive skew, with reproduction typically monopolized by a dominant breeding female, the queen, who is normally the sole reproducing female in the colony^3–5^. Indeed, naked mole-rats are at the extreme end of a mammalian eusociality spectrum^2^. An essential feature of this complex social organization is the suppression of reproduction among colony members, a process that is primarily regulated by the queen^6^. Despite decades of research following the discovery of eusociality in mammals^1^, the precise mechanism by which queens govern colony structure and suppress reproduction has remained unknown.

Reproductive suppression through chemical communication, particularly via olfactory cues, is a common mechanism by which dominant individuals influence the reproductive physiology of conspecifics^7^. In mammals, olfactory cues can trigger profound reproductive consequences, including pregnancy disruption (the Bruce effect)^8^, suppression of ovulation in subordinates in conjunction with other social cues^9^, and the concealment of oestrus in social carnivores^10^. In eusocial insects, queen pheromones maintain colony harmony by inhibiting worker reproduction^11,12^, suggesting an evolutionary precedent for chemically mediated reproductive control. In naked mole-rats, reproductive suppression depends on the presence of the queen^1^ and is rapidly reversed upon her death or removal^13^. Colonies possess distinct odour signatures that facilitate recognition of nestmates versus outsiders^14,15^, and queen scent correlates with changes in cooperative task performance, suggesting a ‘social organizer’ role^16^. These observations indicate that olfactory communication is central to social cohesion, cooperative behaviour and probably to reproductive suppression, yet the identity and function of a chemical signal produced by queens remained unknown.

Here we combine behavioural, chemical and neuroendocrine analyses to identify and functionally characterize a queen-derived chemical signal in naked mole-rats. We show that olfactory cues underlie social recognition and modulate aggression, and that the long-chain ester isopropyl myristate (IPM) is uniquely present in queens and peaks in vaginal secretions during reproductive readiness. This compound is detected by olfaction, alters prolactin and progesterone levels in non-breeding females, and can prevent reproduction and maintain social stability even after queen removal. These findings reveal a queen-enriched chemical that contributes to reproductive suppression and social cohesion, and uncover a fundamental mechanism that underlies mammalian eusocial systems.

## Odour-based recognition in naked mole-rats

**Fig. 1:**
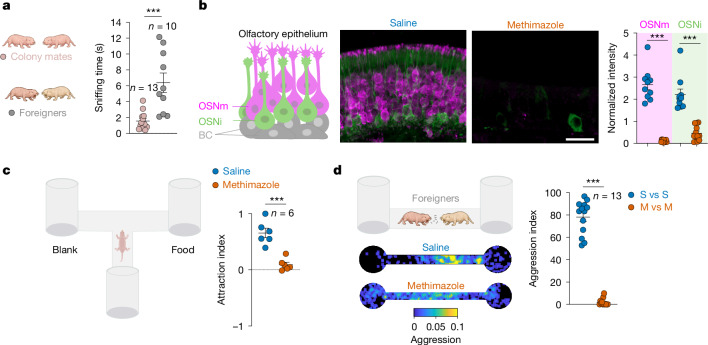
Naked mole-rats rely on olfaction to locate food and recognize strangers. **a**, Time spent sniffing was significantly higher when animals encountered non-colony members (foreigner conspecifics, *n* = 10) compared with familiar colony mates (*n* = 13). Each dot represents one animal-pair interaction. Data are mean ± s.e.m.; two-sided unpaired *t*-test, ***P = 0.0001. **b**, Schematic (left) and immunofluorescent images (middle) of the olfactory epithelium showing mature OSNs (OSNm; OMP^+^, magenta) and immature OSNs (OSNi; STMN1^+^, green) 72 h after injection. Note that methimazole-injected animals showed near-complete ablation of mature and immature OSNs. Right, quantification of mean normalized fluorescence intensity indicated reductions in mature OSN and immature OSN markers (nested *t*-test, sections nested within animal; mature OSN: *t* = 8.96, d.f. = 4, ***P = 0.0009, and immature OSN: *t* = 12.76, d.f. = 4, ***P = 0.0002). Scale bar, 40 µm. Each dot represents one section (*n* = 10 sections from 3 saline-injected and 12 sections from 3 methimazole-injected animals). Data are mean ± s.e.m. BC, basal cell. **c**, Saline-injected animals displayed attraction to food odour compared with methimazole-injected animals in a two-choice assay. Attraction index = (time in food odour chamber − time in control chamber)/total time. Each dot represents one animal. *n* = 6 per group; data are mean ± s.e.m.; two-sided unpaired *t*-test, ***P = 0.0002. **d**, Left, heat maps show spatial distribution of aggressive interactions along the arena (averaged across pairs; each map normalized to sum to 1, colour = fraction of total aggression per bin, 0–0.1) between pairs of unfamiliar, high-ranking animals from different colonies, with both individuals in each pair receiving the same treatment: saline–saline (S vs S) or methimazole–methimazole (M vs M). Methimazole-injected animals exhibited reduced aggression compared with controls. Right, aggression index (proportion of time spent in aggressive behaviours (head-to-head shoving, biting, chasing and dominance displays) relative to total observation time). *n* = 13 per group; data are mean ± s.e.m.; two-sided Mann–Whitney test *P* < 0.0001. Source data

**Extended Data Fig. 1:**
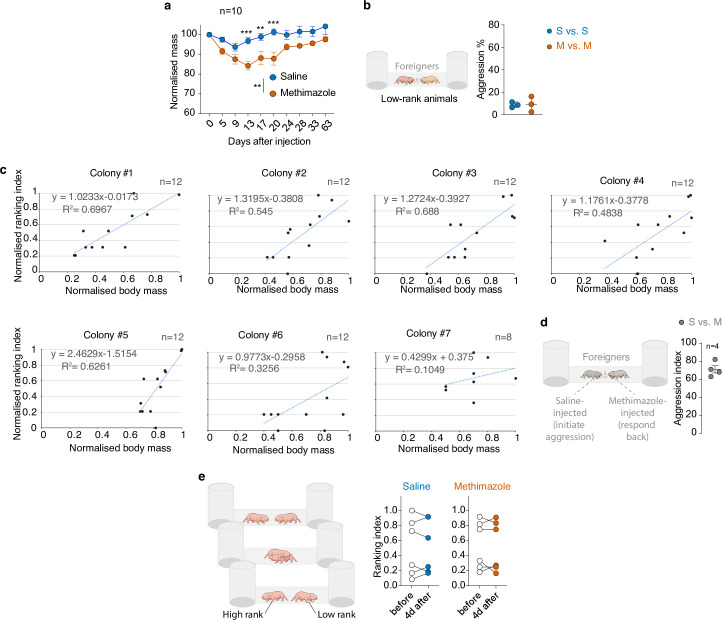
Loss of olfaction induces transient body mass loss without affecting social rank in naked mole-rats. **a**, Normalized body mass declined significantly in methimazole-injected animals compared to saline controls between days 5–20 post-injection. Data are presented as mean ± s.e.m. (n = 10 per group); two-way repeated measures ANOVA with Šídák’s multiple comparisons test, ***p = 0.0005 (day 13), **p = 0.0044 (day 17), ***p = 0.0002 (day 20). **b**, Low-rank naked mole-rats show low aggression regardless of treatment. Each dot represents one animal-pair interaction (n = 3, mean ± s.e.m.). Two-sided unpaired *t*-test, ns *p* > 0.05. **c**, Body mass correlates with social rank in some colonies. Linear regressions show positive relationships between body mass and social rank across seven colonies. The number of animals is indicated in each panel. **d**, Methimazole does not suppress aggressive behaviour. Schematic showing saline-injected animals initiate aggression, while methimazole-injected animals respond by fighting back. Aggression index quantification (right; n = 4, mean ± s.e.m.) shows preserved aggression in methimazole-treated animals (see Supplementary Video 6). **e**, Ranking indices remained stable before and after saline and methimazole injections. Each line represents an individual (n = 6). Two-sided paired *t*-test, ns *p* > 0.05. Source data

To examine the role of olfactory cues in social and foraging behaviours of naked mole-rats^14,15,17^, we first tested their ability to discriminate between familiar and unfamiliar individuals (Fig. 1a). We paired animals in a single tube and recorded their interactions. The animals spent more time sniffing non-colony individuals than colony mates, indicating recognition of social novelty (Fig. 1a and Supplementary Videos 1 and 2). To determine whether this behaviour is solely dependent on olfaction, we ablated olfactory sensory neurons (OSNs) using methimazole. In mice and rats, a single intraperitoneal injection induces rapid damage to the olfactory epithelium, affecting various cell types, including OSNs^18^. This damage begins within hours and involves cellular swelling and detachment from the basal lamina, leading to the near-complete loss of OSNs within 48 h (ref. ^18^). Our immunohistochemical analysis confirmed a near-complete loss of mature OSNs (immunopositive for OMP^19^) and immature OSNs (immunopositive for STMN1^20^) within 72 h of injection in naked mole-rats (Fig. 1b). Moreover, methimazole-treated animals showed no attraction to food odour and experienced a transient loss in body mass compared with saline-treated controls, which resolved within three weeks (Fig. 1c, Extended Data Fig. 1a and Supplementary Video 3).

In naked mole-rat colonies, both body size and social rank shape social roles: smaller, low-ranking individuals primarily carry out maintenance and foraging tasks, whereas some larger, high-ranking members defend the colony against intruders with overt aggression, including head-to-head shoving and biting^1,3,21^. We paired two unfamiliar, high-ranking animals from different colonies in a neutral arena, with both individuals receiving the same treatment (either saline or methimazole). In saline–saline pairings, high-ranking animals displayed robust aggressive interactions, including head-to-head shoving, biting and chasing. By contrast, aggression was markedly reduced in methimazole–methimazole pairings (Fig. 1d and Supplementary Videos 4 and 5). Low-ranking animals showed little aggression regardless of treatment (Extended Data Fig. 1b,c). Notably, in mixed-treatment encounters, methimazole-treated high-ranking animals actively fought back when challenged by saline-treated opponents, demonstrating that olfactory ablation selectively disrupts the initiation of aggression, rather than aggressive behaviour per se (Extended Data Fig. 1d and Supplementary Video 6). Notably, social rank remained stable in both saline- and methimazole-treated groups (Extended Data Fig. 1c,e), indicating that it is linked to body mass and maintained by other modalities, such as vocal communication^22^. These results demonstrate that naked mole-rats rely on olfaction to locate food and discriminate between colony mates and strangers.

## An odour enriched in queens

**Fig. 2:**
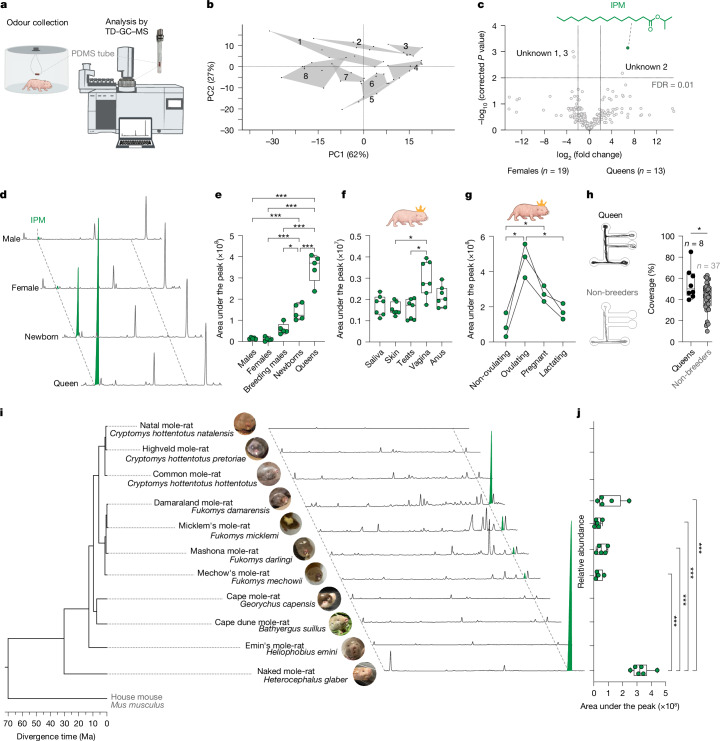
IPM is a queen-specific compound. **a**, Schematic showing odour collection and analysis. PDMS, polydimethylsiloxane. **b**, PCA of odour profiles. **c**, Bottom, volcano plot comparing odour profiles. log_2_ fold change (queen/female) was plotted against −log_10_ of the false discovery rate (FDR)-corrected *P* value, two-sided Welch *t*-test per compound with Benjamini–Hochberg FDR correction. Horizontal line represents the significance threshold (FDR = 0.01). Top, structure of IPM. **d**, Odour chromatograms from naked mole-rat colonies. IPM is highlighted in green. **e**, Quantification of IPM levels (*n* = 5 per group) showed higher abundance in queens (one-way analysis of variance (ANOVA) with Tukey’s post hoc test, *P = 0.02, ***P < 0.001). **f**, Quantification of IPM (*n* = 7 queens) showed higher levels in vaginal, anal and salivary secretions compared with skin and teats. Friedman test with Dunn’s multiple comparisons, *P = 0.0235 (skin versus vagina) and *P = 0.0406 (teats versus vagina). **g**, IPM levels (*n* = 3 queens) were significantly higher during ovulation compared with other reproductive states. Repeated-measures ANOVA with Greenhouse–Geisser corrections and Tukey’s multiple comparisons test, *P = 0.0425 (non-ovulating versus ovulating), *P = 0.0148 (non-ovulating versus pregnant), *P = 0.0271 (ovulating versus lactating). **h**, Movement traces showed that queens (*n* = 8) explored a novel maze more than non-breeders (*n* = 37). Two-sided unpaired *t*-test, *P = 0.0203. **i**, A time-calibrated phylogenetic tree of African mole-rats and house mouse (*Mus musculus*) with GC–MS chromatograms aligned to highlight IPM (green). Photo credits: the authors; and A. Janse van Vuuren for the Highveld mole-rat. **j**, IPM abundance across species (*n* = 5–6 per species). Naked mole-rat levels were higher than all other species (one-way ANOVA with Tukey’s multiple comparisons test, ***P < 0.001). Damaraland mole-rat levels did not significantly differ from other *Fukomys* species. *Cryptomys* and solitary species showed no detectable peak. In box plots in Fig. 2e,f,h,j, the centre line shows the median, box bounds delineate 25th–75th percentiles and whiskers extend from minimum to maximum values. Source data

**Extended Data Fig. 2:**
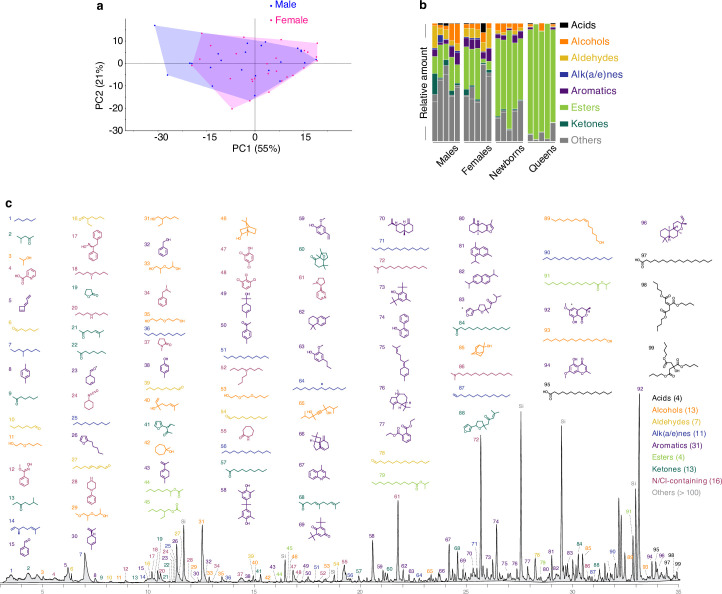
Chemical composition of naked mole-rat odour profiles. **a**, PCA of chemical profiles from non-breeding males (blue, n = 23) and females (pink, n = 27) naked mole-rats shows extensive overlap between the groups, indicating no clear sex-specific variation in odour composition. **b**, Ester peak areas dominate the queen odour profile, while other groups show more varied compositions of different chemical classes. Colour coding indicates compound class, n = 5 in each group. **c**, Representative GC-MS chromatogram displaying major volatile compounds collected from naked mole-rats. Each numbered peak corresponds to a chemically identified compound (structures shown above), categorized by functional group. Colour coding indicates compound class. Over 100 compounds were detected. Isopropyl myristate corresponds to peak #91. Source data

To investigate the chemical basis of olfactory discrimination we collected volatile compounds from 52 non-breeding individuals from 8 distinct colonies and analysed them using thermal desorption gas chromatography–mass spectrometry (TD-GC–MS)^23^ (Fig. 2a). Chemical analyses identified 232 compounds with distinct *m*/*z* (mass-to-charge ratios) across different replicates. Principal component analysis (PCA) revealed distinct clustering by colony, indicating colony-specific chemical signatures (Fig. 2b). However, no significant differences were observed between male and female non-breeding individuals (Extended Data Fig. 2a).

**Extended Data Fig. 3:**
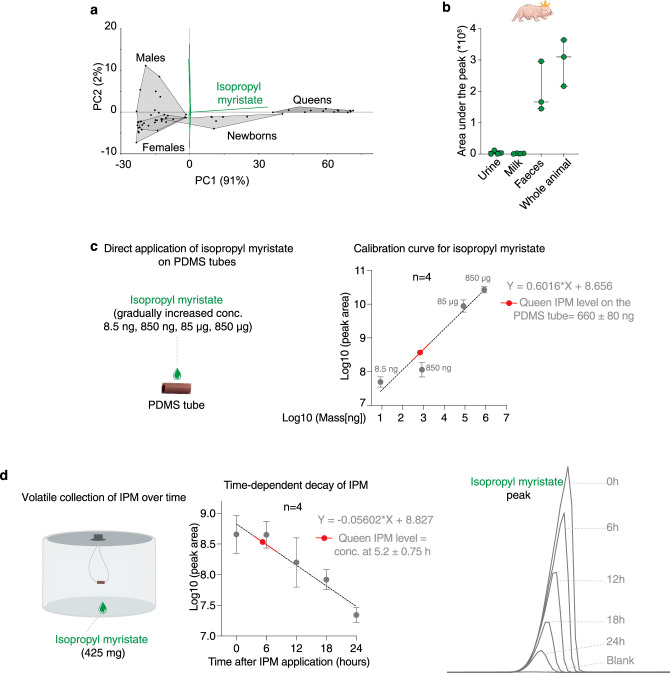
Quantitative analysis of isopropyl myristate levels. **a**, Queens form a distinct cluster in the PCA plot, with isopropyl myristate loading strongly along PC1, highlighting its contribution to queen-specific chemical signatures. **b**, Isopropyl myristate levels were high in whole-animal and faecal samples from queens, whereas urine and milk samples show little to no detectable signal (n = 3–4). Data are presented as median with min-to-max range. **c**, Left, schematic of PDMS tubes spiked with graded isopropyl myristate doses (8.5 ng–850 µg). Right, calibration curve of log_10_ peak area vs. log_10_ isopropyl myristate mass with linear fit (Y = 0.6016 × X + 8.656); red point and line indicate queen isopropyl myristate level (660 ± 80 ng, mean ± s.d., n = 4-7). **d**, PDMS tubes sampled isopropyl myristate (500 µL equal to 425 mg) from a chamber over 24 h. Peak area declined with time (mean ± s.d., n = 4), with queen isopropyl myristate levels corresponding to ~5.2 ± 0.75 h after application; representative GC–MS traces show progressive loss of the isopropyl myristate peak. Source data

Queens may produce distinctive odours that aid in social recognition and coordination^16^. To assess chemical differences between queens and non-breeding colony mates, we analysed volatile profiles from 771 samples representing 351 naked mole-rats across ranks, ages and reproductive states, detecting 240 compounds, 99 of which were chemically identified (Extended Data Fig. 2b,c and Supplementary Table 1). A volcano plot comparing queens with non-breeding females identified IPM as a compound that is significantly enriched in queens (Fig. 2c). This molecule was consistently abundant in queens but nearly undetectable in breeding and non-breeding males and non-breeding females. It was also present at low levels in newborns, probably owing to their repeated physical contacts with the queen (Fig. 2d,e and Extended Data Fig. 3a).

To determine the source of IPM, we analysed smears from various body sites and found it to be most abundant in vaginal, anal and mouth secretions (Fig. 2f). IPM levels were high in whole-animal and faecal samples from queens, but were almost absent from milk and urine (Extended Data Fig. 3b). We next examined how IPM levels varied across reproductive states. Levels peaked during ovulation and declined during pregnancy and lactation (Fig. 2g), linking this compound to reproductive readiness. Calibration experiments indicated that levels collected from queens corresponded to around 600 ng (Extended Data Fig. 3c,d).

IPM is a long-chain ester whose low volatility would be suitable for long-lasting chemical signalling. Consistent with previous observations that the queen is highly active in patrolling her colony^3,24^, we quantified exploratory behaviour of queens and non-breeders in a novel maze using video-based tracking. Queens explored a significantly larger proportion of the maze than non-breeders (Fig. 2h), in line with recent quantitative findings that breeders more actively patrol the colony space compared with non-breeders^25^. Together, our findings demonstrate that IPM is enriched in queens and is a candidate chemical signal linked to reproductive status in naked mole-rats.

Naked mole-rats represent the clearest example of eusociality in mammals. Consistently, IPM abundance was highest in naked mole-rats, but it was also detected in reproductive females from all four *Fukomys* species tested, and was absent from *Cryptomys* and the solitary species *Georychus capensis*, *Bathyergus suillus* and *Heliophobius emini*) (Fig. 2i,j). These findings indicate that IPM production is not a general feature of mole-rat sociality, but may be enriched in social species exhibiting high reproductive skew.

## Olfactory detection of IPM

**Extended Data Fig. 4:**
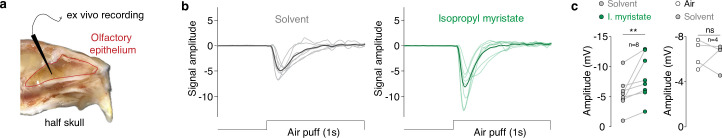
Isopropyl myristate evokes olfactory sensory responses in naked mole-rats. **a**, Photograph of a naked mole-rat half-skull preparation used for ex vivo electro-olfactogram (EOG) recordings, with the olfactory epithelium exposed. **b**, Representative EOG traces in response to a 1-second air puff of solvent (DMSO) and isopropyl myristate diluted 1:10 (v/v) in DMSO. **c**, Quantification of EOG peak amplitude. Left, solvent vs. isopropyl myristate (n = 8); right, air vs. solvent (n = 4). Two-sided paired t-test; ns *p* > 0.05, **p = 0.008. Source data

As in humans, naked mole-rats have a vestigial vomeronasal organ, a chemosensory organ that is typically implicated in pheromone detection^26,27^. Notably, naked mole-rats have an expanded repertoire of odorant receptors compared with other rodents^28^. To determine whether naked mole-rats can detect IPM, we performed ex vivo electro-olfactogram recordings from the olfactory epithelium of non-breeding individuals (Extended Data Fig. 4a). Exposure to IPM (1:10 v/v in DMSO; equal to 85 µg µl^−1^) elicited short latency field potentials in the olfactory epithelium that were larger than those in the solvent control (DMSO), indicating that the compound activates OSNs (Extended Data Fig. 4b,c).

**Fig. 3:**
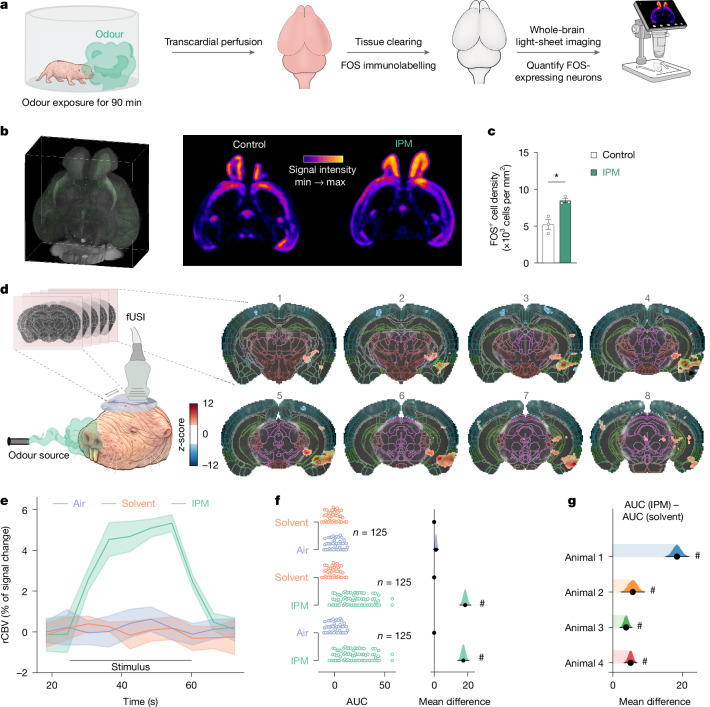
IPM activates olfactory circuits. **a**, Experimental workflow for whole-brain activity mapping. **b**, Representative whole-brain FOS signal intensity maps from control and IPM-exposed animals, shown as maximum-intensity projections. Max, maximum; min, minimum. **c**, FOS^+^ cell density in the olfactory bulb (*n* = 3 animals) showed a significant increase following IPM exposure compared with controls. Individual points denote animals; bars indicate mean ± s.e.m., two-sided unpaired *t*-test; *P = 0.0117. **d**, fUSI during olfactory stimulation in naked mole-rats. Coronal brain sections showing odour-evoked activity (*z*-score) by IPM in one animal (animal 1; 5 sessions). Anatomical overlays from the Allen Mouse Brain Atlas are shown on greyscale power Doppler images. *z*-scores are colour-coded. Statistical thresholds (±3.58; *α* = 0.01, FDR-corrected) were applied; clusters <30 voxels were discarded. A green sphere (*r* ≈ 400 µm) marks the voxel of maximal response. Significant hyperaemia was observed in olfactory-related regions, including the piriform cortex, geniculate nuclei and amygdala-associated areas. **e**, Relative CBV (rCBV) within the green sphere shown in **d** for air, solvent (DMSO) and IPM stimulation. Traces show mean ± s.d. across 5 sessions. **f**, Left, area under the curve (AUC) of rCBV responses for individual voxels (*n* = 125 voxels) across conditions within the green sphere shown in **d**. Right, IPM induces a significant increase in rCBV compared with air and solvent. **g**, Effect sizes and 99% confidence intervals (excluding zero) for DMSO versus IPM across four animals (including data from multiple imaging sessions per animal, *n* = 125 voxels). #, Significant, *P* < 0.01. Source data

We next tested whether exposure of animals to IPM leads to activation of higher-order olfactory centres. Animals were exposed to IPM for 90 min, after which their brains were fixed, optically cleared and immunostained with an antibody against FOS, an immediate early gene that is commonly used as a marker of neuronal activation. We used light-sheet imaging of the entire brain using a mesoSPIM microscope^29^ to image FOS-positive neurons in the olfactory bulb (Fig. 3a). We used a pipeline that enabled us to segment and count FOS-positive neurons in the imaged 3D volume. Animals exposed to IPM exhibited higher FOS^+^ cell density in the olfactory bulb compared with air-exposed controls (Fig. 3b,c).

**Extended Data Fig. 5:**
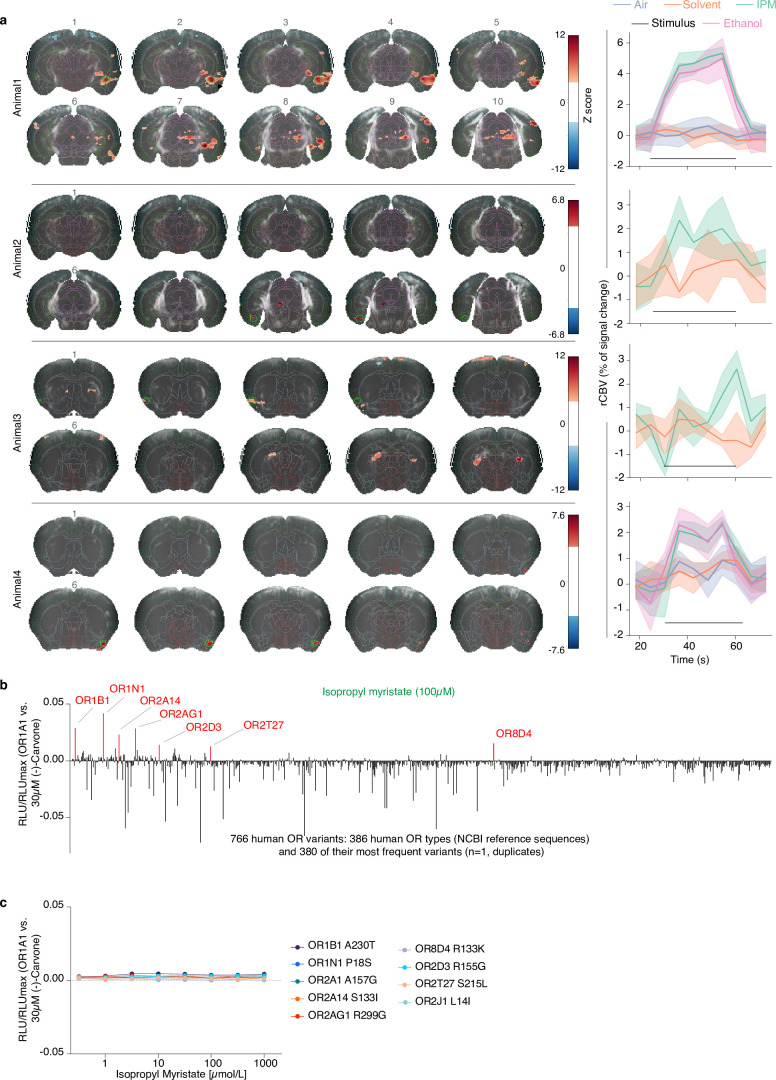
fUSI mapping and human olfactory receptor screening of isopropyl myristate. **a**, Functional ultrasound imaging (fUSI) responses to isopropyl myristate across individual animals. Left, coronal brain sections showing odour-evoked activation maps (Z-scores). Coronal planes show anatomical overlays from the Allen Mouse Brain Atlas on grayscale power Doppler images, with Z-scores indicated in colour. A green sphere (r ≈ 400 µm) marks the voxel of maximal response. Right, relative cerebral blood volume (rCBV; % signal change) within the green sphere for air, solvent (DMSO; negative control), isopropyl myristate, and ethanol (positive control) stimulation. Images represent mean projections across all replicates for each animal. Traces show mean ± s.d. across imaging sessions, averaged over the stimulus period. **b**, A total of 766 recombinant human olfactory receptor (OR) variants, comprising 386 NCBI reference OR types and 380 common natural variants, were screened for responses to 100 μM isopropyl myristate using an IL-6-HaloTag-OR system in HEK-293 cells and a GloSensor cAMP-luminescence assay. Bar graph shows normalized luminescence responses (RLU/RLUmax) relative to 30 μM (–)-carvone, as reference odourant. Several ORs exhibited weak but measurable responses (highlighted in red). **c**, Selected human olfactory receptor variants identified in the initial screen (b) were tested across a range of isopropyl myristate concentrations (1–1000 μM). None of the tested OR variants showed significant dose-dependent activation compared to the reference odourant 30 μM (–)-carvone (OR1A1). Source data

Using FOS as an activity marker could not give us time-resolved information on olfactory responses to IPM. We thus utilized functional ultrasound imaging (fUSI) to measure brain activation during controlled olfactory stimulation in anaesthetized naked mole-rats. The fUS signal is proportional to local cerebral blood volume (CBV) and serves as an indirect readout of neural activity via neurovascular coupling^30^. fUSI enables mesoscopic functional mapping with high spatiotemporal resolution (~100 μm, 80 ms) and has been widely used to measure sensory-evoked activity^30^ including olfactory responses^31^. The non-invasive nature of fUSI enabled us to measure brain-wide responses to olfactory stimuli in the same animals multiple times (Fig. 3d). IPM exposure induced robust, spatially structured CBV increases in olfactory-related brain regions, whereas solvent (DMSO) and air controls produced no detectable responses (Fig. 3d). Notably, responses were lateralized to one hemisphere of the olfactory cortex (Fig. 3d and Extended Data Fig. 5a), which may reflect asymmetric nasal airflow associated with the nasal cycle, an autonomically regulated alternation in nasal cavity patency observed in mammals^32^. Relative CBV changes were stimulus locked and a voxel-wise analysis revealed no significant responses in the same brain areas to air or solvent (Fig. 3e,f). These effects were reproducible across animals, demonstrating that IPM reliably evokes functional hyperaemia in olfactory and limbic circuits (Fig. 3g and Extended Data Fig. 5a).

Notably, IPM has been found in human breath^33^ as well as in the areolar secretions of lactating human mothers^34^. These observations raised the possibility that IPM may be detected by human olfactory receptors, although laboratory members generally perceived IPM as odourless. We thus leveraged a well-annotated repertoire of human odorant receptor variants and screened 766 human odorant receptors using a GloSensor cAMP assay to identify candidates that are responsive to IPM^35^ (100 µM). However, only weak responses were observed in a few receptors, with no significant dose-dependent activation across concentrations (Extended Data Fig. 5b,c). These results suggest that IPM is either undetectable or weakly active on human olfactory receptors. Thus, we could not identify potentially orthologous naked mole-rat olfactory receptors that were activated by IPM.

## IPM blocks reproduction

**Fig. 4:**
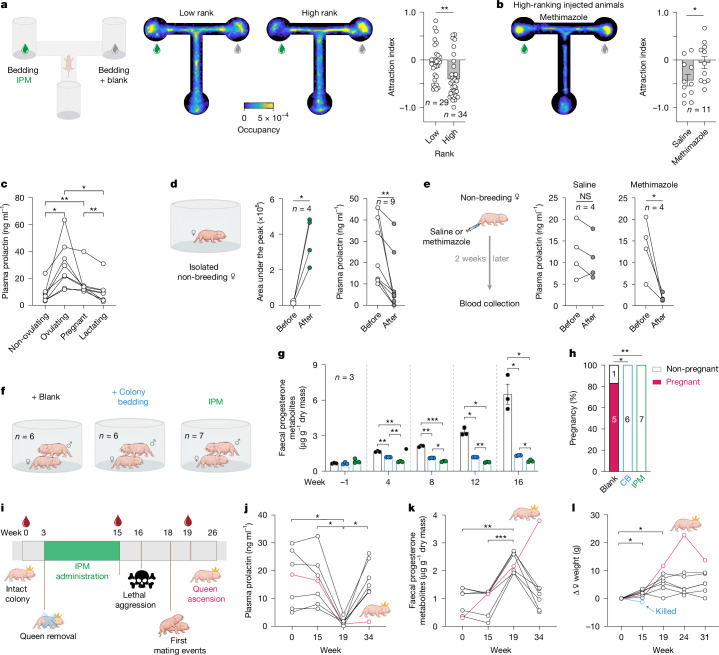
IPM suppresses reproduction and modulates prolactin secretion. **a**, Occupancy maps (middle) and attraction indices (right) for IPM between low-ranking (*n* = 29) and high-ranking (*n* = 34) naked mole-rats. Colour in heat maps indicates the fraction of total time spent at each spatial bin (each map normalized to sum to 1; 0–5 × 10^−^^4^). Two-sided unpaired *t*-test, **P = 0.0019. **b**, Methimazole-treated naked mole-rats did not avoid IPM. Two-sided unpaired *t*-test, *P = 0.045, *n* = 11. **c**, Plasma prolactin levels in non-breeding females (*n* = 8) were higher during queen ovulation or pregnancy. Repeated-measures ANOVA with Greenhouse–Geisser corrections and Tukey’s multiple comparisons test, *P = 0.0196 (non-ovulating versus ovulating), **P = 0.0077 (non-ovulating versus pregnant), *P = 0.0381 (ovulating versus lactating) and **P = 0.0065 (pregnant versus lactating). **d**, Increased IPM (middle; *n* = 4) and decreased prolactin (right; *n* = 9) in females isolated for at least 90 days. Two-sided paired *t*-test, *P = 0.0125 and **P = 0.0081. **e**, Methimazole treatment reduced prolactin levels (*n* = 4). Two-sided paired *t*-test, *P = 0.0359, NS, not significant (*P* ≥ 0.05). **f**, Breeding experiment design. **g**, Reproductive activation without IPM (*n* = 3). Week 4: **P = 0.0088 (IPM versus colony bedding), **P = 0.0067 (IPM versus blank) and **P = 0.0090 (colony bedding versus blank). Week 8: *P = 0.0220 (IPM versus colony bedding), ***P < 0.001 (IPM versus blank) and **P = 0.0032 (colony bedding versus blank). Week 12: **P = 0.0023 (IPM versus colony bedding), *P = 0.0122 (IPM versus blank) and *P = 0.0152 (colony bedding versus blank). Week 16: *P = 0.0122 (IPM versus colony bedding), *P = 0.0443 (IPM versus blank) and *P = 0.0493 (colony bedding versus blank). Two-way repeated-measures ANOVA with the Geisser–Greenhouse correction and Tukey’s multiple comparisons test. **h**, Pregnancy only occured in the blank group. Two-sided Fisher’s exact test, *P = 0.015, **P = 0.0047; blank, *n* = 6; colony bedding (CB), *n* = 6; IPM, *n* = 7. **i**, Key events after queen removal and IPM treatment. Red drops represent blood sampling. **j**, Plasma prolactin levels over time (*n* = 7). Repeated-measures ANOVA with Greenhouse–Geisser corrections and Tukey’s multiple comparisons test, *P = 0.0199 (week 0 versus week 19), *P = 0.0120 (week 15 versus week 19) and *P = 0.0122 (week 19 versus week 34). **k**, Faecal progesterone metabolites over time (*n* = 7). Repeated-measures ANOVA with Greenhouse–Geisser corrections and Tukey’s multiple comparisons test, **P = 0.0021 (week 0 versus week 19) and ***P = 0.0003 (week 15 versus week 19). **l**, Body mass over time (*n* = 8). Repeated-measures ANOVA with Greenhouse–Geisser corrections and Tukey’s multiple comparisons test, *P = 0.0226 (week 0 versus week 15) and *P = 0.0441 (week 0 versus week 19). In **j**–**l**, the pink line represents the finally ascendant queen and the blue line represents an individual that died following an attack by another female. Data are mean ± s.e.m. Source data

**Extended Data Fig. 6:**
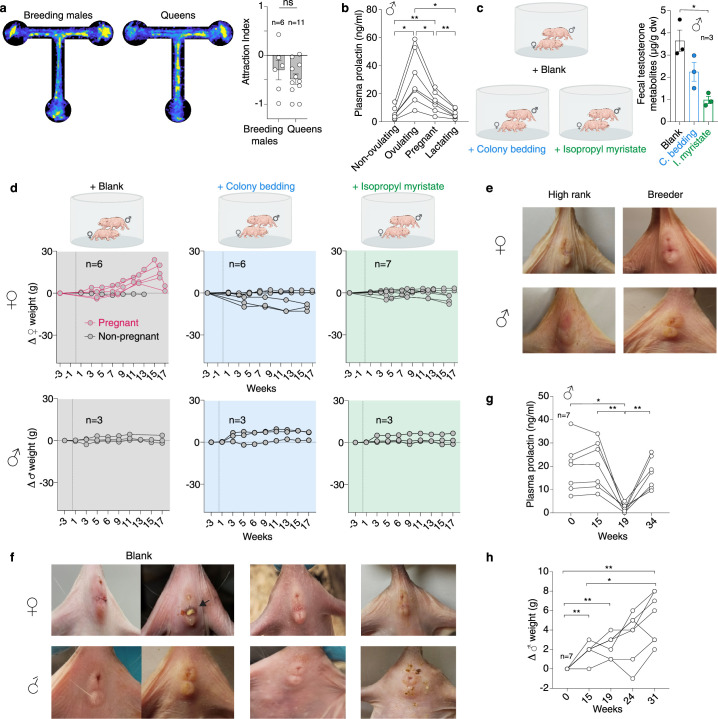
Queen odours and isopropyl myristate suppress reproduction and genital maturation. **a**, Breeding males (n = 6) and queens (n = 11) showed neither attraction nor avoidance of isopropyl myristate. Unpaired t-test, NS *P*>0.05, mean ± SEM. **b**, Plasma prolactin levels in males across the queen’s reproductive states (n = 7). Each line represents an individual; repeated measures ANOVA with Greenhouse–Geisser corrections and Tukey’s multiple comparisons test, *p = 0.0180 (non-ovulating vs. ovulating), **p = 0.0037 (non-ovulating vs. pregnant), *p = 0.0394 (ovulating vs. pregnant), *p = 0.0218 (ovulating vs. lactating), **p = 0.0080 (pregnant vs. lactating). **c**, Faecal testosterone metabolite levels in males housed with blank bedding, colony/queen bedding, or isopropyl myristate (n = 3). Testosterone metabolites were elevated in the blank condition compared to colony bedding or isopropyl myristate; mean ± s.e.m, Kruskal–Wallis test with Dunn’s multiple comparisons, *p = 0.0219. **d**, Body mass changes in isolated opposite-sex pairs housed with blank bedding, colony/queen bedding, or isopropyl myristate. Pregnancy-associated weight gain occurred only in females in the blank condition (5/6), whereas females exposed to colony/queen bedding (0/6) or isopropyl myristate (425 mg) (0/7) show no weight gain. Male body mass was unchanged across all conditions (n = 3). **e**, Breeding males and females displayed qualitative changes in external genital appearance consistent with reproductive status. **f**, Animals exposed to blank bedding developed enlarged genitalia, the arrow indicates material associated with mating. Animals exposed to colony bedding/queen odours or isopropyl myristate retained small genitalia. **g**, Plasma prolactin levels in males (n = 7) dropped by week 19 after isopropyl myristate withdrawal and increased again by week 34 (See Fig. 4i). Repeated-measures ANOVA with Greenhouse–Geisser corrections and Tukey’s multiple comparisons test, *p = 0.0183 (week 0 vs. week 19), **p = 0.0094 (week 15 vs. week 19), **p = 0.0046 (week 19 vs. week 34). **h**, Males gained body mass over time following queen removal (n = 7) (See Fig. 4i). Repeated-measures ANOVA with Greenhouse–Geisser corrections and Tukey’s multiple comparisons test, **p = 0.0090 (week 0 vs. week 15), **p = 0.0075 (week 0 vs. week 19), **p = 0.0090 (week 0 vs. week 31), *p = 0.0255 (week 15 vs. week 31). Source data

Given its queen origin, widespread presence in the colony and ability to activate olfactory circuits in naked mole-rats, IPM is a strong candidate for mediating intra-colony social interactions. To test this, we conducted a two-choice assay (Fig. 4a) in which individuals of different ranks were allowed to freely explore two chambers: one containing bedding scented with IPM (50 µl; 8.5 µg) and another containing untreated bedding, with their behaviours recorded and analysed using video tracking software (Methods). Rank-specific differences were observed as high-ranking individuals showed marked avoidance of IPM, whereas lower-ranking individuals displayed no clear preference (Fig. 4a). Breeding males and queens did not differ significantly, although queens showed a mild trend towards avoidance (Extended Data Fig. 6a). Notably, methimazole-treated higher-ranking individuals no longer avoided IPM, showing that avoidance behaviours required olfaction (Fig. 4b).

The pronounced avoidance of high-ranking individuals to IPM led us to question whether this avoidance behaviour reflects sensitivity to a queen-specific reproductive cue. A hallmark of reproductive suppression in mammals is increased prolactin, which causes infertility and naturally inhibits reproduction during lactation, probably by desensitizing the pituitary gland to gonadotropin-releasing hormone (GnRH)^36,37^. In naked mole-rats, a similar mechanism operates via the central regulation of the hypothalamic GnRH system, involving modulators such as gonadotropin-inhibitory hormone^38–40^. Unlike lower-ranking individuals, higher-ranking animals show reduced prolactin levels and partial recovery of pituitary sensitivity to GnRH, indicating reduced reproductive suppression^41,42^. To assess whether IPM influences neuroendocrine states involved in reproductive suppression, we measured circulating prolactin levels in non-breeding individuals across the queen’s reproductive cycle, when IPM production naturally fluctuates (Fig. 2g). Prolactin levels in non-breeders were significantly higher during the queen’s ovulatory and pregnancy phases, aligning with peak IPM colony-abundance (Fig. 4c and Extended Data Fig. 6b). Of note, non-breeding females that were isolated from their colonies showed reduced prolactin levels and elevated IPM levels (Fig. 4d), and methimazole treatment was associated with reduced plasma prolactin levels (Fig. 4e). These results demonstrate that queen-derived IPM modulates prolactin levels in non-breeders, revealing a potential mechanism by which IPM could mediate reproductive suppression.

To directly investigate the role of IPM in reproductive suppression, non-breeding male and female naked mole-rats were removed from their colonies and housed in opposite-sex pairs (Fig. 4f). Pairs were randomly assigned to one of three treatment groups: (1) a control group, isolated from all colony-derived olfactory cues; (2) a bedding group, isolated but maintained with bedding from their natal colony to preserve queen-associated chemical signals; and (3) an IPM group, isolated and exposed daily to 500 µl of IPM to potentially mimic the queen’s odour. Calibration experiments indicated that the ambient IPM concentration fell to queen levels after 5 h and was not detectable after 24 h (Extended Data Fig. 3d). Following separation from the colony, females typically undergo reproductive activation within 1–3 weeks, followed by a gestation of approximately 10 weeks (refs. ^3,13^). We thus monitored each group for 18 weeks. Consistent with reproductive activation, faecal progesterone metabolite levels increased progressively over time, but only in females housed in the control condition, whereas levels remained low and stable in females exposed to colony bedding or IPM (Fig. 4g). Notably, male faecal testosterone metabolites were highest in the blank condition and reduced by colony bedding or IPM (Extended Data Fig. 6c). Pregnancies occurred only in the control group (5 out of 6 pairs), which also showed clear signs of mating (for example, body mass gain) and reproductive activation, including vaginal perforation (Fig. 4h and Extended Data Fig. 6d–f). By contrast, no pregnancies were observed in the bedding (0 out of 6 pairs) or IPM-treated groups (0 out of 7 pairs), and no signs of mating were detected (Fig. 4h and Extended Data Fig. 6d–f). However, females of the bedding group showed minimal genital changes, but no evidence of vaginal perforation (Extended Data Fig. 6f). Only males in the control group showed enlarged genitalia, confirming reproductive activation (Extended Data Fig. 6f).

Queen removal rapidly triggers heightened, sometimes lethal, aggression in high-ranking females^22,43,44^. These behavioural shifts are closely linked to reproductive activation, marked by vaginal perforation, body mass gain in dominant individuals and modulation of reproductive hormones, all occurring alongside a marked decline in prolactin levels^13,43,45^. Although we demonstrated that IPM is sufficient to suppress reproduction in isolated pairs, it remains possible that a queen odour could stabilize colony dynamics and prevent premature reproductive escalation when a queen is removed. To address this, we implemented a longitudinal intervention in which IPM (500 µl) was administered daily to colony bedding after queen removal (Fig. 4i). Notably, the colony remained socially stable throughout the IPM treatment period of 12 weeks: no aggression or dominance contests were observed, and plasma prolactin levels in non-breeders remained high, closely matching the levels seen in intact colonies (Fig. 4j). Consistent with continued reproductive suppression, faecal progesterone metabolite levels remained low during IPM exposure, indicating a lack of ovarian activation (Fig. 4k). The prolonged nature of this hormonal and behavioural stability (12 weeks) indicated that under these experimental conditions, IPM was able to mimic reproductive suppression and social stability that is normally associated with the presence of a queen.

Upon cessation of IPM treatment, the colony rapidly destabilized. Circulating prolactin levels declined significantly within the first month (Fig. 4j), coinciding with a marked increase in faecal progesterone metabolite concentrations and the emergence of sexual behaviour in multiple females (Fig. 4i,k). One week after IPM withdrawal (week 16), high-ranking individuals began exhibiting aggression, and a lethal attack occurred, resulting in the death of one female (Fig. 4i). By week 19, a surviving dominant female showed a marked increase in body mass, a known correlate of reproductive ascension, and became pregnant (Fig. 4l, pink line). This individual also exhibited a sharp drop in plasma prolactin and a concomitant increase in faecal progesterone, reaching levels typical of established breeders (Fig. 4j,k, pink line) and gave birth. Together, these findings demonstrate that IPM not only suppresses reproduction, but can also stabilize social structure in naked mole-rat colonies.

## Discussion

**Fig. 5:**
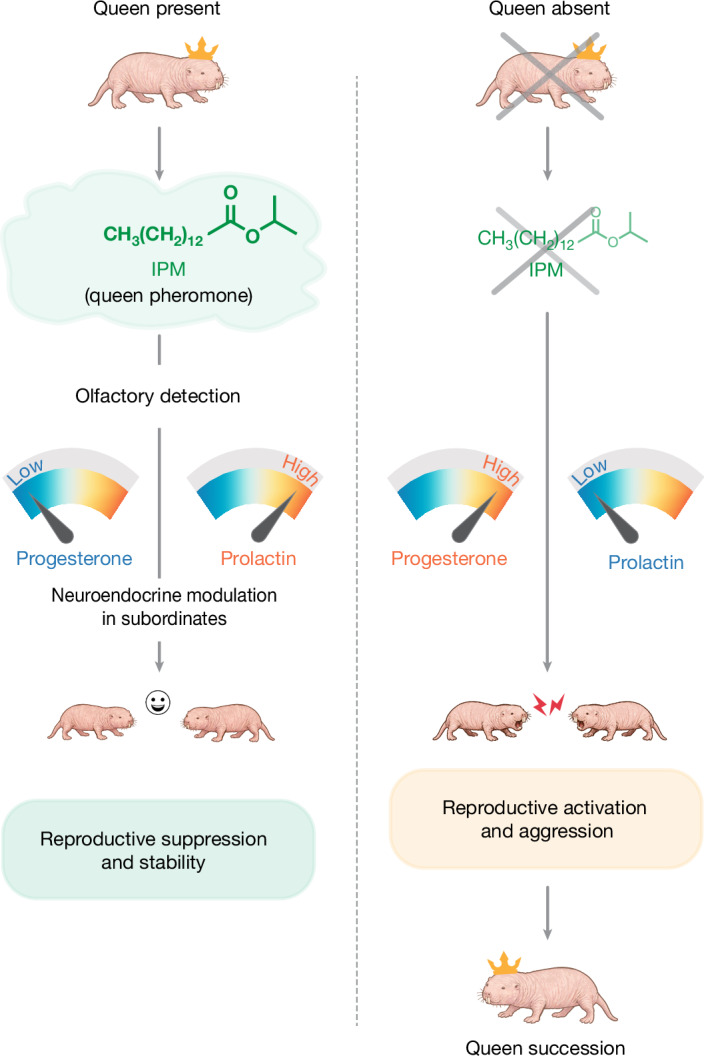
IPM links queen presence to prolactin elevation and progesterone suppression. Schematic illustrating contrasting social and physiological outcomes in naked mole-rat colonies in the presence (left) and absence (right) of the queen. When the queen is present, she produces IPM, which is detected via olfaction and increases prolactin while suppressing progesterone in non-breeders, maintaining reproductive suppression and social stability. Upon queen loss, IPM levels decline, prolactin decreases and progesterone increases, triggering reproductive activation and aggression that can result in mortality. Ultimately, one dominant female ascends to reproductive status, starts to produce IPM and succeeds as the new queen.

Here we identify IPM as a queen-enriched compound in naked mole-rats that mediates reproductive suppression and stabilizes colony structure (Fig. 5). Our findings suggest a functional mammalian analogue of insect eusociality: a stable, queen-derived signal with colony-wide physiological and behavioural effects, echoing the role of queen pheromones in insects^11,12^. By linking olfactory perception to prolactin-driven infertility, we show that a queen-enriched compound can in principle coordinate physiology and behaviour across the colony. Using several methodological approaches including non-invasive fUSI brain imaging we demonstrate that IPM elicits robust activation of olfactory neurons and central circuits providing a neural substrate for its colony-wide effects. Our data are consistent with detection of IPM via the main olfactory epithelium, however, we cannot exclude a contribution of the vomeronasal system.

IPM production peaks during ovulation, is detected by olfactory circuits, and increases circulating prolactin to induce infertility in naked mole-rats. This mirrors the established role of prolactin in lactational infertility across mammals^36,37,46^, suggesting that naked mole-rats have co-opted an ancient endocrine pathway for social regulation. High-ranking animals who maintain lower prolactin and partial pituitary sensitivity to GnRH^41^ showed robust avoidance, linking them to ‘beta queens’ with latent reproductive potential^44^. This rank-specific sensitivity shows how IPM shapes both endocrine states and social behaviour. Consistently, ablation of olfaction eliminated aggression initiation and reduced prolactin levels, directly tying IPM detection to reproductive suppression (Fig. 5).

Queen removal, which normally precipitates aggression, reproductive competition and prolactin decline^13,43,45^, was instead followed by months of stability when IPM was added to the colony bedding. During this period, faecal progesterone metabolite levels remained low, indicating sustained ovarian quiescence alongside increased prolactin. It remains unclear whether the effects of IPM generalize to natural conditions or to rare cases of colonies with more than one breeding female^5^. Similarly, isolated pairs exposed to the compound did not reproduce, whereas controls readily conceived. Our findings also resolve a long-standing puzzle from earlier work showing that, although reproductive suppression in naked mole-rats requires the queen’s presence and is lifted by her removal or death^1,13,43^, daily transfer of soiled bedding and urine from the parent colony does not maintain suppression in isolated individuals^47,48^. This contradiction may be explained by the chemical nature of IPM, its fluctuation across the queen’s reproductive stages and its near-absence from urine. As a long-chain fatty acid ester (C_17_ backbone; molecular mass of approximately 270 g mol^−1^) with relatively low volatility, IPM may be ineffective over long distances, which may in turn explain the queen’s increased activity and extensive spatial coverage of the colony^25,49,50^.

Comparative analyses revealed that IPM was detected in breeding females of a further four *Fukomys* species (Fig. 2i). The mechanisms of reproductive suppression probably vary between these four *Fukomys* species, but physiological suppression of reproduction with considerable reproductive skew, similar to naked mole-rats, has been described in *Fukomys damarensis*^51^.

Although queens and breeding males are continuously exposed to IPM, they remain reproductively active and do not self-suppress. This paradox could be explained by molecular and cellular brain plasticity associated with attaining breeder status. Indeed, morphological and molecular changes have already been described to occur in breeding versus non-breeding animals^52^, and it is plausible that these changes could, for example, involve differential expression of prolactin receptors across various key brain regions after queen ascension^53^. However, further work should determine whether rank-specific differences in odorant receptor sensitivity or downstream signalling mediate selective suppression.

The properties of IPM differ from the complex odour blends typical of cooperative mammals such as marmosets^9^ or wolves^10^. Instead, we propose that it functions as a long-lived signal that is capable of maintaining endocrine suppression and social quiescence even in queenless colonies, effectively acting as a ‘social hormone’. Its deposition at multiple body sites and transfer via queen-specific behaviours such as tunnel rubbing and ‘pass over’ interactions^42,49^ probably ensures persistent colony-wide exposure, whereas partial avoidance by beta females may allow succession.

These results provide a rare example of a chemical signal with colony-wide effects in a mammal. However, we cannot exclude the contribution of other sensory or behavioural mechanisms in maintaining reproductive suppression. Further work will be required to disentangle how this chemosensory signal—and potentially others—interact with behavioural and social cues, which are likely to act in a coordinated way to regulate reproductive hierarchy in natural colonies. We propose that IPM activates an olfactory–hypothalamic pathway, increasing prolactin levels to inhibit GnRH release^54^, and thereby coupling odour detection to reproductive control, paralleling olfactory inputs to hypothalamic reproductive circuits described in other mammals^55,56^. Future work should investigate the biosynthesis of IPM, its receptor pathways, and whether comparable odorant regulators exist in other cooperative vertebrates. The involvement of this molecule in reproductive suppression highlights a fundamental principle of social evolution: a queen pheromone that can regulate reproductive hierarchies, prevent conflict and sustain eusociality in a mammal.

## Methods

### Animals and housing

Long-term laboratory colonies of naked mole-rats (*H. glaber*) were maintained in Berlin, Germany and Pretoria, South Africa. Damaraland mole-rat (*F. damarensis*) colonies were maintained in Pretoria, and Micklem’s mole-rat (*Fukomys micklemi*), Mashona mole-rat (*Fukomys darlingi*) and Mechow’s mole-rat (*Fukomys mechowii*) were maintained in České Budějovice, Czech Republic. (The following species were kept in the laboratory (Pretoria, South Africa) for unrelated studies after being collected from the wild: Highveld mole-rat (*Cryptomys hottentotus pretoriae*), Natal mole-rat (*Cryptomys hottentotus natalensis*), common mole-rat (*Cryptomys hottentotus hottentotus*), Cape mole-rat (*G. capensis*), Cape dune mole-rat (*B. suillus*), and Emin’s mole-rat (*H. emini*).

In Berlin, naked mole-rats were kept under controlled environmental conditions, with ambient temperature kept at 30–32 °C and humidity at 50–70%, under dim illumination. Eighteen colonies were housed in custom-designed, interconnected plastic chamber systems (Fräntzel Kunststoffe). Animals were provided with a daily ad libitum diet of tubers (primarily sweet potatoes, celery root, cucumbers, bananas and carrots) and supplemented weekly with ProNutro (Bokomo). All husbandry and experimental procedures were approved by the local governmental authorities in Berlin (Landesamt für Gesundheit und Soziales, licenses G 0196/17 and G 0121/23). When selecting animals for experiments, a non-invasive salivary swab assay was established to determine sex by genotyping. In brief, saliva was collected from live naked mole-rats using a swab (Geyer). The extraction of genomic DNA was performed with proteinase K. Precipitation of the DNA was performed to concentrate the DNA solution used as template for the PCR. To determine whether the animal is a male, we amplified a 163-bp region of the sex-determining gene on the Y chromosome, *Sry* using Hg-SRY-For 5′-GAAGAACGGCCATTTTTCGG-3′ Hg-SRY-Rev 5′-GCATTCATGGTGTGGTCTCG-3′.

To check the DNA quality and PCR condition, we amplified a 446-bp region of the mitochondrial 16S rRNA gene using Hg-16S-For, 5′-TGGTGATAGCTGGTTGTCCA-3′ and Hg-16S-Rev, 5′-TAGTCTTTCCTTGCGGCACT-3′, amplicon detectable in both sexes as previously described^57^.

In South Africa, eight colonies of naked mole-rats were maintained in tunnel systems consisting of multiple plastic chambers (designated for food storage, toileting and nesting) connected by acrylic glass tunnels. Animals were fed a varied diet of chopped vegetables (primarily sweet potatoes, cucumbers and carrots) with weekly ProNutro supplementation. Nesting material consisted of wood shavings. The holding rooms were kept at 29–32 °C with 50–70% relative humidity.

In the Czech Republic, the animal rooms of Micklem’s mole-rat, Mashona mole-rat and Mechow’s mole-rat were kept at 25 ± 1 °C, 50 ± 10% relative humidity and photoperiod of 12 h light:12 h dark. Animals were fed ad libitum with vegetables (such as carrots, potatoes, sweet potatoes, beetroot, apple and cucumbers) and a rodent dry food mix. Animals were given the opportunity to carry out their natural digging behaviours in peat and were provided extra enrichment such as tree branches and plastic tubes for gnawing.

All procedures were approved by the relevant institutional animal care and use committees and local governmental authorities in Berlin (Max Delbrück Center; Landesamt für Gesundheit und Soziales, licenses G 0196/17 and G 0121/23) and Pretoria (University of Pretoria), in accordance with national and international guidelines for the ethical treatment of laboratory animals. Procedures in Pretoria were approved by the Animal Ethics Committee of the University of Pretoria (license no. NAS199/2020, NAS071-2023, NAS313/2022, NAS324/2022, NAS209-2021, NAS011/2025 and NAS130_2025) and received DALRRD Section 20 approval (SEpi-Bizhub24110620262, SDAH-Epi-22101309360, SDAH-Epi-23032315040, SDAH-Epi-23041710040, 12/11/1/1/8 (2002 LH), SEpi-Bizhub25021311032 and SEpi-Bizhub25061009490). All naked mole-rats used in this study were derived from colonies originally captured by J. Jarvis, primarily in Mtito Andei and Lerata, Kenya, and represent a mixed parentage^1^.

### Behavioural assays

#### Social rank determination

To assess dominance hierarchies within the eight colonies from South Africa, we followed a previously established assay for naked mole-rat social rank^22,58^. In brief, two plastic chambers were connected by a transparent tube, with one animal placed in each chamber. When both entered the tube simultaneously, the individual climbing over the other was scored as dominant. Each dyad was tested in at least three trials, with pairings pseudo-randomized and repeated across multiple months. A ranking index (R.I.) was calculated as wins divided by total trials and normalized to the colony maximum (R.I. = 1 for the top-ranking individual). Ranks were assigned categorically (rank 1: R.I. > 0.8, rank 5: R.I. < 0.2). For experiments, we used high-ranking individuals with R.I. ≥ 0.7 and low-ranking individuals with R.I. ≤ 0.3.

#### Social recognition and sniffing discrimination

Pairs of naked mole-rats were introduced into transparent acrylic tubes and video-recorded with a high-speed camera (BASLER, boA1936-400cm) at 100 frames per second for 5 min. Sniffing bouts, characterized by rhythmic nose movements and defined as nose-to-body contact, were scored by an observer blind to colony identity. Each dot represents one animal-pair interaction.

#### Aggression assays

Animals from seven colonies from South Africa were paired and introduced into a rectangular arena (60 cm × 7 cm). Aggressive interactions—including head-to-head lunges, shoves and bites—were video-recorded, and head-to-head positions (distances <4 cm) were analysed using a custom-written MATLAB script in combination with DeepLabCut^59^. All frames were subsequently checked by human observers, and any errors were corrected to ensure accurate position identification. Recordings lasted 5 min or were terminated earlier if there was a risk of severe injury. An aggression index was calculated as the proportion of time an animal engaged in aggressive behaviours relative to the total assay duration. Spatial distributions of head-to-head encounters were visualized as heat maps. Higher-ranking individuals, previously identified to defend their colonies in observations across these seven colonies, were used.

#### T-maze odour preference assay

Odour-guided attraction was tested in a two-choice T-tube olfactometer. The apparatus comprised a start chamber (30 cm × 20 cm × 14 cm) connected to 2 entrance tunnels (64 cm × 7 cm), which extended into side tunnels (80 cm × 7 cm) leading to either a control or a stimulus chamber (30 cm × 20 × 14 cm). In the food odour assay, one arm was scented with ProNutro (cereal-based supplement) food odour (odour source placed outside the maze to prevent contact), whereas the opposite arm contained no odour. In the IPM preference assay, bedding was treated with IPM diluted in DMSO (in total 50 µl; 8.5 µg) in one arm and 50 µl DMSO as a solvent control in the other. Animals were placed in the start chamber and allowed to explore freely for 10 min. Position and movement trajectories were recorded using a custom-written MATLAB script in combination with DeepLabCut^59^. Attraction indices were calculated as (time in odour arm – time in control arm)/total assay time. To control for side bias, the odour-stimulus arm was alternated between replicates.

#### Maze exploration

Individuals were placed in a novel multi-arm maze (80 cm × 7 cm, 4 arms) and video-recorded for 15 min. Animal trajectories were quantified and total coverage was calculated.

#### Pregnancy-suppression assay

Opposite-sex pairs were formed from low-ranking non-breeders, matched for body size and drawn from the same colonies to avoid confounds related to isolation history, and were housed in three chambers (30 cm × 20 cm × 14 cm) connected by two tunnels (64 cm × 7 cm). Pairs were randomly assigned to blank bedding (*n* = 6), bedding supplemented with toilet bedding from their original colony (*n* = 6), or bedding treated daily with IPM (*n* = 7; 500 µl applied to bedding, equivalent to 425 mg; see Extended Data Fig. 3c,d for time-dependent decay of IPM). Because testes in naked mole-rats remain intra-abdominal, external genital morphology was used only as a qualitative indicator and not as a quantitative measure of gonadal activation. Animals were monitored for 18 weeks, during which pregnancy status, body weight, genital morphology, vaginal perforation and reproductive activation were assessed; reproductive activation was assessed using endocrine measures and qualitative external genital observations, including vaginal perforation, which were documented photographically. Pregnancy was defined by progressive body mass gain of ~20–30 g beginning at weeks 3–9 and continuing until weeks 15–18. In the blank group (3 replicates in Berlin, 3 in Pretoria), 5 of 6 females gained body mass and gave birth, whereas none of the females in the colony bedding (3 Berlin, 3 Pretoria) or IPM groups (3 Berlin with 500 µl isopropyl myristate + 500 µl DMSO, 4 Pretoria with 500 µl neat isopropyl myristate; both delivering ~425 mg IPM) became pregnant and body masses remained stable. Pregnancy outcomes were interpreted as a downstream consequence of reproductive activation and were evaluated alongside longitudinal endocrine measures and body mass trajectories. Males showed no significant body mass changes in any group.

#### Queen removal and colony stability

Three weeks after blood collection from an intact colony, the queen was removed from an intact colony that consisted of 19 individuals (8 females and 11 males) to initiate destabilization, after which IPM (500 µl equal to 425 mg; see Extended Data Fig. 3d for time-dependent decay of IPM) was applied daily to the bedding for 12 weeks to simulate persistent queen-derived olfactory cues. The colony was inspected daily, and behavioural interactions were scored. Blood samples were collected at weeks 0, 15, 19 and 34 by tail venipuncture, and plasma was stored at –80 °C until analysis. Prolactin concentrations were measured as described below. Body masses of all females were measured weekly using a precision balance (Scout Pro SPU123, Ohaus).

### Odour collection and chemical analyses

Volatile compounds were collected by placing individual naked mole-rats in a Plexiglas chamber (30 cm × 20 cm × 14 cm) for 30 min, during which headspace odours were adsorbed onto PDMS tubes suspended from the chamber lid. PDMS tubing (1 mm internal diameter × 0.4 mm wall thickness; VWR International) was cut into 1-cm segments, soaked in 100% methanol for up to 24 h, and conditioned under a constant flow of purified nitrogen at 180 °C for 1.5 h in a modified heating oven. Odour samples were collected from non-breeding males, non-breeding females, breeding males, and queens across reproductive states (pre-mating, mating, pregnancy and lactation (*n* = 3 for each)). Ovulation was inferred retrospectively using established reproductive staging in naked mole-rats, based on longitudinal behavioural observations, and defined reproductive states (pre-mating, mating, pregnancy, and lactation). As direct determination of the ovulatory window was not feasible, this classification reflects inferred reproductive state rather than precise measurement of ovulation. Additional samples were non-invasively obtained from eight African mole-rat species, and in naked mole-rat queens, swabs were taken from vaginal, anal, oral, teat, and skin regions using PDMS tubes on the same day.

Breeding females of the four *Fukomys* spp. were sampled across two reproductive stages (ovulation and pregnancy), as were naked mole-rats. The three *Cryptomys* spp. females were likewise sampled during ovulation and pregnancy, whereas *Bathyergus* females were sampled during lactation. We could not identify the reproductive stage of *G. capensis*. Importantly, IPM was detectable across all sampled reproductive stages (Fig. 2g), but its abundance was most strongly elevated during ovulation, indicating that the observed species differences are not attributable to mismatched reproductive staging. Reproductive state in all breeding females was retrospectively determined using established morphological and behavioural criteria.

All samples were analysed by TD-GC–MS as previously described^23^ with minor modifications. In brief, analyses were performed on an Agilent 7890A GC system coupled to a 5975C inert XL MSD and fitted with an HP5-MS UI column (19091S-433UI; Agilent Technologies). Following desorption at 250 °C for 3 min, volatiles were cryo-trapped at −50 °C with liquid nitrogen and transferred to the GC column via a vaporizer injector heated to 270 °C (12 °C s^−1^, 5-min hold). The GC oven program was: 40 °C for 3 min, ramped at 5 °C min^−1^ to 260 °C (10-min hold), then 5 °C min^−1^ to 280 °C (5-min hold). Mass spectrometry parameters were: transfer line 260 °C, ion source 230 °C, quadrupole 150 °C. Compounds were ionized by electron impact (70 eV), detected in positive ion mode (*m*/*z* 33–500), and identified using NIST spectral libraries. XCMS (v3.7.1) was used to analyse and compare odour profiles. The identity of IPM was confirmed by comparison of mass spectra and retention time with a commercial standard (Sigma-Aldrich, 172472).

#### Calibration of IPM on PDMS tubes

To generate a calibration curve, defined amounts of IPM (1 µl of 8.5 ng, 850 ng, 85 µg, and 850 µg; Sigma-Aldrich) were applied directly onto conditioned PDMS tubes (1 cm × 0.3 cm). After solvent evaporation, tubes were analysed using TD-GC–MS (Agilent 7890 A/5975 C, equipped with an HP-5MS UI column). Peak areas of the IPM chromatographic signal were quantified and log_10_-transformed. A linear regression of log_10_(peak area) versus log_10_(mass) was fitted (*Y* = 0.6016 × *X *+ 8.656). The mean peak area measured from queen samples was converted to mass equivalents using this calibration, corresponding to 660 ± 80 ng IPM (mean ± s.d., *n* = 4–7).

#### Time-course decay of IPM in a chamber

For breeding and queen-removal experiments, we examined the temporal dynamics of volatilized IPM, 500 µl (425 mg) of neat IPM was placed on the floor of a sealed Plexiglas chamber (30 cm × 20 cm × 14 cm). PDMS tubes were suspended from the chamber lid and collected volatiles at defined timepoints (0, 6, 12, 18 and 24 h). Tubes were analysed by TD-GC–MS as described above. Peak areas were log_10_-transformed and plotted against time, revealing a progressive decline in IPM signal. Linear regression yielded *Y* = –0.05602 × *X* + 8.827. The average queen IPM level corresponded to the concentration present at 5.2 ± 0.75 h after application (*n* = 4).

### Methimazole-induced olfactory ablation

Methimazole (Sigma-Aldrich; M8506) was diluted in 0.9% NaCl and administered by intraperitoneal injection at 75 mg kg^−1^. Following methimazole injury, the olfactory epithelium in mice regenerates over approximately 1 month, with newly generated OSNs beginning to establish synaptic contacts with the olfactory bulb within ~1–2 weeks (ref. ^60^). Accordingly, all behavioural experiments were conducted within 3–7 days post-injection, the established window of maximal OSN loss, ensuring effective olfactory ablation. Control animals received sterile saline only. After 72 h, olfactory epithelium was collected for histological analysis as described^18^. In brief, animals were perfused, and tissue was fixed in 4% paraformaldehyde (PFA), embedded in OCT, cryosectioned at 60 µm, and immunostained with antibodies against OMP (mature OSNs; goat polyclonal, 1:1,000; Wako Chemicals, 544-10001) and STMN1 (immature OSNs; rabbit polyclonal, 1:500; Abcam, ab24445). Images were acquired using an Airyscan confocal fluorescence microscope (Axio, Zeiss) with a 20× objective. Exposure parameters were kept constant across sections, and identical contrast adjustments were applied to all images. Mean fluorescence intensity was quantified using ImageJ^61^.

### Electro-olfactogram recordings

Recordings were performed as previously described^62^ with minor modifications. Animals were deeply anaesthetized with an overdose of ketamine and xylazine (100 and 20 mg kg^−1^, respectively) and subsequently decapitated. Heads were bisected along the sagittal midline and immediately transferred to a dissecting microscope, where the olfactory epithelium was surgically exposed. Extracellular activity from OSNs was recorded using a tungsten wire electrode inserted into the olfactory mucosa. For stimulus delivery, 10 μl of freshly prepared odorant solution was applied to a 1 cm^2^ piece of filter paper (Whatman) and inserted into a glass Pasteur pipette. Odour stimuli (1 s air puffs) of IPM (1:10 dilution in DMSO; equal to 85 µg µl^−1^) or DMSO alone were delivered at a controlled flow rate of 40 cm s^−1^ (Stimulus Controller CS 55, Ockenfels-Syntech). Signals were amplified at a sampling rate of 10 kHz, low-pass filtered at 50 Hz using a NeuroLog Amplifier (Digitimer), and recorded with a PowerLab 4/30 system (ADInstruments) running LabChart 8 software for subsequent analysis.

### Whole-brain FOS imaging

Animals were exposed to IPM (500 µl, equivalent to 425 mg) or solvent for 90 min in a Plexiglas chamber (30 cm × 20 cm × 14 cm). At the end of exposure time, animals were deeply anaesthetized with an intraperitoneal single-dose injection of ketamine and xylazine (100 and 20 mg kg^−1^, respectively), transcardially perfused with phosphate-buffered saline (PBS) followed by 4% PFA. Whole-brain tissue was collected, incubated with 4% PFA overnight at 4 °C and then transferred to 0.02% sodium azide in PBS for storage until clearing. One brain was excluded from the FOS analysis because unsuccessful perfusion compromised tissue quality and prevented reliable quantification. All other samples were processed according to the LifeCanvas Technologies protocol (v5.03, based on^63^) in the following order: SHIELD preservation, Delipidation with SmartClear II Pro, Immunolabelling with SmartLabel. Cleared brains were incubated with 3.5 μg of rabbit anti-FOS monoclonal antibody (Abcam, ab214672) together with 24 μl of propidium iodide (Thermo Fisher, P3566, 1.0 mg ml^−1^ solution in water) for nuclear stain. Primary antibody against FOS was fluorescently conjugated (Alexa Fluor® 647) with a donkey anti-rabbit secondary antibody (Jackson ImmunoResearch, 711-605-152) in 1:1.5 primary:secondary molar ratio. After active labelling, samples were matched to a refractive index of *n* = 1.52 using EasyIndex (LifeCanvas Technologies). Two datasets were generated, each containing equal numbers of animals from both treatment conditions. The first dataset (*n* = 4) was processed as described above and imaged with a custom built mesoSPIM^29^ light-sheet fluorescence microscope using 4× magnification. Pixel size was 1.6 µm for nominal lateral spatial resolution with a 6 µm step size in the *z*-direction. 488, 561 and 647 nm excitation was used with the respective 520/50, 590/50 and quad band 405/488/561/640 nm emission filters to image the entire brain for autofluorescence background, nuclear stain and FOS signal. The second dataset (*n* = 2) was sent to LifeCanvas Technologies where samples were passively delipidated for 7 days, batch labelled using SmartBatch+ with donkey anti-rabbit SeTau647 secondary antibody (LifeCanvas Technologies, DkxRb-ST), and imaged with SmartSPIM using a 3.6× objective. Pixel size was 1.8 μm laterally with a 4 μm *z*-step. Laser excitation at 561 nm and 640 nm was used with 600/50 and 690/50 emission filters to image nuclear stain and FOS signal. All samples in each dataset were imaged using the same imaging protocol and excitation intensities.

### Whole-brain image processing and analysis

FOS^+^ neurons in the olfactory bulb were quantified using a difference-of-Gaussian filter and local maxima detection implemented in Matlab 2023b (Mathworks). Whole-brain images were stitched and reconstructed using BigStitcher and Fiji^64,65^. All analyses were performed blinded to treatment conditions.

To enable quantitative comparison of FOS-positive cell densities acquired using different light-sheet microscopes, we assessed and matched the effective spatial resolution of mesoSPIM and SmartSPIM datasets. Intensity profiles along the axial (*z*) dimension were extracted from individual FOS-positive cells and fitted with Gaussian functions to estimate the axial point spread function (PSF). SmartSPIM data exhibited substantially higher axial resolution than mesoSPIM. To harmonize resolution, SmartSPIM image stacks were filtered with a 3D Gaussian kernel (*σ* = 1, 1 and 2 pixels in *x*, *y* and *z*, respectively), preserving lateral resolution while increasing axial spread to match that of the mesoSPIM. Filtered SmartSPIM datasets were then resampled to the mesoSPIM voxel size prior to downstream cell counting and density analyses. Importantly, filtering parameters were derived solely from single-cell PSF measurements and were independent of cell counts.

The total volume of the olfactory bulb from which FOS-positive cells were quantified was estimated by manually thresholding the light-sheet fluorescence imaging data. The threshold was selected to include all visible olfactory bulb tissue while excluding background signal. The number of voxels exceeding this threshold was multiplied by the voxel volume to obtain the total sampled volume. FOS^+^ cell density was calculated as the number of FOS-positive cells divided by the total sampled olfactory bulb volume (cells per mm^3^).

### fUSI experiment

Plane wave fUSI was performed using an Iconeus One ultrafast ultrasound system (Iconeus) equipped with a 15-MHz IcoPrime 4D MultiArray probe. Whole-brain volumes were acquired by scanning 40 coronal planes with a slice spacing of 0.21 mm, using the manufacturer’s default ultrafast plane-wave sequence. Experiments were performed in naked mole-rats aged 1–3 years with body weights not exceeding 40 g. Animals were anaesthetized with ketamine (100 mg kg^−1^) and xylazine (2 mg kg^−1^) and secured in a stereotaxic frame. Rectal body temperature was maintained at 29.5 °C using a heating pad placed beneath the animal. Odour delivery was controlled by a stimulus controller (CS 55, Ockenfels-Syntech) connected to a glass tube, from which gas was released at the distal end (2 mm diameter). The glass tube was positioned in front of the anterior nostrils to allow efficient delivery of odorants into the nasal cavity. Each imaging session lasted 6 min and followed an off–on stimulation paradigm consisting of alternating 30-s stimulation and 30-s rest periods.

### Signal analysis of fUSI data

Although no anatomical atlas is currently available for the adult naked mole-rat, we found that overall brain size and major vascular architecture resemble those of the mouse. Therefore, imaging data were registered to the Allen Mouse Brain Atlas^66^. Following an initial anatomical registration using Iconeus’ proprietary Brain Positioning System (BPS)^67^, a second, animal-specific registration step was performed. For each animal, a randomly selected imaging session was chosen as a reference, and all remaining acquisitions from that animal were registered to this reference session, thereby inheriting the transformation matrix linking the data to atlas space. Registration was conducted in two stages—coarse and fine—using custom Python code implemented with SimpleITK registration utilities and methods^68^.

### First-level event-based analysis with generalized linear modelling

As previously described, olfactory stimulation was performed using a single event-based experimental design. For statistical analysis, we used previously developed custom code^69^ that adapts a generalized linear model framework^70^ from fMRI Python packages^71^ for fUSI data. Power Doppler images were first spatially smoothed using a Gaussian kernel (full width at half maximum, 300 µm). A design matrix was then constructed incorporating the stimulus paradigm, cosine drift regressors, and data-driven confounding regressors. The stimulus pattern was convolved with a previously described hemodynamic response function^72^. Cosine drift terms were computed up to 0.01 Hz based on the power Doppler acquisition frequency (1/6 Hz). Data-driven confounds were estimated using aCompCor^73^ by extracting the first three principal components of the Power Doppler signal from white matter and cerebrospinal fluid voxels. Using a first-order autoregressive model^74^, a generalized linear model was fitted for all imaging sessions in a given pair of animal and experimental condition (for IPM, DMSO (negative control), ethanol (positive control) and, when available, air olfactory stimulation). *z*-score maps were derived from model effect sizes using stimulus-specific contrast matrices. Statistical thresholds were determined using the Benjamini–Hochberg procedure for false discovery rate correction (*α* = 0.01), and voxels not forming 6-connected clusters of at least 30 voxels above threshold were excluded. Finally, prominent clusters in thresholded IPM *z*-maps were identified, and spherical regions of interest (*r* ≈ 400 µm) were defined around the voxel exhibiting the maximal *z*-score.

### rCBV in the piriform cortex

Following cluster detection in the *z*-score maps, one spherical mask per animal (*n* = 4) was selected to encompass a prominent activation cluster located within or adjacent to the piriform cortex. For each animal, power Doppler time series were extracted from all voxels within the corresponding mask for each experimental condition (IPM, DMSO and air). For each imaging session within a given condition, rCBV signals were obtained by regressing out previously computed data-driven aCompCor confounds from the extracted power Doppler time series using QR decomposition, followed by standardization of the residual signals as percent change relative to baseline. The baseline was defined as the first OFF period of the stimulation paradigm and the second half of subsequent OFF periods. For each rCBV time series, the average stimulus-evoked response was computed by windowing the stimulus ON period, padded at both onset and offset by half of the OFF period, and then averaging these windows across imaging sessions for each experimental condition.

### Bootstrap and permutation testing

Permutation testing^75^ was performed separately for each animal to compare rCBV responses between pairs of experimental conditions. For each voxel within the spherical mask, the mean trapezoidal AUC of the rCBV signal was calculated over the stimulus ON period. The difference between mean AUC values for condition A (for example, IPM) and condition B (for example, DMSO) was then computed. Effect sizes and 99% confidence intervals were estimated using 5,000 permutation resamples, in which voxels were randomly reassigned to conditions A or B with replacement. Permutation testing was implemented using the DABEST Python package^76^.

### Plasma collection and prolactin measurement

Adult naked mole-rats were removed from their natal tunnel systems and weighed. Venous blood samples, corresponding to ~1% of the animal’s body mass were collected from the tail vein using heparinized micro-haematocrit tubes and transferred into Eppendorf tubes. Samples were centrifuged immediately at 500*g*, and the resulting plasma was decanted and stored at −80 °C until analysis. Plasma prolactin concentrations were quantified using a commercial enzyme-linked immunosorbent assay (ELISA; Elabscience, guinea pig prolactin kit, E-EL-GP0358) following the manufacturer’s protocol. This assay has been previously validated for naked mole-rats^38^. The intra-assay coefficient of variation was <10%, and assay sensitivity was 0.09 ng ml^−1^.

### Faecal sample collection

The opposite-sex pairs experiments were conducted across two locations (as stated above). Faecal hormone analyses were performed only on samples collected in South Africa (*n* = 3 pairs). Faecal samples were collected opportunistically during routine animal management procedures, including scheduled weighing and blood sampling events. Samples were collected immediately after defecation to avoid environmental contamination, transferred into sterile containers using sterilized forceps, and stored frozen until further processing.

### Faecal steroid extraction

Faecal samples were freeze-dried and homogenized to a fine powder following established protocols^42^. Depending on sample mass (0.0150–0.0249 g, 0.0250–0.0366 g and 0.0370–0.0550 g), steroids were extracted using 0.5, 1.0 or 1.5 ml of 80% ethanol, respectively. Samples were vortexed for 15 min, centrifuged at 1,500*g* for 10 min, and the resulting supernatant was transferred into microcentrifuge tubes^77^. Extracts were stored at −20 °C until hormone analysis.

### Quantification of faecal androgen and progestagen metabolites

Faecal androgen metabolites and faecal progestagen metabolites were quantified using enzyme immunoassays (EIAs) for epiandrosterone^78^ and 5α-tetrahydroprogesterone^79^, respectively. These assays have been validated for use in naked mole-rats and shown to reliably reflect reproductive endocrine activity^80^. It should be noted that faecal steroid metabolites reflect circulating hormone changes with a physiological delay, as they represent metabolized hormone excretion rather than real-time endocrine levels.

Hormone quantification followed established protocols^81^. Assay sensitivities were 6.0 ng g^−1^ faecal dry mass for the 5α-tetrahydroprogesterone EIA and 7.2 ng g^−1^ faecal dry mass for the epiandrosterone EIA. Intra-assay coefficients of variation were 4.38% and 5.32% for the 5α-tetrahydroprogesterone EIA and 7.40% and 7.94% for the epiandrosterone EIA. Inter-assay coefficients of variation for the 5α-tetrahydroprogesterone EIA were 11.10% and 12.75%. Only a single plate was used for the epiandrosterone EIA; therefore, inter-assay variation was not calculated.

### Human odorant receptor screening and luminescence assay

HEK-293 cells (RRID:CVCL_0045), a human embryonic kidney cell line, were used as a heterologous system for recombinant olfactory receptor expression. Cells were maintained at 37 °C in a humidified incubator (5% CO_2_) in DMEM supplemented with 4.5 g l^−1^ d-glucose, 10% fetal bovine serum, 2 mM l-glutamine, 100 U ml^−1^ penicillin and 100 μg ml^−1^ streptomycin. Luminescence-based functional assays were performed as previously described^35^. For receptor screening, a cDNA expression plasmid library comprising 766 constructs, encoding 386 human odorant receptor reference sequences and 380 common variants, was transfected into HEK-293 cells.

cAMP-dependent luminescence signals were acquired using a GloMax Discover detection system (Promega) and analysed in Excel (Microsoft, v16.109.2). Raw traces were averaged, and basal activity was subtracted from odorant-induced responses. For concentration–response relationships, baseline-corrected values were normalized to the maximal response of a reference odorant receptor–odorant pair, and signals from mock-transfected controls were subtracted. Dose–response curves and half-maximal effective concentration (EC_50_) values were determined by nonlinear regression (SigmaPlot 14.0, Systat Software) using the equation *f*(*x*) = (min – max)/(1 + (*x*/EC_50_)^Hillslope^)+max, with Hill slope constrained to ≤2.9 and data points weighted by the reciprocal of their standard deviation (0.1/s.d.). Dose–response curves were fitted using nonlinear regression (log[agonist] versus response, variable slope).

### Phylogenomic analysis

Protein-coding transcripts from ten African mole-rat species (*H. glaber*, *H. emini*, *G. capensis*, *F. damarensis*,* F. micklemi*, *F. darlingi*, *F. mechowii*, *C. hottentotus*, *C. natalensis* and *C. pretoriae*) were combined with transcripts obtained from published data^82^ and from a newly assembled transcriptome of *B. suillus*. Following previous methodology^82^, orthologous datasets were cleaned, resulting in a set of 3,393 single-copy genes (SCGs) shared across all 11 species. These genes were aligned using the mafft-linsi algorithm implemented in MAFFT^83^, and alignments were further trimmed to remove columns containing gaps in more than 50% of the species. Orthofinder^84^ was used to discover orthogroups and build a species tree from the SCGs. Maximum-likelihood gene trees and species trees were inferred using default settings (-m MSA) based on the individual gene trees. For divergence time estimation, the species tree was calibrated with the TimeTree R package^85^, constraining the split between *M. musculus* and Bathyergidae to 56–77 million years ago.

### Statistics and figure preparation

Sample sizes were chosen on the basis of feasibility, ethical considerations and consistency with prior studies in naked mole-rats and other mole-rats, as well as established practice for behavioural, endocrine, imaging and electrophysiological experiments in this field. No statistical methods were used to predetermine sample size. Where possible, multiple independent animals, colonies or experimental repetitions were used. Opposite-sex pairs in the pregnancy-suppression assay were randomly assigned to blank bedding, natal colony bedding or IPM. Other groups were defined by social/reproductive status or colony manipulation, so randomization was not applicable. Behavioural and FOS analyses were performed and scored by observers blinded to treatment. Blinding was not applicable where groups were defined by social or reproductive status or by chemical or colony treatment. Normality was first assessed on datasets using a Shapiro–Wilk test. Statistical analyses (see the corresponding legends of each figure) and figures were generated using GraphPad Prism v8 (https://www.graphpad.com) and further processed with Adobe Illustrator v25.2.1.

### Reporting summary

Further information on research design is available in the Nature Portfolio Reporting Summary linked to this article.

## Online content

Any methods, additional references, Nature Portfolio reporting summaries, source data, extended data, supplementary information, acknowledgements, peer review information; details of author contributions and competing interests; and statements of data and code availability are available at 10.1038/s41586-026-10772-5.

## Supplementary information

Supplementary Table 1 Supplementary Table 1 | List of identified compounds in naked mole-rat (shown in Extended Data Fig. 2c), including retention time, chemical class, CAS numbers and identification confidence (probability %).

Reporting Summary

Peer Review file

Supplementary Video 1 Sniffing interaction between two animals from the same colony. Colony mates engage in brief reciprocal sniffing.

Supplementary Video 2 Sniffing interaction between two animals from different colonies. Two naked mole-rats from different colonies engage in reciprocal sniffing.

Supplementary Video 3 Sniffing behaviour in a T-maze. A naked mole-rat explores odour cues (food odours) and displays directional sniffing in the T-maze assay.

Supplementary Video 4 Aggressive interaction between two saline-treated animals. Saline-injected naked mole-rats from two different colonies engage in head-to-head shoving and biting.

Supplementary Video 5 Interaction between two methimazole-treated animals. Methimazole-injected naked mole-rats from two different colonies show absence of aggression compared to saline controls.

Supplementary Video 6 Aggressive interaction between saline- and methimazole-treated animals. Saline-treated animals initiate aggression, while methimazole-treated animals respond defensively.

## Source data

Source Data Figs. 1–4 and Extended Data Figs. 1–6

## Data Availability

The raw data of GC–MS, fUSI and FOS light-sheet imaging and the phylogenetic tree generated in this study are available at 10.17617/3.7QCU1F. Behavioural videos are available from the authors upon reasonable request. All data supporting the findings of this study are available within the paper and its Supplementary Information files. Source data are provided with this paper.

## Code availability

Custom codes (except fUSI analysis pipeline) for analysis of chemical profiles (XCMS), FOS light-sheet imaging, and behavioural T-maze data are available at 10.17617/3.7QCU1F.
