# Supplementary material for: A queen odour mediates reproductive suppression in a eusocial mammal
Source: Nature. 2026 Jul 15;656(8127):390–8. doi: 10.1038/s41586-026-10772-5 (PMC13468116; doi:10.1038/s41586-026-10772-5)
Supplement: Supplementary file 1 [file 41586_2026_10772_MOESM2_ESM.pdf]

nature portfolio

Corresponding author(s): Mohammed A. Khallaf
Gary R. Lewin

Last updated by author(s): Jun 1, 2026

Reporting Summary

Nature Portfolio wishes to improve the reproducibility of the work that we publish. This form provides structure for consistency and transparency in reporting. For further information on Nature Portfolio policies, see our Editorial Policies and the Editorial Policy Checklist.

Statistics

For all statistical analyses, confirm that the following items are present in the figure legend, table legend, main text, or Methods section.

n/a	Confirmed	
☐	☒	The exact sample size (*n*) for each experimental group/condition, given as a discrete number and unit of measurement
☐	☒	A statement on whether measurements were taken from distinct samples or whether the same sample was measured repeatedly
☐	☒	The statistical test(s) used AND whether they are one- or two-sided<br>*Only common tests should be described solely by name; describe more complex techniques in the Methods section.*
☒	☐	A description of all covariates tested
☐	☒	A description of any assumptions or corrections, such as tests of normality and adjustment for multiple comparisons
☐	☒	A full description of the statistical parameters including central tendency (e.g. means) or other basic estimates (e.g. regression coefficient) AND variation (e.g. standard deviation) or associated estimates of uncertainty (e.g. confidence intervals)
☐	☒	For null hypothesis testing, the test statistic (e.g. *F*, *t*, *r*) with confidence intervals, effect sizes, degrees of freedom and *P* value noted<br>*Give P values as exact values whenever suitable.*
☒	☐	For Bayesian analysis, information on the choice of priors and Markov chain Monte Carlo settings
☐	☒	For hierarchical and complex designs, identification of the appropriate level for tests and full reporting of outcomes
☒	☐	Estimates of effect sizes (e.g. Cohen's *d*, Pearson's *r*), indicating how they were calculated

Our web collection on statistics for biologists contains articles on many of the points above.

Software and code

Policy information about availability of computer code

Data collection	Data collection used BASLER boA1936-400cm cameras for behavioural videos; an Iconeus One ultrafast ultrasound system with IcoPrime 4D MultiArray probe and manufacturer acquisition software for fUSI; MesoSPIM and SmartSPIM (LifeCanvas Technologies) for light-sheet microscopy; Agilent 7890A GC/5975C inert XL MSD for TD-GC-MS; PowerLab 4/30 with LabChart 8 (ADInstruments) for electro-olfactogram recordings; and GloMax Discover (Promega) for luminescence assays.
Data analysis	Data analysis used custom MATLAB scripts with DeepLabCut for tracking/pose estimation; ImageJ/Fiji, BigStitcher and BigDataViewer for light-sheet image processing/visualization; MATLAB 2023b for c-Fos cell detection and signal processing; custom Python code with SimpleITK, nilearn and DABEST for fUSI GLM/permutation analyses; Excel (Microsoft, V16.109.2) and SigmaPlot 14.0 for OR assays; GraphPad Prism v8 for statistics; Adobe Illustrator v25.2.1 for figures; and MAFFT, OrthoFinder and the TimeTree R package for phylogenomics. Custom codes, except the fUSI pipeline, are available at https://doi.org/10.17617/3.7QCU1F.

For manuscripts utilizing custom algorithms or software that are central to the research but not yet described in published literature, software must be made available to editors and reviewers. We strongly encourage code deposition in a community repository (e.g. GitHub). See the Nature Portfolio guidelines for submitting code & software for further information.

Data

Policy information about availability of data

All manuscripts must include a data availability statement. This statement should provide the following information, where applicable:

- Accession codes, unique identifiers, or web links for publicly available datasets
- A description of any restrictions on data availability
- For clinical datasets or third party data, please ensure that the statement adheres to our policy

Raw GC-MS, fUSI, c-Fos light-sheet imaging and phylogenetic tree data generated in this study are available at https://doi.org/10.17617/3.7QCU1F. Source data are provided with the paper, and all data supporting the findings are within the paper and Supplementary Information. Behavioural videos are available from the authors upon reasonable request. Custom codes, except the fUSI analysis pipeline, for c-Fos light-sheet imaging and behavioural T-maze analyses are available at the same DOI. The Allen Mouse Brain Atlas was used for image registration as described in Methods.

Research involving human participants, their data, or biological material

Policy information about studies with human participants or human data. See also policy information about sex, gender (identity/presentation), and sexual orientation and race, ethnicity and racism.

Reporting on sex and gender	NA
Reporting on race, ethnicity, or other socially relevant groupings	NA
Population characteristics	NA
Recruitment	NA
Ethics oversight	NA

Note that full information on the approval of the study protocol must also be provided in the manuscript.

Field-specific reporting

Please select the one below that is the best fit for your research. If you are not sure, read the appropriate sections before making your selection.

☒ Life sciences ☐ Behavioural & social sciences ☐ Ecological, evolutionary & environmental sciences

For a reference copy of the document with all sections, see nature.com/documents/nr-reporting-summary-flat.pdf

Life sciences study design

All studies must disclose on these points even when the disclosure is negative.

Sample size	Sample sizes were chosen based on feasibility, ethical considerations, and consistency with prior studies in naked mole-rats and other mole-rats, as well as established practice for behavioural, endocrine, imaging, and electrophysiological experiments in this field. Where possible, multiple independent animals, colonies, or experimental repetitions were used, and sample sizes are comparable to or exceed those reported in previous studies addressing similar questions. Exact sample sizes (n), numbers of biological replicates, and numbers of independent experiments are reported in the figure legends and Methods. One brain from the c-Fos experiment was excluded from analysis because the animal was not perfused correctly. No other data were excluded from the analyses.
Data exclusions	One brain from the c-Fos experiment was excluded from analysis because the animal was not properly perfused leading to high background. No other data were excluded from the analyses.
Replication	Independent biological replicates are reported in figure legends and Methods. Pregnancy suppression was replicated in Berlin and Pretoria (blank bedding n=6: 3 Berlin/3 Pretoria; colony bedding n=6: 3/3; isopropyl myristate n=7: 3/4). The queen-removal intervention was performed in one colony.
Randomization	Opposite-sex pairs in the pregnancy-suppression assay were randomly assigned to blank bedding, natal-colony bedding or isopropyl myristate. Other groups were defined by social/reproductive status or colony manipulation, so randomization was not applicable.
Blinding	Behavioural and c-Fos analyses were performed and scored by observers blinded to treatment. Blinding was not applicable where groups were defined by social/reproductive status or by chemical/colony treatment.

Reporting for specific materials, systems and methods

We require information from authors about some types of materials, experimental systems and methods used in many studies. Here, indicate whether each material, system or method listed is relevant to your study. If you are not sure if a list item applies to your research, read the appropriate section before selecting a response.

Materials & experimental systems

n/a	Involved in the study
☐	☒ Antibodies
☐	☒ Eukaryotic cell lines
☒	☐ Palaeontology and archaeology
☐	☒ Animals and other organisms
☒	☐ Clinical data
☒	☐ Dual use research of concern
☒	☐ Plants

Methods

n/a	Involved in the study
☒	☐ ChIP-seq
☒	☐ Flow cytometry
☒	☐ MRI-based neuroimaging

Antibodies

Antibodies used	OMP (olfactory marker protein), goat polyclonal, Wako Chemicals, cat. no. 544-10001, dilution 1:1000. STMN1 (stathmin-1), rabbit polyclonal, Abcam, cat. no. ab24445, dilution 1:500. Rabbit anti-c-Fos monoclonal antibody, Abcam, #ab214672, 3.5 ug; donkey anti-rabbit secondary antibody, Jackson ImmunoResearch, 711-605-152, primary:secondary molar ratio 1:1.5. Nuclear stain: propidium iodide, Thermo Fisher, P3566.
Validation	OMP and STMN1 were used as markers of mature and immature olfactory sensory neurons, respectively; staining showed expected loss after methimazole. c-Fos was used as an immediate early gene marker of neuronal activation, and c-Fos-positive cells were quantified in control and isopropyl myristate-exposed brains.

Eukaryotic cell lines

Policy information about cell lines and Sex and Gender in Research

Cell line source(s)	HEK-293 cells (RRID:CVCL_0045), a human embryonic kidney cell line, were used as a heterologous system for recombinant olfactory receptor expression.
Authentication	NA
Mycoplasma contamination	NA
Commonly misidentified lines (See ICLAC register)	NA

Animals and other research organisms

Policy information about studies involving animals; ARRIVE guidelines recommended for reporting animal research, and Sex and Gender in Research

Laboratory animals	Species: naked mole-rat (Heterocephalus glaber); Damaraland mole-rat (Fukomys damarensis); Micklem's mole-rat (Fukomys micklemi); Mashona mole-rat (F. darlingi); Mechow's mole-rat (F. mechowii); Highveld mole-rat (Cryptomys h. pretoriae); Natal mole-rat (C. h. natalensis); common mole-rat (C. h. hottentotus); Cape mole-rat (Georychus capensis); Cape dune mole-rat (Bathyergus suillus); Emin's mole-rat (Heliophobius emini). Source: long-term laboratory colonies in Berlin, Pretoria and Ceske Budejovice; some species were kept in the laboratory after wild collection for unrelated studies. Age/sex: both sexes and all social/reproductive states; fUSI animals were <=40 g.
Wild animals	The following species were kept in the laboratory for unrelated studies after collection from the wild: Highveld mole-rat, Natal mole-rat, common mole-rat, Cape mole-rat, Cape dune mole-rat and Emin's mole-rat.
Reporting on sex	Both sexes were included. Analyses were not sex-restricted unless explicitly stated in the Methods.
Field-collected samples	NA
Ethics oversight	All procedures were approved by relevant institutional animal care and use committees and local governmental authorities in Berlin (Max Delbruck Center; LAGeSo licenses G 0196/17 and G 0121/23) and Pretoria (University of Pretoria). Pretoria approvals: NAS199/2020, NAS071-2023, NAS313/2022, NAS324/2022, NAS209-2021, NAS011/2025 and NAS130_2025; DALRRD Section 20 approvals: SEpi-Bizhub24110620262, SDAH-Epi-22101309360, SDAH-Epi-23032315040, SDAH-Epi-23041710040, 12/11/1/1/8 (2002 LH), SEpi-Bizhub25021311032 and SEpi-Bizhub25061009490.

Note that full information on the approval of the study protocol must also be provided in the manuscript.

Plants

Seed stocks	NA
Novel plant genotypes	NA
Authentication	NA
