# Supplementary material for: A queen odour mediates reproductive suppression in a eusocial mammal
Source: Nature. 2026 Jul 15;656(8127):390–8. doi: 10.1038/s41586-026-10772-5 (PMC13468116; doi:10.1038/s41586-026-10772-5)
Supplement: Supplementary file 2 [file 41586_2026_10772_MOESM3_ESM.pdf]

nature portfolio

Peer Review File

A queen odour mediates reproductive suppression in a eusocial mammal

Corresponding Author: Professor Gary Lewin

Any redactions in this file are there to maintain patient confidentiality, the confidentiality of unpublished data, or to remove third-party material.

This file contains all reviewer reports in order by version, followed by all author rebuttals in order by version.

Version 0:

Reviewer comments:

Referee #1

(Remarks to the Author)
Khallaf and co-authors describe a single queen-derived molecule, isopropyl myristate, that has the power to control reproductive behavior in two species of eusocial naked mole rats. This is a fascinating and compelling story with many points of strength. It solves a long-standing problem in the field, and links themes from eusocial insects to this new discovery in eusocial mammals. The authors show that social interactions require the olfactory system (Figure 1), identify a single molecule (isopropyl myristate) that is specific to queens and specific to reproductive stage (Figure 2), that the molecule activates the olfactory system (Figure 3 – though see my serious concern below), that isolated non-breeder females begin to produce this molecule, and that the molecule or colony bedding will inhibit mating (Figure 4), and finally that in a real colony situation that the molecule will enforce a phase of peaceful behavior after queen removal and that its withdrawal triggers conflict and the eventual emergence of a new queen (Figure 4).

There are a few points that the authors should address to further strengthen the work:

1. Figure 1 is not a strong start to the paper and doesn't really address the core question of the queen odor – it's just a demonstration that olfaction matters for aggression behavior. I would consider reorganizing the narrative to talk about the search for the queen substance by opening with Figure 2, which is very strong.

2. Figure 3 is a weak demonstration that isopropyl myristate acts through the olfactory system and is the weakest point in an otherwise fantastic paper. This is probably no fault of the authors. Isopropyl myristate is extremely non-volatile and it's not clear that the response in the EOG (Figure 3a-c) is materially different than the response to DMSO. The fact that the authors do not show the EOG response to DMSO is telling. The data points in Figure 3c show that for most experiments, isopropyl myristate is not different than DMSO. For the c-fos experiment, the authors use clean air rather than DMSO as the control. Therefore there is no way to disambiguate whether the (weak) c-fos signal is due to DMSO, or isopropyl myristate, or a combination. My recommendation is NOT to ask the authors to redo these difficult experiments, but simply remove these data and these claims from the paper entirely. It's possible that some different method of applying the odors or another concentration would show olfactory responses, but it does not seem worth sacrificing more precious animals and more time of the scientists involved to do this experiment. By common sense readers will believe that this molecule is likely acting on the olfactory system, and the very compelling data in Figure 4 are persuasive that it is a molecule that has the power to disrupt endocrinology, mating, colony structure, etc. This is enough at this stage for this story!

Minor points
Line 9-10 – this seems speculative as a reason for high-ranking animals to avoid the odorant

Line 70 – just curious if the methimazole injection itself causes lethargy that might be contributing to the reduced aggression behavior? Or were there control experiments to establish that the effect is specific to ablating the olfactory system? I see that Extended Data Fig 1 has some good countering points that would be good to call out in the Results for Figure 1

Line 86 – what is the food odor? The methods do not provide adequate information (Line 690-691)

Line 90, Line 97-107 – is this an aggression assay where both are either saline or methimazole treated, or are you comparing the aggressiveness of a saline vs. methimazole animal? In Figure 1d, are these animals of the same or different ranks? The issue of social rank is not well explained in the behavioral assays. This is not clear from the figure or text, and as it's a key experiment it is necessary to be very clear about what the experimental design is.

Line 117 (Figure 2e-g) – the use of bars and stars to indicate statistical differences is not clear, I suggest the alternate approach of labeling each set of data with a different letter if they are statistically different.

Line 140-142, Figure 2h, Lines 174-178, Lines 396-397 – this experiment seems like a non-sequitur in this otherwise highly compelling and persuasive figure. While it is convincing that the queens explore the maze more than subordinates, it is an overinterpretation to conclude that this is because she is scent marking. There could be other neural/behavioral differences between queens and subordinates that are driving the differences in behavior, independent of their ability to produce isopropyl myristate. Also it was not clear if the authors measured whether isopropyl myristate was physically deposited by the queen, so the statement (Line 396-397) that she is doing 'tunnel rubbing' is not substantiated by data.

Line 161-170, Line 904 (Extended Data Fig 3e) – these are important data that should be moved to the main Figure 2, which has plenty of space for these exciting data. This result greatly strengthens the case that isopropyl myristate is the molecule of eusociality.

Line 179 (Figure 3b) – would the authors please provide example traces of responses to solvent? The differences between DMSO and isopropyl myristate are not extremely compelling in Figure 3c

Line 179 (Figure 3d-e), Line 211-215 – given that the EOG responses of DMSO and isopropyl myristate are not extremely different, it is problematic that the controls for the c-fos experiment were simply air-exposed rather than DMSO-exposed. It is therefore not possible to interpret how much of the c-fos immunoreactive cells are specifically activated by isopropyl myristate vs the DMSO solvent.

Line 216-226 – it's impressive that the authors went looking for a human isopropyl myristate receptor, but I do not think that the negative result is particularly interpretable. It is possible that such a receptor exists, but it does not show functional expression in the assay being used. To avoid disrupting the flow of the paper with this interesting non-sequitur, I would recommend moving all of this text to the methods and out of the main paper (but retaining it as it's an interesting set of negative data for experts in the field).

Line 241 (Figure 4c) – as mentioned above, it would be best to indicate significance using the notation of different letters indicating significance rather than the many bars and stars.

Review prepared by Leslie Vosshall

Referee #2

(Remarks to the Author)
This interesting study investigates the potential role of a queen derived chemosignal in reproductive suppression in naked mole-rats (NMRs), in the form of a single compound (isopropyl myristate). Beyond the significance in furthering our understanding of NMR sociality, the results of the study could have broad impact in the field of mammalian pheromones, the latter being the subject of considerable debate. For example, the characterisation of mammalian pheromones has remained elusive, even in mice, for which research has spanned many decades (see Wyatt 2014 for review) – with some authors even doubting their existence in mammals (in the social-insect sense of the definition), e.g. Doty (2010). To date, dominance-based behavioural interactions between the NMR queen and non-breeders have provided some explanation regarding the cues that bring about suppression, with a general agreement among NMR researchers that an individual odour "signature" (and possibly vocal cues) from the queen reinforces her presence in the darkness of the subterranean niche. This study goes further than this conjecture in implicating a single chemical rather than a learned odour profile. Many aspects of the study are robustly, logically and appropriately conducted and presented in a clear fashion. However, some aspects need much further clarification and justification and there are some weaknesses in the approach. In particular, the measures of reproductive activation of non-breeders lack detail/nuance (and even plausibility), and the relative contributions of the odour signal versus queen dominance interactions in suppression could or should be explored further. More care needs to be taken in social definitions and extrapolation across other species in the family. These and other issues are covered in more detail below.

Specific points (minor)
1. Line 7: isopropyl myristate is stated to be volatile, yet in other places in the manuscript it is stated as low volatility (e.g. line 171).
2. Lines 15-16: "both eusocial species" only the NMR has been mentioned thus far in the abstract, this needs clarifying so it makes sense. Also see further discussion below regarding the validity of the statement "this mechanism enables mammalian eusociality".
3. Lines 24-25: "division of labor into specialized roles" isn't strictly part of the accepted categorical definition of eusociality proposed by Michener, 1969.
4. Lines 33-34: the statement "In mammals, such cues can trigger profound reproductive consequences, including

pregnancy disruption (the Bruce effect)8, suppression of ovulation in subordinates9," is slightly misleading regarding Reference 9 (Barrett et al. 1990) and would benefit by being re-worded. Barrett et al. found a short extension of reproductive suppression (time to commencement of ovarian cyclicity) in subordinate female marmoset monkeys separated from their social groups but maintained in contact with the scent of the dominant breeder. Other cues (visual and behavioral) were also crucial in mediating full suppression (see follow on study by Barrett et al. 1993) as subordinate females with ablated olfactory epithelia within social groups stay suppressed, diminishing further the role of odour cues.

5. Line 48: isopropyl myristate was focused on as a prime candidate for a chemosignal presumably because in Figure 2c it is shown to be the most enriched compound in queens, although it could be argued that less enriched compounds could have greater effect depending on the sensitivity of the olfactory epithelium of NMRs to particular compounds. Notwithstanding that, "unknown 2" was also highly enriched. Why was that not chemically characterized and tested as well? If nothing else, it could have constituted a good control compound. A full tabulated list of compounds identified (as illustrated in extended data Fig. 2) would also be a useful addition in the supplementary section.

6. Line 53: The statement "uncovering a fundamental mechanism underlying mammalian eusociality" is arguably rather strong given that by the strict definition of eusociality (which many researchers disagree with using for mammals) the are only two "eusocial mammals" – both mole-rats (NMR and Damaraland mole-rats) and the role of isopropyl myristate in the latter is far from proven (See point 15 below). I suggest toning down the wording.

7. Lines 56-57: This ability has been tested before e.g. Clarke and Faulkes, 1999

8. Lines 64-66: How long does the OSN ablation last – is it permanent?

9. Lines 97-100: This description of the division of labor is outdated and not that simple – see Mooney et al., 2015, Gilbert et al., 2020, Holmes and Goldman 2021.

10. Lines 157-158 (and Fig. 2g legend): "Levels peaked during ovulation" - How was the time of ovulation determined in these females in order to collect the samples? This isn't explained (unless I missed it).

11. Lines 161-163: "Naked mole-rats and Damaraland mole-rats (Fukomys damarensis) are the only known eusocial mammals" this depends on your definition, see Crespi and Yanega (1995), among others.

12. Lines 164-165: "versus over 30% in Damaraland mole-rats" – check the 30%. I'm sure a much lower figure has been quoted before.

13. Lines 165-168: Again, it is difficult not to become bogged down with definitions like "social" versus eusocial (see point 11 above). There is a spectrum of sociality among Cryptomys and Fukomys, with overlap in terms of group sizes etc, and also evidence for physiological suppression (again, see point 15 below).

14. Section starting line 196: I see why the odour receptor activation test had to be done, but in and of itself the results are perhaps not surprising as presumably many of the chemicals found (in extended Fig. 2) would potentially bind to an olfactory receptor and relay information to the olfactory bulb. That's how the sense of smell works. The vestigial VNO of NMRs is well known yet this is where a less volatile signal would normally be expected to act, and the pathways running to the accessory olfactory bulb then relaying information to the reproductive centres of the hypothalmus. If isopropyl myristate despite being "of low volatility" is acting via the primary olfactory epithelium and primary olfactory bulb, then this is highly unusual in terms of the accepted neural circuitry. Some mention of this might be helpful or worth brief discussion.

Specific points (major)

15. Lines 165-170: "Remarkably, isopropyl myristate was exclusively detected in the two eusocial mole-rat species, H. glaber and F. damarensis, but was absent in all other tested African mole-rat species (Extended Data Fig. 3e)." While this statement might be true, it doesn't tell the whole story, and more information and qualification of this statement is required. Firstly, the mechanism of suppression in F. damarensis females is different to the NMR (Bennett et al. 2022) and not prolactin driven, so any pathways of action of isopropyl myristate must be different in damarensis. More importantly, while several species of Cryptomys were screened for isopropyl myristate (and was found to be absent) only F. damarensis was examined among Fukomys. Within that genus the are many "social" species including a number that are very close sister species to F. damarensis. The authors contentions would be much stronger (and more believable) if several other Fukomys species were screened for the presence of isopropyl myristate to prove that it has only evolved as a signal in F. damarensis, and not for example in the common ancestor of the Fukomys clade.

Furthermore, on lines 48-49 it is stated that "isopropyl myristate, is uniquely present in queens, especially peaking in vaginal and anal secretions during reproductive readiness". Were the other species of mole-rat analysed for isopropyl myristate and illustrated in Fig. 3e also at these stages of their reproductive cycle? If not, what was their reproductive status? Presumably it could be missed. How many individuals of these other species were analysed? What sexes were they? None of this important information is given for these other eight species in the methods (line 737).

Janse van Vuuren et al. (2025) suggest a prolactin-mediated physiological-based socially induced infertility in highveld mole-rats (C. pretoriae) – the same as in NMRs. C. pretoriae are included among the other mole-rats screened for isopropyl myristate, yet was not found, arguing against a widespread function among mole-rats. This should be mentioned.

16. Line 285 onwards to the discussion and 702-716: In my opinion, the measures of reproductive activation in the separation and scent/bedding transfer experiments are not detailed enough and some measures are unconvincing at best. Granted, pregnancy appears to be suppressed. However, important subtleties are lost. Firstly, the authors state "reproductive activation (testis enlargement, vaginal perforation, copulatory plug formation) was documented photographically". How can testis enlargement be measured in a small rodent that does not have an external scrotal sac? The testes in NMRs remain deeply abdominal and do not descend. In non-breeder males they are average mass of 0.034 g/testis - even in breeders they are very small (testis mass around 0.079 g/79 mg) and could surely only be measured by a highly sensitive ultrasound device. How could testes enlargement possibly have been "documented photographically"? Can the measurements be provided? Furthermore, while occasionally a crusty vaginal discharge can be seen in queens, copulatory plugs as described for other mammals (to prevent sperm competition) do not form in NMRs (Seney et al. 2009). External genitalia is a difficult way of assessing either sex in NMRs, and vaginal development and perforation can also be very variable among females in a colony - separated singly housed females can initially undergo clear ovarian cycles (indicated by elevated progesterone) yet remain imperforate with no male present and thus no mating. The photos in Extended Data Fig. 5e are not overly convincing without "before" and "after" separation pictures for all the animals concerned and some kind of quantitative

measure. Without this it is all very subjective.
Without hormonal profiling (e.g. testosterone in males and progesterone in females) or post-mortem gonadal histology in the experimental (bedding transfer/isopropyl myristate) treatment conditions to look at gonadal activation it is impossible to tease apart sex differences and/or more subtle yet important effects of suppressing cues. For example, one scenario could be that the separated females in scent contact began ovarian cycling (i.e. were released from suppression) yet did not become pregnant because just the males were suppressed by the isopropyl myristate. Reading the paper cited on line 375 (Faulkes et al., 1993), of the bedding transfer experiments reported therein only one of the six treatment females actually became pregnant (91 days after pairing with a male), yet all the separated treatment females receiving bedding transfer showed ovarian cyclicity (as evidenced by progesterone profiles). This emphasises the disadvantages of relying on pregnancy as the only clear marker of reproduction in the manuscript under review. Why were hormonal profiling or post-mortem gonadal histology omitted from the study? It seems a major and obvious omission.
It is also not clear how two-queen colonies can form (although uncommon, they do occur) with females sharing the nest and in close body (and chemical) contact. Likewise, how does the queen recruit a breeding male from a non-breeder when characteristically they spend much time in close body/chemical contact?
On lines 285-287 it states “Prolactin levels in subordinates were significantly higher during the queen’s ovulatory and pregnancy phases, aligning with peak isopropyl myristate colony-abundance (Fig. 4c)” but the figure legend states this was only in females – this needs clarifying. What about males? Previous work suggests that as the queen approaches the end of gestation she may lose some control of the colony (in terms of suppression) – what stage of pregnancy is reflected in 4c? Higher prolactin would infer increased suppression and be at odds with these earlier observations.
On lines 295-301 it states that “Pairs were randomly assigned to one of three treatment groups”, but how were the male-female pairs initially chosen for treatment assignment in terms of social rank, body size, home or different colony? All the aforementioned could affect pairing success. Was it andom as well. As worded, it is not clear.
On lines 305-307 - see comment above regarding measures of activation and plugs, and note on line 309 that mating is very difficult to observe unless the animals are being extensively observed, so not a good marker of reproductive activation.
The statement on lines 346-347 and content on Figure 5 may need rewording in the light of comments above and to follow.
17. The study needs further nuancing to fully address the relative the effects of queen behavior and odour signalling and a crucial experiment remains to be done. While animals have been separated and kept in odour contact, and in one colony the queen was removed but isopropyl myristate added to maintain suppression, anosmic NBrs (methimazole ablated) should have been investigated (with appropriate profiling) while remaining as subordinates within their colonies in a similar way to the marmoset study of Barrett et al. (1993) – where anosmic subordinate females stayed suppressed because behavioral and other cues over-rode the effect of odour. Only by doing this can the effects of dominance and queen odour be teased apart.

References

Barrett J, Abbott DH, George LM. (1993) Sensory cues and the suppression of reproduction in subordinate female marmoset monkeys, Callithrix jacchus. J Reprod Fertil. 97(1):301-10. doi: 10.1530/jrf.0.0970301. PMID: 8464022.
Bennett, N.C.; Faulkes, C.G.; Voigt, C. (2022) Socially Induced Infertility in Naked and Damaraland Mole-Rats: A Tale of Two Mechanisms of Social Suppression. Animals 2022, 12, 3039. https://doi.org/10.3390/ani12213039
Clarke F. M. and Faulkes C. G. (1999) Kin discrimination and female mate choice in the naked mole-rat Heterocephalus glaber. Proc. R. Soc. Lond. B.2661995–2002.
Crespi B.J., Yanega D. (1995) The definition of eusociality. Behavioral Ecology 6:109-115
Doty, R. L. (2010). The Great Pheromone Myth. Johns Hopkins University Press.
Gilbert J.D., Rossiter S.J., Faulkes C.G. (2020) The relationship between individual phenotype and the division of labour in naked mole-rats: it’s complicated. PeerJ 8:e9891 https://doi.org/10.7717/peerj.9891
Holmes, M.M., Goldman, B.D. (2021). Social Behavior in Naked Mole-Rats: Individual Differences in Phenotype and Proximate Mechanisms of Mammalian Eusociality. In: Buffenstein, R., Park, T.J., Holmes, M.M. (eds) The Extraordinary Biology of the Naked Mole-Rat. Advances in Experimental Medicine and Biology, vol 1319. Springer, Cham.
Janse van Vuuren, A.K., Süess, T., Finn, K., Hagenah, N., Ganswindt, A., Hart, D.W., & Bennett, N.C. (2025). Hormonal lockdown: How mole-rat societies enforce infertility in helpers. Hormones and Behavior, 147, 105836.
Mooney S.J., Filice D.C.S., Douglas N.R., & Holmes M.M. (2015) Task specialization and task switching in eusocial mammals. Animal Behaviour 109:227-233
Seney, M. L., Kelly, D. A., Goldman, B. D., Sumbera, R., and Forger, N. G. (2009). Social structure predicts genital morphology in African mole-rats. PLoS One 4:e7477. doi: 10.1371/journal.pone.0007477
Wyatt, T. D. (2014). Pheromones and animal behavior: Chemical signals and signatures (2nd ed.). Cambridge University Press.

Referee #3

(Remarks to the Author)
Khallaf et al from the Lewin lab report the identification of a compound released by a female naked mole-rat, the queen “odor”, that suppresses reproduction in other females in the same colony. This is a spectacular finding, which solves a long-standing mystery of how a single female is able to dominate and control reproduction in this eusocial mammal. The authors first show that naked mole-rats use chemical cues presumably detected by the olfactory epithelium to recognize other individuals, and that aggression between individuals is reduced when the olfactory epithelium is ablated with methimazole, which effectively ablates the olfactory epithelium. They next analyze compounds released by naked mole-rats, and show that Isopropyl myristate is selectively released by queens, and release is higher during ovulation. Most remarkably, they show that isopropyl myristate is sufficient to maintain females in a non-reproductive status. When the queen is removed and isopropyl myristate is introduced, the remaining females stay in a non-reproductive status (smaller, high prolactin levels).

Upon removal of isopropyl myristate, prolactin levels in all females drops, the females begin to fight and a single female ascends. They also show that isopropyl myristate is sufficient to prevent females removed from the colony from becoming pregnant when placed with a male naked mole-rat. Overall, notwithstanding some concerns raised below, the authors provide convincing data that they have identified the mechanism that supports reproductive suppression in this eusocial mammal. The manuscript is well written and will be of great interest to a general scientific readership.

Concerns:
1. The authors did not in my view adequately address the question of whether the isopropyl myristate could be detected by the vomeronasal rather than olfactory system. Although the author cite evidence that the VNO is likely to be non-functional, based on its smaller size relative to other rodents, this does not rule it out as a possible site of sensory detection. One way to answer this question would be to look at whether methimazole causes cell death in the VNO, or if it spared. If spared, then the evidence would point clearly to detection by the olfactory epithelium. If ablated, then I believe the jury is out as to where the compound is detected.

Similarly, the evidence that the olfactory systems is specifically activated by isopropyl myristate (Fig 3) is not entirely convincing. In fig 3e, the response to Isopropyl myristate appears to encompass much of the olfactory bulb. Extended fig 4 e shows some evidence of higher labeling of some glomeruli, but it is hard to tell if that is just normal variation in staining. As this experiment was presumably repeated multiple times, I suggest the authors examine and show the cFos staining from all animals studied. This would allow them to address whether the activated glomeruli show a stereotyped localization, as expected if the compound is truly acting on a single or small number of odorant receptors.
If the authors cannot provide evidence to distinguish between detection by the olfactory epithelium and the VNO, they should state this. This would in no way detract from the main conclusion of the paper, showing a clear role for a chemical cue, isopropyl myristate, in the maintenance of reproductive suppression in naked mole-rats
Minor:
Fig 3a. The cartoon looks like the recording was made in vivo but the text says ex vivo
Fig 3b. What is the thick black line – is this the average response? What does the DMSO control look like?
Fig 4e. This result is not well described. Are these females that were removed from the colony (from Fig 4d), who were treated with methimazole? Or just some of the females in the colony. Showing a timeline of the experiment would help.

Version 1:

Reviewer comments:

Referee #1

(Remarks to the Author)
The authors have done a comprehensive and impressive revision of the paper, have addressed all of my prior concerns, and I am enthusiastic about the work. The fUSI is remarkable and very informative, and I appreciate the revised EOG data. I defer to the authors on the other minor recommendations that they chose to not pursue.

Review prepared by Leslie Vosshall

Referee #2

(Remarks to the Author)
The manuscript is substantially improved and stronger than the original submission. In particular, the expanded comparative analysis across multiple Fukomys species resolves issues with the evolutionary framing. The addition of longitudinal fecal steroid profiling strengthens the interpretation of reproductive activation and suppression (although this is only provided for n=3 pairs), and the functional ultrasound imaging is an interesting inclusion. These additions address several of my primary concerns from the first round of reviewing. However, despite these improvements, the manuscript still overstates the exclusivity and mechanistic sufficiency of isopropyl myristate in a number of places. For a study of this conceptual impact particularly in a journal such as Nature, the strength of the claims must match the strength of the evidence in the absence of the key within colony anosmia experiment that I suggested.
Remaining issues that require revision are detailed below:

1. Overstatement of exclusivity of isopropyl myristate and mechanism
The manuscript repeatedly uses language implying that isopropyl myristate governs, enforces, or orchestrates reproductive suppression in naked mole-rats.
For example:
The Abstract states that the study reveals “a single chemical cue governing reproductive suppression”.
The Discussion refers to “a solitary pheromone that can enforce reproductive hierarchies, prevent conflict, and sustain eusociality.”
The Results state that isopropyl myristate “alone was sufficient” to preserve reproductive suppression.
Other prominent compounds in the queen odour profile were not tested, so there could also be further compounds that reflect the presence of a breeding queen.
The statements above imply that isopropyl myristate alone is both necessary and sufficient for reproductive regulation in intact colonies. However, the key experiment disentangling olfactory cues from behavioural dominance interactions within intact colonies was not performed, the reason being a lack of ethical permission – this remains a major weakness of the

study, together with only n=1 for the queen removal experiment. So, while the present data demonstrate that isopropyl myristate is a queen-associated chemosignal capable of maintaining endocrine suppression and colony stability under defined experimental manipulations, it is not possible to establish that it operates independently of other social modalities in intact colonies (or indeed other queen specific compounds). Given that the key experiment (within-colony anosmia) cannot be done, then the causal language must be moderated throughout to reflect this. Therefore, I recommend replacing "governing reproductive suppression" with language such as "capable of mediating reproductive suppression", removing or modifying statements implying that a single pheromone "enforces eusociality" and adding an explicit limitation paragraph acknowledging that reproductive suppression in naked mole-rats is likely multimodal, with behavioural and chemosensory components operating in parallel. This is not a semantic issue, it is a matter of aligning interpretation with evidence. In terms of explaining the formation of two-queen colonies and breeder males recruitment some brief mention of this should be included in the discussion, e.g. from lines 459. In the rebuttal the authors suggest that it occurs when a reigning queen becomes reproductively impaired but this is not always so. Two queens (or even three) can co-exist and breed in asynchrony, and breeding queens can also be deposed from within by a non-breeding sister or daughter reproductively activating. There have been rare cases of dual queens also reported in the wild (see Braude's work) so it can also occur in natural colonies too. Granted, these cases are not common, by all point to changes in dominance behaviour causing release from suppression in the presence of the queen odour and cannot be ignored.

2. Behavioural versus olfactory contributions

The present study demonstrates a powerful chemosensory component but does not eliminate other mechanisms (or indeed other compounds). Although practical/ethical constraints prevented the within-colony anosmia experiments, this limitation must be more explicitly acknowledged. The current Discussion briefly notes that vomeronasal contributions cannot be excluded (although I agree that the evidence for the primary olfactory epithelium is good), but it does not adequately address the broader issue of multimodal suppression. A clear statement is needed acknowledging that behavioural dominance cues may contribute independently or synergistically.

3. Further clarification of endocrine and reproductive measures

The addition of fecal progesterone and testosterone metabolite profiling, although cruder than urinary hormone sampling is an improvement and strengthens the manuscript. However, the main text does not sufficiently clarify the lag time between circulating hormone changes and fecal metabolite detection. In the methods, n=6 pairs were used in the separation studies, yet only n=3 are included in the hormone assays (again a low n). This need to be explained. Were these part of the original study or extra experiments? If the former then the other three colonies we still don't know if lack of pregnancy in the scent transfer colonies wasn't due to deficiencies in the males. In Extended Fig. 6c, it is stated in the legend "Testosterone metabolites are significantly elevated in the blank condition compared with colony bedding and isopropyl myristate". Looking at the figure and the error bars it looks like all three groups are different from one another. Was a post-hoc test done to test for significance among all three groups? As written it implies that the blank is different from the other groups, which are not sig. diff. between each other. Is this so? If not, this needs discussion.

Lines 874-879: "Ovulation was inferred retrospectively using established reproductive staging in naked mole-rats, based on longitudinal behavioural observations, and defined reproductive states (pre-mating, mating, pregnancy, and lactation)", and on lines 882-884 "Breeding females of the four Fukomys spp. were sampled across two reproductive stages (ovulation and pregnancy), as were naked mole-rats. The three Cryptomys spp. females were likewise sampled during ovulation and pregnancy". Given that gestation in the naked mole-rat has been reported to be from 66-74 days, and the follicular phase is on average 6 days, counting back from birth to estimate ovulation could potentially entirely miss the entire follicular phase, let alone the small time window of ovulation within the follicular phase – so how confident can the authors be with their estimates of "ovulation" etc? Were any matings observed?

4. Copulatory plug terminology

The manuscript continues to use the term "plug formation". Even with contextual clarification, this phrasing risks anatomical inaccuracy, as naked mole-rats do not form canonical rodent copulatory plugs. I recommend replacing this terminology throughout with neutral descriptive language or removing it entirely, particularly now that endocrine data provide a more robust endpoint.

Minor comments/corrections

Abstract: "These findings reveal a single chemical cue governing reproductive suppression in a mammal, linking insect and mammalian eusociality" This statement has also been indicated above as needing toning down – but also in the best characterised insect system (the honey bee) the suppressing chemosignal emitted by the queen is composed of multiple (specific) compounds, not one.

Line 46: "This compound is detected by olfaction, alters prolactin and progesterone levels in non-breeders," - progesterone is only altered in non-breeder females.

Line 134: isopropyl myristate wasn't found in urine – this is interesting as urine is the most common source of rodent chemosignals. Maybe emphasise?

Lines 241-242: state whether primary or accessory olfactory bulb (I assume primary, but be clear as this is important).

Line 458: damaransis is incorrectly spelt.

Referee #3

(Remarks to the Author)

This remains an excellent paper that reports a very important finding, that isopropyl myristate is released by queens and suppresses subordinate reproduction in this eusocial mammal. I therefore, in principle, support publication in Nature.

However, I do not believe the data presented allow the investigators to claim a role for the main olfactory system, rather than

the accessory olfactory systems, in mediating effects of isopropyl myristate, not withstanding the diminished size of the VNO in the NMR. Some issues with the data presented related to cFos imaging include: (1) duplication of images in extended data 4e (compare propidium iodide with cFos), (2) uncertainty regarding identification of the AOB (extended data 4e, red circle; different in top and bottom images, and no markers used to identify it) (3) large scatter of data and n=3 on measurement of cFos in AOB (Fig 3) which preclude any conclusion regarding statistical significance, (4) concern that methimazole treatment may damage the VNO, or even respiratory epithelium, absent any data indicating otherwise, (5) cFos data (Fig 3 and Extended data Fig 4) remains unconvincing, absence replicates and clear cFos activity in a subset of mitral/tufted cells or glomeruli; supplementary movie is useless as it shows widespread cFos and no control.

Regarding the new data obtained using fUSI imaging, it would be necessary to perform control experiments to confirm that areas identified indeed correspond to olfactory areas. Moreover, unilateral responses need some explanation, and replicate responses (same animal) should be shown.

Thus, for these reasons and others, I believe that the data from cFos IHC and fUSI, as currently presented, could be misinterpreted and the paper will be stronger if it is not included. Similarly, the text should be revised to keep open all possibilities (there are also olfactory subsystems that need to be considered). A deeper dive into the mechanisms by which Isopropyl myristate regulates behavior in this fascinating species certainly merits further investigation.

Version 2:

Reviewer comments:

Referee #2

(Remarks to the Author)
The manuscript is substantially improved and several of my previous technical concerns have been addressed. In particular, the authors have moderated aspects of the language, clarified elements of the endocrine analyses, and removed problematic terminology. The addition of a statement acknowledging that behavioural and chemosensory cues may act in concert is also welcome (and important). However, I do not consider my main interpretive concern to be fully resolved.

The issue was not solely one of wording, but of ensuring that the strength of the claims is consistently aligned with the strength of the evidence. While the Abstract now uses more appropriate phrasing (e.g. “can mediate”), stronger assertions remain in several key sections of the manuscript. For example, the Introduction and Discussion still describe isopropyl myristate as a “single” or “solitary” compound capable of coordinating colony-wide reproductive suppression and sustaining eusociality. Similarly, the Results continue to frame the compound as sufficient to preserve reproductive suppression at the colony level. The issue of dual queen colonies is avoided “given the word limits of the manuscript” and should be mentioned at least briefly as an unresolved issue requiring future consideration. Taken together, these statements still imply a level of mechanistic sufficiency and generality that is not fully supported by the data. The study convincingly demonstrates that isopropyl myristate is a queen-associated chemosignal capable of mediating endocrine suppression and stabilizing social interactions **under defined experimental conditions**. However, very importantly, it does not establish that this compound alone governs reproductive suppression **in intact colonies independently of other queen-associated compounds or behavioural dominance interactions**. This distinction remains crucially important, particularly given that the within-colony anosmia experiment was not performed and that the queen-removal paradigm is based only on a **single colony**.

Although the Discussion now includes a sentence acknowledging that behavioural and chemosensory cues may act in a coordinated manner, this point is not fully integrated into the overall framing of the study. I still think the manuscript would benefit from more consistent and explicit alignment of the central claims with this limitation throughout, particularly in the Introduction and Discussion. One example in the introduction it is stated “a single, naturally occurring chemical capable of orchestrating colony-wide reproductive suppression and social cohesion” is still very strongly worded in terms of exclusivity when any behavioural component from the queen has not been tested. In the discussion: “a single compound can coordinate physiology and behaviour across the colony” and “a solitary pheromone that can enforce reproductive hierarchies, prevent conflict, and sustain eusociality in mammals” are problematic statements and the language asked to be removed. The title itself (which many people may not read much beyond) “A queen odour mediates reproductive suppression in a eusocial mammal” gives the impression that just a queen odour exclusively causes suppression in all naked mole-rat colonies and I would recommend rewording in some way.

I therefore consider the manuscript to be improved, but recommend further revision to ensure that the conceptual framing is fully consistent with the evidence. Without this the results are in danger of misinterpretation by the casual reader and given that NMRs are of broad and topical interest misinformation is likely to spread.

Version 3:

Reviewer comments:

Referee #2

(Remarks to the Author)

The manuscript has been improved through further revision. In particular, the authors have now moderated the strongest claims throughout the manuscript, clarified that the observed effects occur under defined experimental conditions, and explicitly acknowledge that behavioural and additional sensory mechanisms may contribute to reproductive suppression in intact colonies. The inclusion of discussion regarding dual-breeder colonies is also welcomed. The only place where I still think they are stretching slightly is the final sentence: “a queen pheromone that can regulate reproductive hierarchies, prevent conflict, and sustain eusociality in mammals” This is still somewhat grand and broad, but it is much less problematic than: “a solitary pheromone that can enforce…”. At this point, I personally would not hold the paper over that sentence alone. So, while some broad framing remains, I now consider the conceptual interpretation to be sufficiently aligned with the evidence presented.

Open Access This Peer Review File is licensed under a Creative Commons Attribution 4.0 International License, which permits use, sharing, adaptation, distribution and reproduction in any medium or format, as long as you give appropriate credit to the original author(s) and the source, provide a link to the Creative Commons license, and indicate if changes were made.
In cases where reviewers are anonymous, credit should be given to 'Anonymous Referee' and the source.
The images or other third party material in this Peer Review File are included in the article’s Creative Commons license, unless indicated otherwise in a credit line to the material. If material is not included in the article’s Creative Commons license and your intended use is not permitted by statutory regulation or exceeds the permitted use, you will need to obtain permission directly from the copyright holder.
To view a copy of this license, visit https://creativecommons.org/licenses/by/4.0/

Nature 2025-09-24315A: RESPONSE TO REVIEWERS

We are grateful to the reviewers for their encouraging, and insightful evaluations of our manuscript, "A queen odour mediates reproductive suppression in a eusocial mammal." We were delighted that the referees found the study to be a *"fascinating," "compelling,"* and *"spectacular"* solution to a long-standing question in the field of naked mole-rat biology. In particular, the reviewers highlighted the conceptual significance of identifying a single, queen-derived chemical signal that enforces reproductive suppression, noting that the work *"solves a long-standing problem," "links themes from eusocial insects to mammals,"* and provides *"convincing data"* for a chemical mechanism underlying colony-wide social regulation.

The detailed and constructive critiques have been invaluable, and we believe they have substantially strengthened the clarity, rigor, and impact of the revised manuscript. We summarise first briefly the main new pieces of data, before providing detailed responses to each of the reviewers' specific issues:

- **Additional aggression control assay**: Mixed-treatment pairings show that methimazole-treated high-ranking animals retain aggressive capacity when challenged, demonstrating that olfactory ablation selectively disrupts aggression initiation rather than causing general lethargy.
- **Expanded chemical characterization**: We added a comprehensive list of all identified compounds detected in naked mole-rat odour profiles (new Supplementary Table 1).
- **Expanded comparative species analysis**: Inclusion of three additional *Fukomys* species and reanalysis across all species reveal that all *Fukomys species* seem to make appreciable levels of isopropyl myristate, this data is consistent with strong reproductive skew in this genus.
- **Expanded olfactory controls**: New EOG analyses show that air and DMSO elicit indistinguishable responses, whereas isopropyl myristate evokes significantly larger signals, addressing concerns about solvent effects.
- **Expanded c-Fos dataset with additional brains and new accessory olfactory bulb analysis:** Analysis of the AOB in animals exposed to isopropyl myristate demonstrated no increase in the number of cfos cells, morphological measurements confirmed that the AOB is considerably smaller in naked mole-rats compared to mice.
- **New fUSI experiments**: Functional ultrasound imaging directly comparing air, DMSO, and isopropyl myristate reveals robust, solvent-independent activation of olfactory circuits, strengthening evidence for olfactory specificity.
- **Male prolactin and weight measurements added:** We now include male prolactin and weight measurements following queen removal and isopropyl myristate withdrawal (**new Extended Data Fig. 6g**).
- **New reproductive hormone data**: Longitudinal measurements of fecal progesterone metabolites (fPMs) in females and fecal testosterone metabolites (fAMs) in males show that isopropyl myristate and colony bedding suppress reproductive endocrine activation,

complementing pregnancy outcomes and strengthening the mechanistic link to reproductive suppression.

Referee #1 (Remarks to the Author):

Khallaf and co-authors describe a single queen-derived molecule, isopropyl myristate, that has the power to control reproductive behavior in two species of eusocial naked mole rats. This is a fascinating and compelling story with many points of strength. It solves a long-standing problem in the field, and links themes from eusocial insects to this new discovery in eusocial mammals. The authors show that social interactions require the olfactory system (Figure 1), identify a single molecule (isopropyl myristate) that is specific to queens and specific to reproductive stage (Figure 2), that the molecule activates the olfactory system (Figure 3 – though see my serious concern below), that isolated non-breeder females begin to produce this molecule, and that the molecule or colony bedding will inhibit mating (Figure 4), and finally that in a real colony situation that the molecule will enforce a phase of peaceful behavior after queen removal and that its withdrawal triggers conflict and the eventual emergence of a new queen (Figure 4).

RESPONSE: We thank the reviewer for this positive and thoughtful evaluation. We are very encouraged by your assessment that our findings resolve a long-standing problem and provide a compelling link between eusocial insects and mammals.

There are a few points that the authors should address to further strengthen the work:

1. Figure 1 is not a strong start to the paper and doesn't really address the core question of the queen odor – it's just a demonstration that olfaction matters for aggression behavior. I would consider reorganizing the narrative to talk about the search for the queen substance by opening with Figure 2, which is very strong.

RESPONSE: Thanks for the suggestion. While we agree that Figure 2 presents a strong and exciting entry point into the identification of the queen-specific compound, we deliberately begin with Figure 1 because the role of olfaction in naked mole-rat social regulation has been largely overlooked for decades. Establishing that olfaction is causally required for core social behaviors provides a necessary conceptual foundation for the subsequent search for a queen-derived chemical signal. By first demonstrating that disruption of olfactory input fundamentally alters social recognition, we aim to motivate and justify the chemical analyses that follow, rather than presenting the identification of isopropyl myristate in isolation. In this figure, we also introduce the methimazole treatment that we later use to directly link olfactory function to hormonal status, showing that non-breeding animals that lost the ability to smell exhibit a marked reduction in circulating prolactin levels (revised Fig. 4e).

2. Figure 3 is a weak demonstration that isopropyl myristate acts through the olfactory system and is the weakest point in an otherwise fantastic paper. This is probably no fault of the authors. Isopropyl myristate is extremely non-volatile and it's not clear that the response in the EOG (Figure 3a-c) is materially different than the response to DMSO. The fact that the authors do not show the EOG response to DMSO is telling. The data points in Figure 3c show that for most experiments, isopropyl myristate is not different than DMSO. For the c-fos experiment, the authors use clean air rather than DMSO as the control. Therefore, there is no way to disambiguate whether the (weak) c-fos signal is due to DMSO, or isopropyl myristate, or a combination. My recommendation is NOT to ask the authors to redo these difficult experiments, but simply remove these data and these claims from the paper entirely. It's possible that some different method of applying the odors or another concentration would show olfactory responses, but it does not seem worth sacrificing more precious animals and more

time of the scientists involved to do this experiment. By common sense readers will believe that this molecule is likely acting on the olfactory system, and the very compelling data in Figure 4 are persuasive that it is a molecule that has the power to disrupt endocrinology, mating, colony structure, etc. This is enough at this stage for this story!

RESPONSE: Thank you for this careful critique and we do share the concern about distinguishing solvent effects from odor-evoked activity. To address this, we have revised the manuscript in two ways:

- We expanded our EOG analyses (**new Extended Data Fig. 4c**) to explicitly compare responses to air and DMSO, which do not differ from one another, consistent with previous literature showing that EOG responses can be triggered by the mechanical force of airflow alone (e.g. Grosmaitre et al 2007 PMID 17310245). In contrast, isopropyl myristate elicits significantly larger EOG peak amplitudes than either air or DMSO. We now also explicitly show the EOG response to DMSO in the revised figure (**new Extended Data Fig. 4b**).

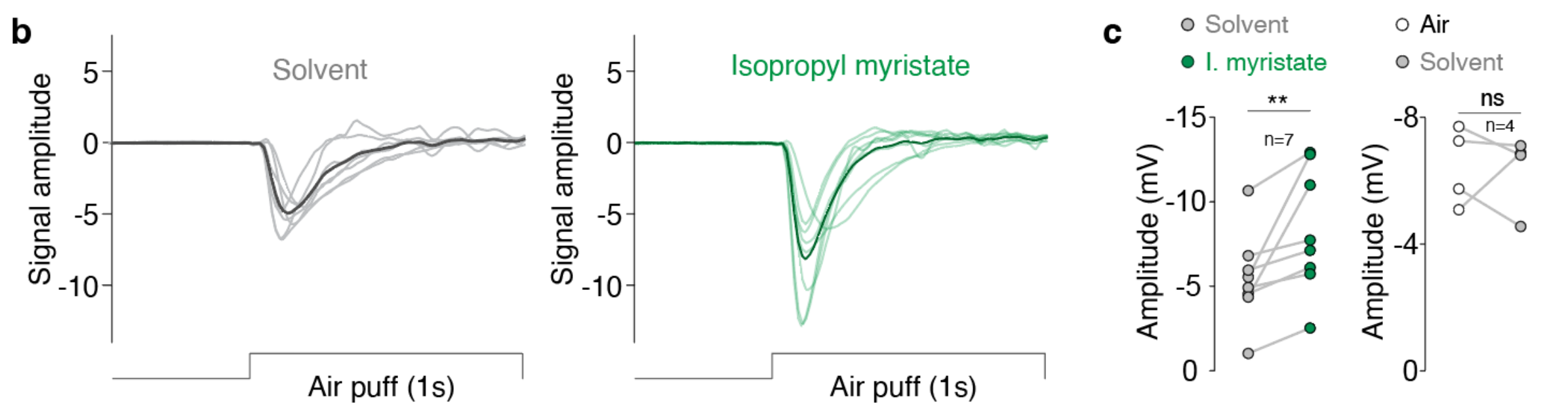

Extended Data Fig. 4b, Representative EOG traces in response to a 1-second air puff of solvent (DMSO) and isopropyl myristate diluted 1:10 (v/v) in DMSO.
c, Quantification of EOG peak amplitude (left) and area under the peak (right) shows significantly greater responses to isopropyl myristate compared to solvent, while air and solvent controls do not differ. Each dot represents one animal; paired *t*-test; ns $p > 0.05$, $^{**}p < 0.01$.

- Second, and most importantly, we added a new functional ultrasound imaging (fUSI) dataset that provides an independent and more sensitive measure of olfactory circuit activation (**new Fig. 3d-g** and **new Extended Data Fig. 5a**). fUSI reveals robust, stimulus-locked increases in cerebral blood flow within olfactory cortical regions in response to isopropyl myristate that are significantly greater than both air and DMSO controls (**new Fig. 3e**). Notably, air and DMSO do not differ in this assay, reinforcing the specificity of the isopropyl myristate response.

This represents, to our knowledge, the first application of fUSI in naked mole-rats. We emphasize its particular suitability for this species: fUSI is minimally invasive, compatible with repeated measurements, and does not require genetic tools or terminal procedures, making it especially well-suited for long-lived, socially housed animals. Indeed, we were able to make fUSI measurements up to 2-5 times from the same animal over multiple experimental days. Strikingly, acute brain activation in response to isopropyl myristate was seen consistently in the same areas across sessions from single animals. The robustness of fUSI signals can be become strongly attenuated when the cranial bones are thicker impeding ultrasound penetration and for this reason we concentrated on cortical areas in these experiments as the olfactory bulb is buried beneath thicker cranial structures that impede imaging. For the same reason we were only able to make good quality imaging from smaller naked mole-rats with thinner bone structure. Indeed, our attempts to obtain good quality fUSI data from the much larger queens

was unsuccessful for this reason. The convergence of electrophysiology, immediate-early gene expression, and fUSI thus provides complementary evidence that isopropyl myristate activates olfactory circuits, while the colony-level behavioral and endocrine effects remain the central strength of the study.

We have revised the Results, Methods, and figure organization accordingly and believe these additions substantially strengthen the manuscript while directly addressing the reviewer's concerns.

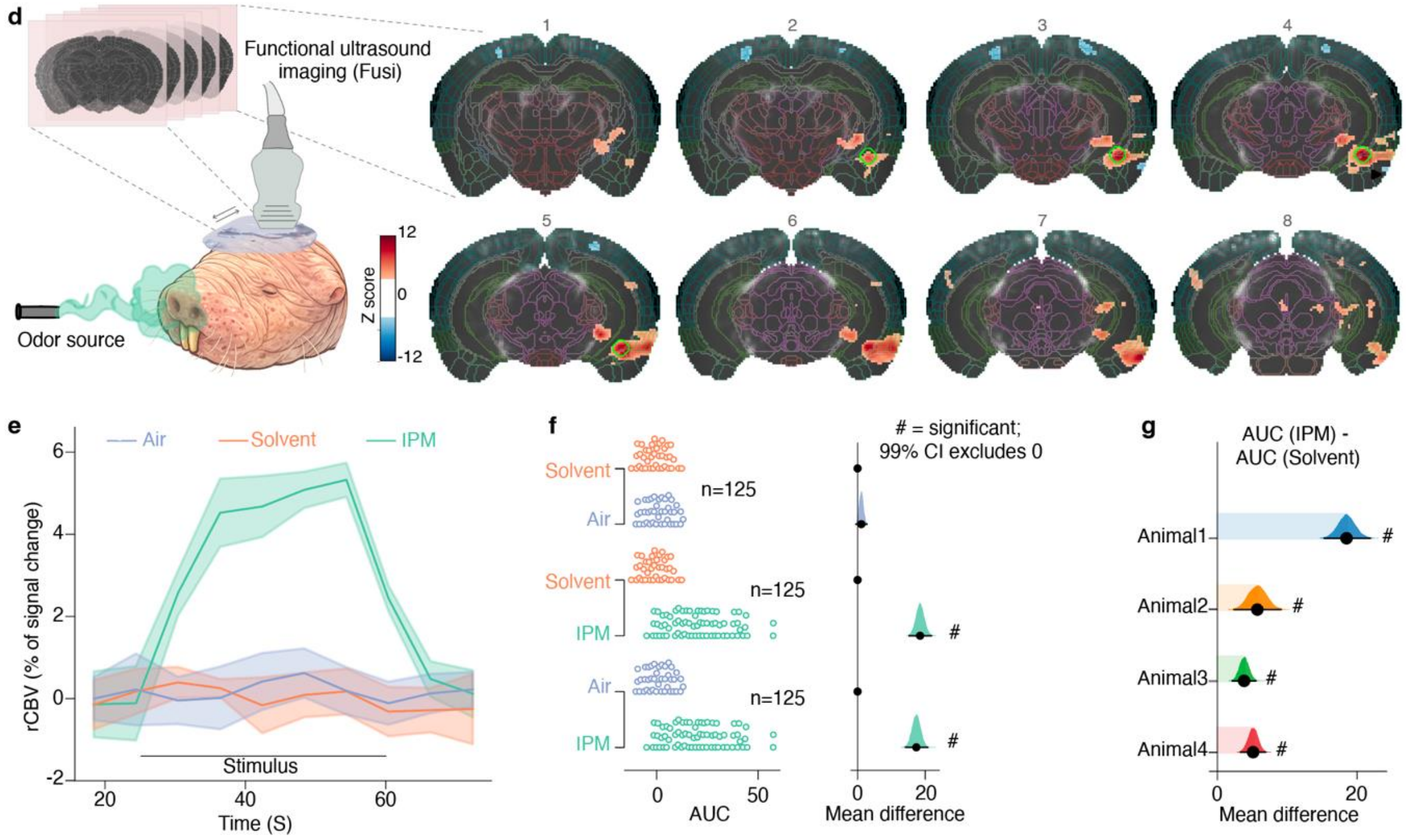

Fig. 3d, Functional ultrasound imaging (fUSI) during olfactory stimulation in anesthetized animals. Left, schematic of fUSI setup. Right, coronal brain sections showing odour-evoked activity (Z-score) by isopropyl myristate in one animal (animal#1). Representative coronal planes (1–8, anterior to posterior) show anatomical overlays from the Allen Mouse Brain Atlas on grayscale power Doppler images, with Z-scores indicated in color. Statistical thresholds (±3.58; $\alpha = 0.01$, FDR-corrected) were applied, and clusters <30 voxels were discarded. A green sphere ($r \approx 400$ µm) marks the voxel of maximal response. Significant hyperaemia was observed in olfactory-related regions, including the piriform cortex, geniculate nuclei, and amygdala-associated areas.
e, Relative cerebral blood volume (rCBV; % signal change) within the green sphere for air, solvent (DMSO), and isopropyl myristate stimulation. Traces show mean ± s.d. across imaging sessions, averaged over the stimulus period.
f, Area under the curve (AUC) of rCBV responses for individual voxels (n = 125) within the green sphere across conditions. Right, mean AUC differences with 99% confidence intervals from permutation testing. Isopropyl myristate induces a significant rCBV increase compared with air and solvent, whereas air and solvent do not differ.
g, Effect sizes and 99% confidence intervals for DMSO versus isopropyl myristate across four animals (including data from multiple imaging sessions per animal). Only this comparison is shown as not all animals received air stimulation. Hashtags indicate confidence intervals excluding zero.

Minor points

Line 9-10 – this seems speculative as a reason for high-ranking animals to avoid the odorant

RESPONSE: We have now revised the text to ("*elicits avoidance in high-ranking animals*") lines (7-8), removing any inference about the underlying motivation for this behavior.

Line 70 – just curious if the methimazole injection itself causes lethargy that might be contributing to the reduced aggression behavior? Or were there control experiments to establish that the effect is specific to ablating the olfactory system? I see that Extended Data Fig 1 has some good countering points that would be good to call out in the Results for Figure 1

RESPONSE: Social rank remained stable following methimazole treatment (Extended Data Fig. 1c), indicating that activity and general dominance-related behaviours were preserved. However, to further directly test whether methimazole suppresses aggressive behaviour per se, we now include an additional experiment in which two unfamiliar animals were paired, with one individual saline-injected and the other methimazole-injected. In these encounters, saline-treated animals reliably initiated aggression, while methimazole-treated animals actively fought back, demonstrating preserved aggressive capacity. Quantification of the aggression index confirmed robust aggression (**new Extended Data Fig.1d**), and representative behaviour is shown in **new Supplementary Video 6**. We have revised the Results and figure organization accordingly and added the following in lines (109-113) "*Importantly, in mixed-treatment encounters, methimazole-treated high-ranking animals actively fought back when challenged by saline-treated opponents, demonstrating that olfactory ablation selectively disrupts the initiation of aggression, rather than aggressive behaviour per se (Extended Data Fig. 1d and Supplementary Video 6)..*"

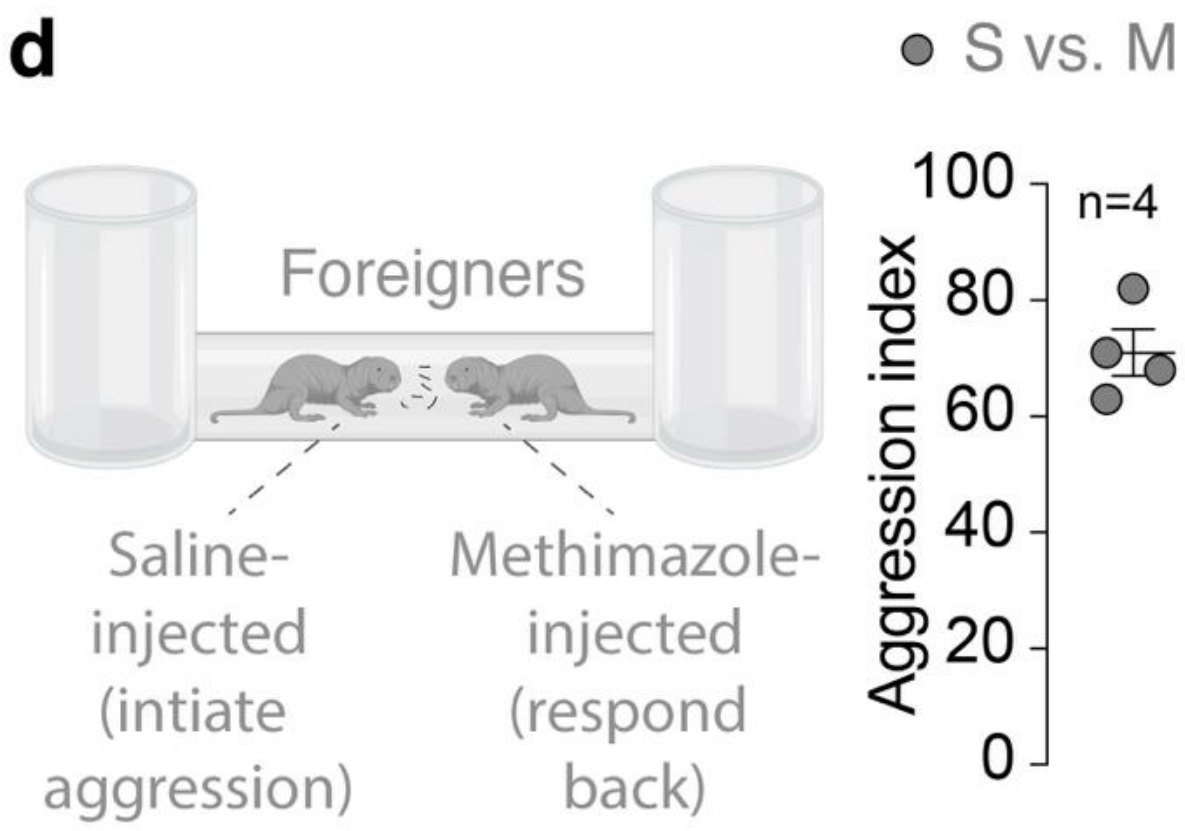

Extended Data Fig. 1d, Methimazole does not suppress aggressive behaviour. Schematic showing saline-injected animals initiate aggression, while methimazole-injected animals respond by fighting back. Aggression index quantification (right; n = 4) shows preserved aggression in methimazole-treated animals (see Supplementary Video 6).

Line 86 – what is the food odor? The methods do not provide adequate information (Line 690-691)

RESPONSE: The food odour has now been explicitly defined in the Methods as ProNutro, a cereal-based dietary supplement (line 865).

Line 90, Line 97-107 – is this an aggression assay where both are either saline or methimazole treated, or are you comparing the aggressiveness of a saline vs. methimazole animal? In Figure 1d, are these animals of the same or different ranks? The issue of social rank is not well explained in the behavioral assays. This is not clear from the figure or text, and as it's a key experiment it is necessary to be very clear about what the experimental design is.

RESPONSE: We have now revised the Results text (lines 101-109) and Fig. 1d legend and labels to explicitly state that aggression assays were performed by pairing two unfamiliar animals from different colonies that were matched for both treatment and social rank. Specifically, Fig. 1d shows pairings of high-ranking individuals, with both animals receiving the same treatment (saline–saline or methimazole–methimazole). We further clarify that low-ranking animals show minimal aggression regardless of treatment, as shown in Extended Data Fig. 1b. In addition, we now explicitly describe the **new Extended Data Fig.1d** (the mixed-treatment control experiments) demonstrating that methimazole-treated animals retain aggressive capacity when challenged. Together, these clarifications make the experimental design and interpretation of Fig. 1d explicit in both the text and figure legend.

Line 117 (Figure 2e-g) – the use of bars and stars to indicate statistical differences is not clear, I suggest the alternate approach of labeling each set of data with a different letter if they are statistically different.

RESPONSE: We thank the reviewer for this suggestion. After consultation with colleagues outside the author group, we retained the asterisk-based notation, as it is widely used and clearly conveys the degree of statistical significance; the statistical comparisons and thresholds are now explicitly defined in the figure legends.

Line 140-142, Figure 2h, Lines 174-178, Lines 396-397 – this experiment seems like a non-sequitur in this otherwise highly compelling and persuasive figure. While it is convincing that the queens explore the maze more than subordinates, it is an overinterpretation to conclude that this is because she is scent marking. There could be other neural/behavioral differences between queens and subordinates that are driving the differences in behavior, independent of their ability to produce isopropyl myristate. Also, it was not clear if the authors measured whether isopropyl myristate was physically deposited by the queen, so the statement (Line 396-397) that she is doing 'tunnel rubbing' is not substantiated by data.

RESPONSE: We agree that the original interpretation risked over-attributing increased maze exploration by queens to scent marking. We have therefore revised the text to remove speculative language linking maze exploration to scent marking or tunnel rubbing. The result is now described in lines (185-187) "*Queens explored a significantly larger proportion of the maze than non-breeders (Fig. 2h), in line with recent quantitative findings that breeders more actively patrol the colony space compared to non-breeders*". We additionally cite recent work (Yamakawa et al 2025 PMID 41061078) consistent with our findings showing that queens patrol a significantly larger proportion of a novel maze than subordinates.

Line 161-170, Line 904 (Extended Data Fig 3e) – these are important data that should be moved to the main Figure 2, which has plenty of space for these exciting data. This result greatly strengthens the case that isopropyl myristate is the molecule of eusociality.

RESPONSE: We thank the reviewer for highlighting the importance of these comparative data. In response, we (i) broadened our sampling to include three additional *Fukomys* species, (ii) re-ran comprehensive GC–MS presence/absence and abundance analyses across all sampled species, and (iii) mapped the occurrence of isopropyl myristate onto the phylogeny. These new data are now presented in **new Fig. 3i,j,** and the corresponding text has been added to lines 185-190 of the revised manuscript.

Line 179 (Figure 3b) – would the authors please provide example traces of responses to

solvent? The differences between DMSO and isopropyl myristate are not extremely compelling in Figure 3c

RESPONSE: We now show the EOG response to DMSO in the **new Extended Data Fig. 4b**.

Line 179 (Figure 3d-e), Line 211-215 – given that the EOG responses of DMSO and isopropyl myristate are not extremely different, it is problematic that the controls for the c-fos experiment were simply air-exposed rather than DMSO-exposed. It is therefore not possible to interpret how much of the c-fos immunoreactive cells are specifically activated by isopropyl myristate vs the DMSO solvent.

RESPONSE: We agree that, in the original version, the use of air alone as a control in the c-Fos experiment limited the ability to disentangle responses to isopropyl myristate from potential effects of the DMSO solvent. As stated above, we now include new data showing that EOG responses to air and DMSO do not differ, consistent with previous reports that EOG signals can be elicited by the mechanical force of air movement. In addition, we complemented the c-Fos analysis with functional ultrasound imaging, which enabled direct comparison of responses to air, DMSO, and isopropyl myristate within the same animals.

Line 216-226 – it's impressive that the authors went looking for a human isopropyl myristate receptor, but I do not think that the negative result is particularly interpretable. It is possible that such a receptor exists, but it does not show functional expression in the assay being used. To avoid disrupting the flow of the paper with this interesting non-sequitur, I would recommend moving all of this text to the methods and out of the main paper (but retaining it as it's an interesting set of negative data for experts in the field).

RESPONSE: We agree that the negative results from the human olfactory receptor screen should be interpreted with caution. However, we chose to retain this analysis in the main text because it provides important context regarding the presence of isopropyl myristate in another mammalian species, humans. To minimize disruption to the narrative flow, we have trimmed the text and revised it to present the findings descriptively, with explicit acknowledgment of the assay's limitations.

Line 241 (Figure 4c) – as mentioned above, it would be best to indicate significance using the notation of different letters indicating significance rather than the many bars and stars.

RESPONSE: We retained the asterisk-based notation for clarity and consistency, with all statistical thresholds defined in the figure legend.

Review prepared by Leslie Vosshall

Referee #2 (Remarks to the Author):

This interesting study investigates the potential role of a queen derived chemosignal in reproductive suppression in naked mole-rats (NMRs), in the form of a single compound (isopropyl myristate). Beyond the significance in furthering our understanding of NMR sociality, the results of the study could have broad impact in the field of mammalian pheromones, the latter being the subject of considerable debate. For example, the characterisation of mammalian pheromones has remained elusive, even in mice, for which research has spanned many decades (see Wyatt 2014 for review) – with some authors even doubting their existence in mammals (in the social-insect sense of the definition), e.g. Doty (2010). To date, dominance-based behavioural interactions between the NMR queen and non-breeders have provided some explanation regarding the cues that bring about suppression, with a general agreement among NMR researchers that an individual odour "signature" (and possibly vocal cues) from the queen reinforces her presence in the darkness of the subterranean niche. This study goes further than this conjecture in implicating a single chemical rather than a learned odour profile. Many aspects of the study are robustly, logically and appropriately conducted and presented in a clear fashion. However, some aspects need much further clarification and justification and there are some weaknesses in the approach. In particular, the measures of reproductive activation of non-breeders lack detail/nuance (and even plausibility), and the relative contributions of the odour signal versus queen dominance interactions in suppression could or should be explored further. More care needs to be taken in social definitions and extrapolation across other species in the family. These and other issues are covered in more detail below.

RESPONSE: We thank the reviewer for these positive and constructive comments and for recognizing the potential significance of this work. We have carefully revised the manuscript in response, addressing the points raised through clarification, additional analyses, and new data where possible.

Specific points (minor)

1. Line 7: isopropyl myristate is stated to be volatile, yet in other places in the manuscript it is stated as low volatility (e.g. line 171).

RESPONSE: Isopropyl myristate is a low-volatility, long-chain ester, but it is nevertheless detectable in the headspace under our experimental conditions, as demonstrated by GC–MS sampling and by its ability to elicit olfactory responses across three independent assays. We have now changed it to low-volatility ester in the Abstract (Line 5).

2. Lines 15-16: "both eusocial species" only the NMR has been mentioned thus far in the abstract, this needs clarifying so it makes sense. Also see further discussion below regarding the validity of the statement "this mechanism enables mammalian eusociality".

RESPONSE: We have revised the Abstract (lines 10-12) to explicitly clarify this point by stating, *"Isopropyl myristate was also detected in breeding females of several Fukomys mole-rat species, but reaches higher levels in naked mole-rats, paralleling their extreme reproductive skew"* We have also revised the Results section to report the new comparative findings in detail (described explicitly below). In addition, we have toned down the wording regarding "enabling mammalian eusociality" to avoid overgeneralization and now frame this

mechanism more conservatively as being associated with species exhibiting extreme reproductive skew.

3. Lines 24-25: "division of labor into specialized roles" isn't strictly part of the accepted categorical definition of eusociality proposed by Michener, 1969.

RESPONSE: We have revised the text to align with the accepted definition, focusing on cooperative care of offspring, overlapping generations, and extreme reproductive skew, and have rephrased the text in lines 19-21 to: *"Eusociality is characterized by cooperative care of offspring, overlapping generations, and extreme reproductive skew, with reproduction typically monopolized by a dominant breeding female, ….."*

4. Lines 33-34: the statement "In mammals, such cues can trigger profound reproductive consequences, including pregnancy disruption (the Bruce effect)8, suppression of ovulation in subordinates9," is slightly misleading regarding Reference 9 (Barrett et al. 1990) and would benefit by being re-worded. Barrett et al. found a short extension of reproductive suppression (time to commencement of ovarian cyclicity) in subordinate female marmoset monkeys separated from their social groups but maintained in contact with the scent of the dominant breeder. Other cues (visual and behavioral) were also crucial in mediating full suppression (see follow on study by Barrett et al. 1993) as subordinate females with ablated olfactory epithelia within social groups stay suppressed, diminishing further the role of odour cues.

RESPONSE: We have revised the text in lines 32–33 to read "*suppression of ovulation in subordinates in conjunction with other social cues,*" thereby clarifying that olfactory cues act together with additional sensory and behavioural signals.

5. Line 48: isopropyl myristate was focused on as a prime candidate for a chemosignal presumably because in Figure 2c it is shown to be the most enriched compound in queens, although it could be argued that less enriched compounds could have greater effect depending on the sensitivity of the olfactory epithelium of NMRs to particular compounds. Notwithstanding that, "unknown 2" was also highly enriched. Why was that not chemically characterized and tested as well? If nothing else, it could have constituted a good control compound. A full tabulated list of compounds identified (as illustrated in extended data Fig. 2) would also be a useful addition in the supplementary section.

RESPONSE: Isopropyl myristate was prioritized because it was both strongly enriched in queens and could be unambiguously chemically identified and quantified using commercial standards. Although "Unknown 2" was also enriched, its mass spectrum indicated a long-chain compound with an unresolved double-bond position, which precluded definitive structural identification and the availability of a reference standard. As a result, we were unable to confidently characterize or test this compound in isolation.
We have now added a comprehensive tabulated list "**new Supplmetary Table 1**" of all identified compounds detected in Extended Data Fig. 2c, including their retention times, putative chemical classes, and identification confidence, in the Supplementary Information. We have also clarified this rationale in the Results text.

6. Line 53: The statement "uncovering a fundamental mechanism underlying mammalian eusociality" is arguably rather strong given that by the strict definition of eusociality (which many researchers disagree with using for mammals) the are only two "eusocial mammals" –

both mole-rats (NMR and Damaraland mole-rats) and the role of isopropyl myristate in the latter is far from proven (See point 15 below). I suggest toning down the wording.

RESPONSE: We appreciate the reviewer's concern regarding the ongoing debate surrounding the application of eusociality to mammals. Our intent was not to imply a universal mechanism across all mammals, but rather to highlight that a single, queen-derived chemical can mediate reproductive suppression in a mammalian system that meets commonly accepted criteria for eusociality. We changed the text in lines (51) to "*….underlying mammalian eusocial systems.*". We address this issue in more detail below (see point 15).

7. Lines 56-57: This ability has been tested before e.g. Clarke and Faulkes, 1999

RESPONSE: We have now cited Clarke and Faulkes (1999) in addition to the previously cited literature (O'Riain and Jarvis, 1997; Judd and Sherman, 1996; Toor et al., 2015) in the preceding sentence (lines 54).

8. Lines 64-66: How long does the OSN ablation last – is it permanent?

RESPONSE: We thank the reviewer for this question. Methimazole-induced ablation of olfactory sensory neurons is not permanent. We have elaborated on this point in the Methods section (lines 965-969), where we state: *"Following methimazole injury, the olfactory epithelium in mice regenerates over approximately one month, with newly generated olfactory sensory neurons beginning to establish synaptic contacts with the olfactory bulb within ~1–2 weeks[79]. Accordingly, all behavioural experiments were conducted within 3–7 days post-injection, the established window of maximal OSN loss, ensuring effective olfactory ablation."*

9. Lines 97-100: This description of the division of labor is outdated and not that simple – see Mooney et al., 2015, Gilbert et al., 2020, Holmes and Goldman 2021.

RESPONSE: We thank the reviewer for this clarification. We have rephrased the definition and deleted references to "division of labor" from the manuscript, and now cite recent work highlighting the complexity and flexibility of task specialization (Holmes and Goldman, 2021). Accordingly, we changed the text in lines (19-21) to "*Eusociality is characterized by cooperative care of offspring, overlapping generations, and extreme reproductive skew, with reproduction typically monopolized by a dominant breeding female, known as the queen, who is the sole reproducing female in the colony[2–4].*"

10. Lines 157-158 (and Fig. 2g legend): "Levels peaked during ovulation" - How was the time of ovulation determined in these females in order to collect the samples? This isn't explained (unless I missed it).

RESPONSE: Ovulation was determined retrospectively based on established reproductive indicators in naked mole-rats, including longitudinal hormonal profiling and reproductive state (pre-mating, mating, pregnancy, lactation), rather than direct physiological measurement at the time of sampling. We have now clarified this point in the Methods section in lines (916-919) to read as "*Ovulation was inferred retrospectively using established reproductive staging in naked mole-rats, based on longitudinal behavioural observations, and defined reproductive states (pre-mating, mating, pregnancy, and lactation)*".

11. Lines 161-163: "Naked mole-rats and Damaraland mole-rats (Fukomys damarensis) are the

only known eusocial mammals" this depends on your definition, see Crespi and Yanega (1995), among others.

RESPONSE: As noted above, we have rephrased the definition of eusociality to adopt more cautious and definition-aware language. Specifically, we revised the text in lines 19-21 to read: *"Eusociality is characterized by cooperative care of offspring, overlapping generations, and extreme reproductive skew, with reproduction typically monopolized by a dominant breeding female, known as the queen, who is the sole reproducing female in the colony*[2–4]*."*

12. Lines 164-165: "versus over 30% in Damaraland mole-rats" – check the 30%. I'm sure a much lower figure has been quoted before.

RESPONSE: We thank the reviewer for this comment. We have now deleted the whole sentence.

13. Lines 165-168: Again, it is difficult not to become bogged down with definitions like "social" versus eusocial (see point 11 above). There is a spectrum of sociality among Cryptomys and Fukomys, with overlap in terms of group sizes etc, and also evidence for physiological suppression (again, see point 15 below).

RESPONSE: As requested in point 15, we have expanded our comparative analysis to include three additional *Fukomys* species and re-ran a thorough cross-species analysis of isopropyl myristate abundance. In parallel, we have rephrased the text in lines (190-201) and lines (469-472) to emphasize graded variation in sociality and reproductive skew across mole-rat genera, acknowledging cooperative behavior, and physiological reproductive suppression. We address this issue in more detail below (see point 15).

14. Section starting line 196: I see why the odour receptor activation test had to be done, but in and of itself the results are perhaps not surprising as presumably many of the chemicals found (in extended Fig. 2) would potentially bind to an olfactory receptor and relay information to the olfactory bulb. That's how the sense of smell works. The vestigial VNO of NMRs is well known yet this is where a less volatile signal would normally be expected to act, and the pathways running to the accessory olfactory bulb then relaying information to the reproductive centres of the hypothalmus. If isopropyl myristate despite being "of low volatility" is acting via the primary olfactory epithelium and primary olfactory bulb, then this is highly unusual in terms of the accepted neural circuitry. Some mention of this might be helpful or worth brief discussion.

RESPONSE: In the revised manuscript, we now explicitly address this issue by (i) noting the vestigial nature of the vomeronasal organ in naked mole-rats; lines (203-204), (ii) showing minimal c-Fos activation in the accessory olfactory bulb following isopropyl myristate exposure **new Fig. 3c** and **new Extended Data Fig. 4e**, and (iii) demonstrating robust activation of olfactory regions using functional ultrasound imaging (fUSI); **new Fig. 3d-g** and **new Extended Data Fig. 5a**. Together, these data support the conclusion that, despite its low volatility, isopropyl myristate is detected predominantly via the main olfactory system.

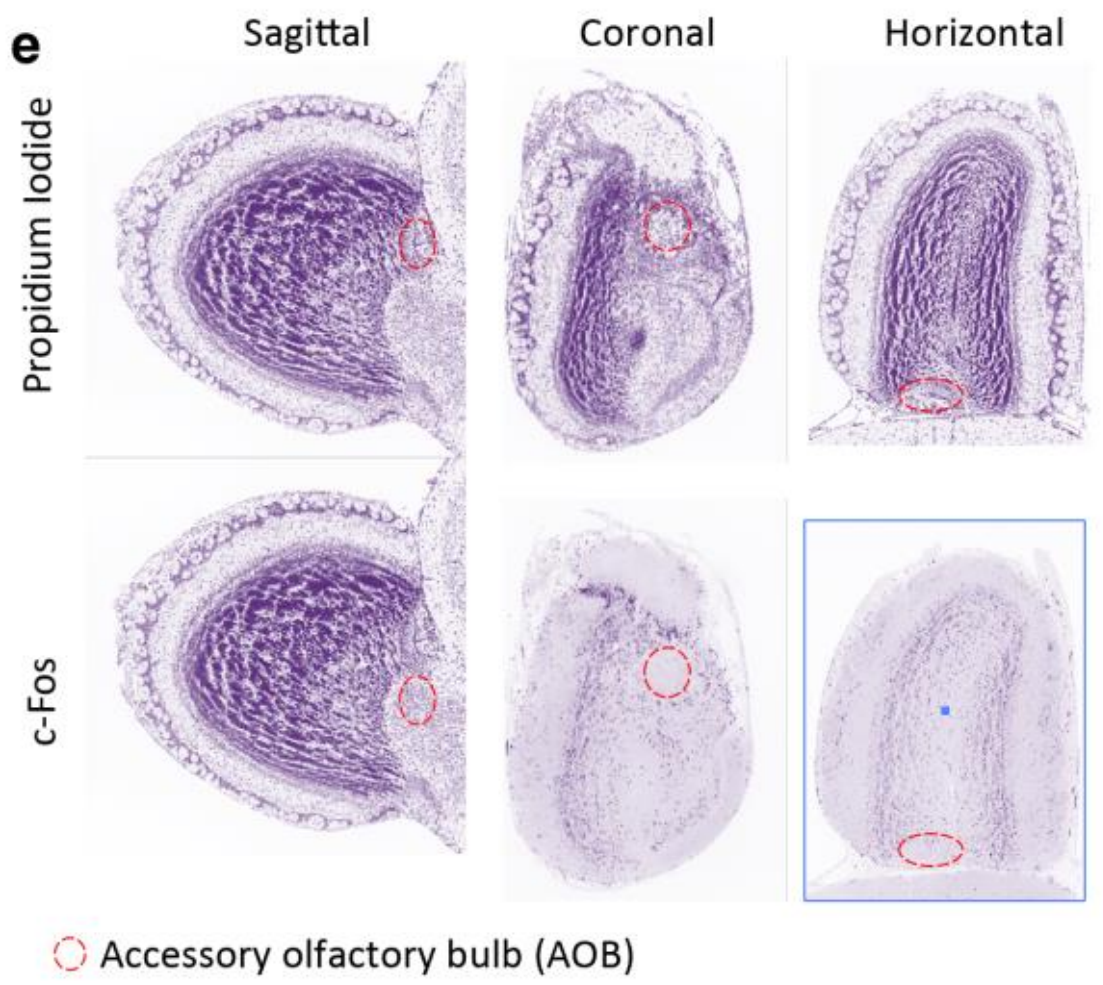

Extended Data Fig. 4e, Left, sagittal, coronal, and horizontal sections of the naked mole-rat olfactory bulb showing propidium iodide staining (top) and c-Fos expression (bottom); dashed circles mark the accessory olfactory bulb (AOB).

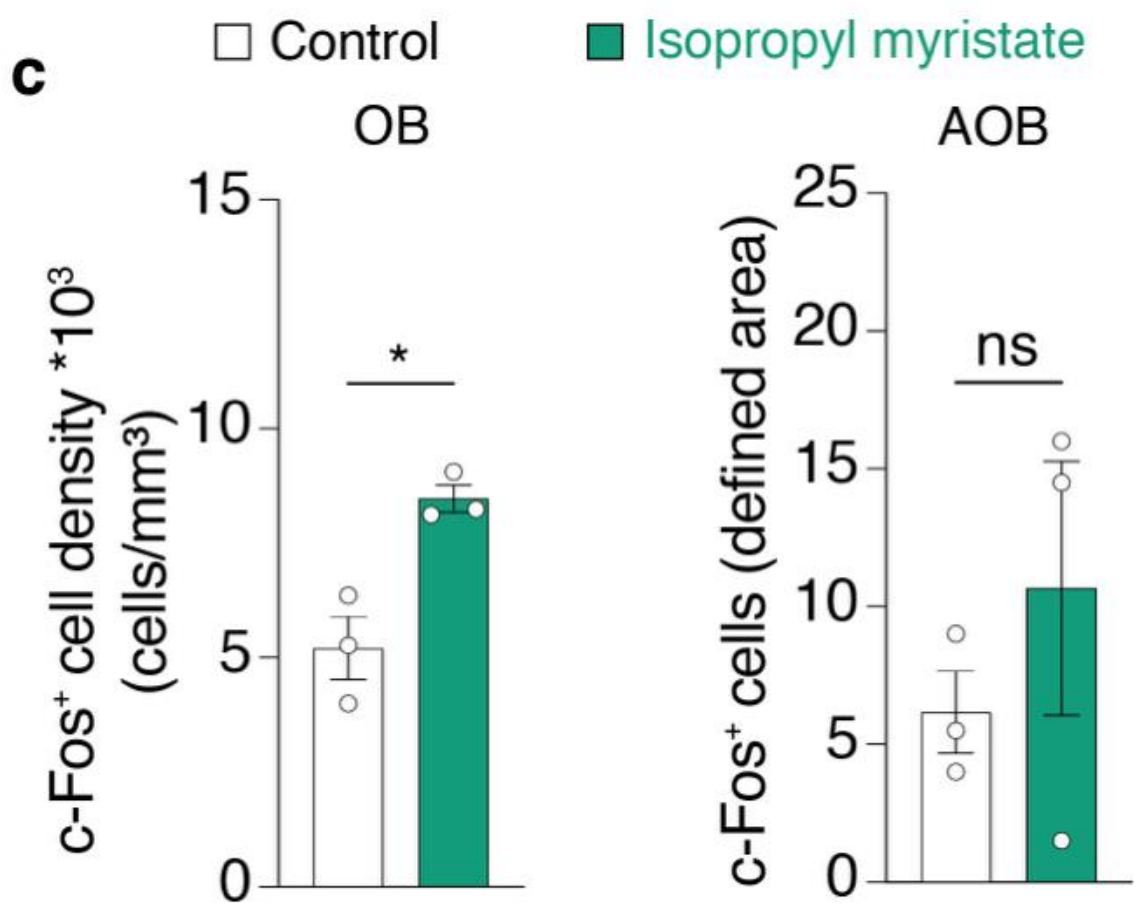

Fig. 3c, Left, c-Fos⁺ cell density (cells/mm³) in the olfactory bulb showed a significant increase following isopropyl myristate exposure compared to controls. Values were averaged across the left and right olfactory bulbs for each animal. Right, the average number of c-Fos⁺ cells across the left and right accessory olfactory bulb (AOB, the main target of vomeronasal organ sensory neurons) for control (air-exposed) and isopropyl myristate–exposed animals. Cells were quantified within anatomically defined AOB regions (see methods). Individual points denote animals; bars indicate mean ± s.e.m. Groups were compared using Mann–Whitney U test; ns * $p > 0.05$, ns = $p = 0.7$.

Specific points (major)

15. Lines 165-170: "Remarkably, isopropyl myristate was exclusively detected in the two eusocial mole-rat species, H. glaber and F. damarensis, but was absent in all other tested African mole-rat species (Extended Data Fig. 3e)." While this statement might be true, it doesn't tell the whole story, and more information and qualification of this statement is required. Firstly, the mechanism of suppression in F. damarensis females is different to the NMR (Bennett et al. 2022) and not prolactin driven, so any pathways of action of isopropyl myristate must be different in damarensis. More importantly, while several species of Cryptomys were screened for isopropyl myristate (and was found to be absent) only F. damarensis was examined among Fukomys. Within that genus the are many "social" species

including a number that are very close sister species to F. damarensis. The authors contentions would be much stronger (and more believable) if several other Fukomys species were screened for the presence of isopropyl myristate to prove that it has only evolved as a signal in F. damarensis, and not for example in the common ancestor of the Fukomys clade. Furthermore, on lines 48-49 it is stated that "isopropyl myristate, is uniquely present in queens, especially peaking in vaginal and anal secretions during reproductive readiness". Were the other species of mole-rat analysed for isopropyl myristate and illustrated in Fig. 3e also at these stages of their reproductive cycle? If not, what was their reproductive status? Presumably it could be missed. How many individuals of these other species were analysed? What sexes were they? None of this important information is given for these other eight species in the methods (line 737).

Janse van Vuuren et al. (2025) suggest a prolactin-mediated physiological-based socially induced infertility in highveld mole-rats (C. pretoriae) – the same as in NMRs. C. pretoriae are included among the other mole-rats screened for isopropyl myristate, yet was not found, arguing against a widespread function among mole-rats. This should be mentioned.

RESPONSE: We thank the reviewer for this detailed and important critique. We agree that the original statement was overly categorical and required additional qualification. In response, we have substantially expanded the comparative analysis and revised both the Results and Discussion accordingly.

- First, we have now **screened three additional *Fukomys* species** (*F. micklemi*, *F. darlingi*, and *F. mechowii*) in addition to *F. damarensis*, and re-ran a thorough GC–MS analysis across all sampled species. These data are now presented in the main manuscript (**new Fig. 2i,j**), revealing a graded distribution of isopropyl myristate: highest levels in naked mole-rats, but detectable levels in the four *Fukomys* species, and absence in *Cryptomys* and solitary genera. We have changed the text in lines (186-200) to read as "*Naked mole-rats represent the clearest example of eusociality in mammals. Consistently, isopropyl myristate abundance was highest in naked mole-rats, but it was also detected in reproductive females from all four Fukomys species tested, while being absent from Cryptomys and the solitary species (Fig. 2i,j). These findings indicate that isopropyl myristate production is not a general feature of mole-rat sociality, but may be enriched in social species exhibiting reproductive skew.*"

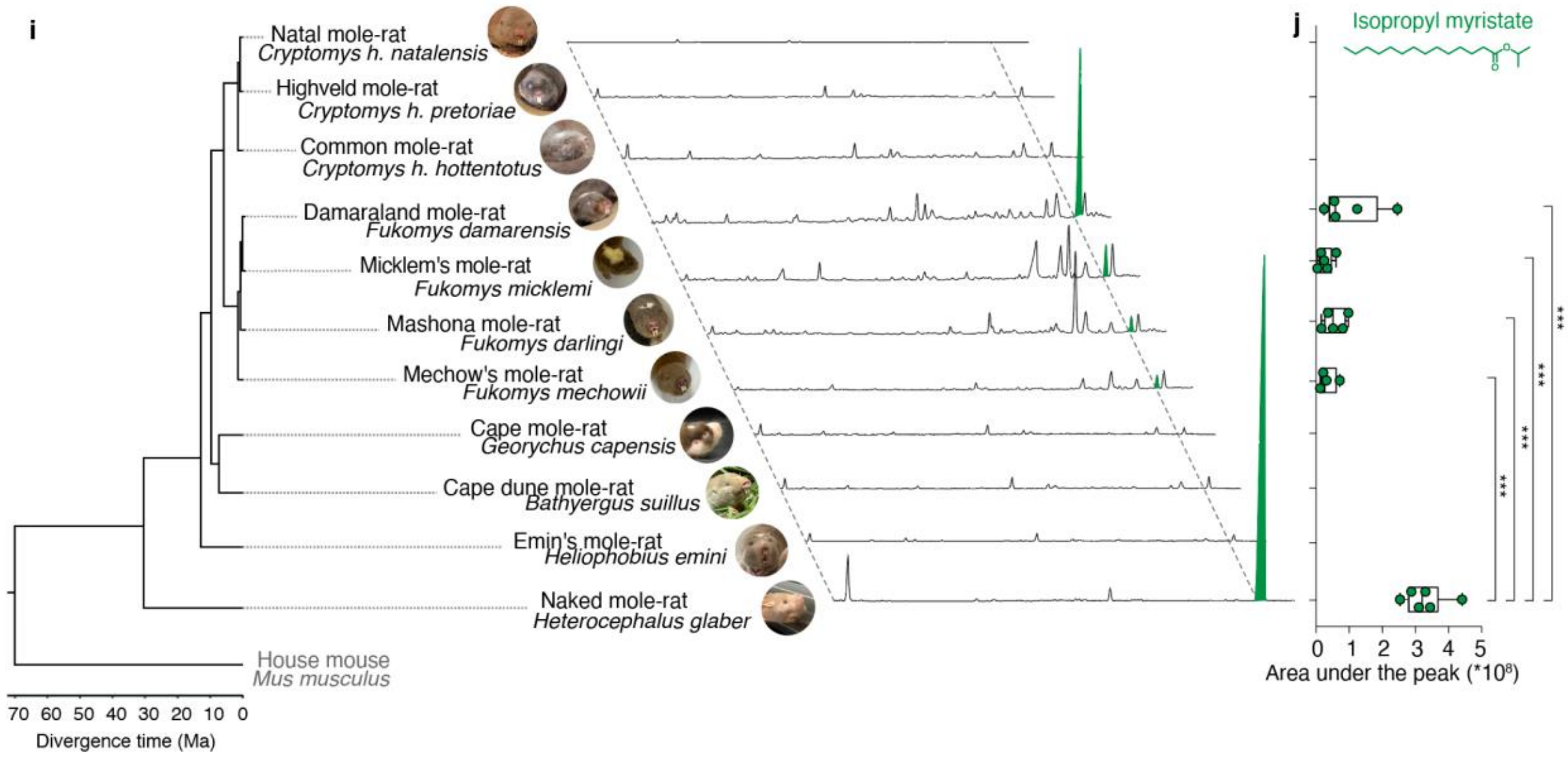

Fig. 2i, A time-calibrated phylogenetic tree of African mole-rats and the house mouse (*Mus musculus*) as outgroup with corresponding GC-MS chromatograms shows species-specific odour profiles. Chromatograms are aligned to

highlight the retention time of isopropyl myristate (green). Photo credits: Mohammed A. Khallaf, Alice Rossi, Andreas K. Janse Van Vuuren, Daniel Hart, Daniel Mendez Aranda, Firdevs Murad, and Felix Petermann.
j, Quantification of isopropyl myristate abundance (area under the peak) across species. Each dot represents a breeding female. Naked mole-rats isopropyl myristate levels were significantly higher compared to all *Fukomys* species. Levels of isopropyl myristate in Damaraland mole-rats did not differ significantly from those in other *Fukomys* species. *Cryptomys* and solitary species showed no detectable isopropyl myristate peak (one-way ANOVA with Holm–Sidak correction, ****p*< 0.001.).

- Second, we have revised the text to emphasize that the presence of isopropyl myristate is correlated with reproductive ske"Comparative analyses revealed that isopropyl myristate was detected in breeding females of a further four Fukomys species (Fig. 2i). The mechanisms of reproductive suppression likely vary between these four Fukomys species[64], but physiological suppression of reproduction with considerable reproductive skew, similar to naked mole-rats, has been described in F. damaransis[65]. Due to clear limits on the length of the text we have not gone into this issue in detail as we feel it is beyond the scope of this paper. Clearly the identification of isopropyl myristate will enable mechanistic studies in species of Fukomys on the role of this odourant in cooperative reproduction.

- Finally, we have clarified the sampling strategy and reproductive status of non-naked mole-rat species in the Methods. All comparative samples were obtained non-invasively from adult females of known breeding status where possible, and sample sizes and reproductive state are now explicitly reported in lines (922-930) "*Breeding females of the four Fukomys spp. were sampled across two reproductive stages (ovulation and pregnancy), as were naked mole-rats. The three Cryptomys spp. females were likewise sampled during ovulation and pregnancy, whereas Bathyergus females were sampled during lactation. We could not identify the reproductive stage of Georychus spp. Importantly, isopropyl myristate was detectable across all sampled reproductive stages (Fig. 2f), but its abundance was most strongly elevated during ovulation, indicating that the observed species differences are not attributable to mismatched reproductive staging. Reproductive state in all breeding females was retrospectively determined using established morphological and behavioural criteria.*"

16. Line 285 onwards to the discussion and 702-716: In my opinion, the measures of reproductive activation in the separation and scent/bedding transfer experiments are not detailed enough and some measures are unconvincing at best. Granted, pregnancy appears to be suppressed. However, important subtleties are lost. Firstly, the authors state "reproductive activation (testis enlargement, vaginal perforation, copulatory plug formation) was documented photographically". How can testis enlargement be measured in a small rodent that does not have an external scrotal sac? The testes in NMRs remain deeply abdominal and do not descend. In non-breeder males they are average mass of 0.034 g/testis - even in breeders they are very small (testis mass around 0.079 g/79 mg) and could surely only be measured by a highly sensitive ultrasound device. How could testes enlargement possibly have been "documented photographically"? Can the measurements be provided? Furthermore, while occasionally a crusty vaginal discharge can be seen in queens, copulatory plugs as described for other mammals (to prevent sperm competition) do not form in NMRs (Seney et al. 2009). External genitalia is a difficult way of assessing either sex in NMRs, and vaginal development and perforation can also be very variable among females in a colony - separated singly housed females can initially undergo clear ovarian cycles (indicated by elevated progesterone) yet remain imperforate with no male present and thus no mating. The photos in Extended Data Fig. 5e are not overly convincing without "before" and "after" separation pictures for all the animals concerned and some kind of quantitative measure. Without this it is all very subjective.

Without hormonal profiling (e.g. testosterone in males and progesterone in females) or post-mortem gonadal histology in the experimental (bedding transfer/isopropyl myristate) treatment conditions to look at gonadal activation it is impossible to tease apart sex differences and/or more subtle yet important effects of suppressing cues. For example, one scenario could be that the separated females in scent contact began ovarian cycling (i.e. were released from suppression) yet did not become pregnant because just the males were suppressed by the isopropyl myristate. Reading the paper cited on line 375 (Faulkes et al., 1993), of the bedding transfer experiments reported therein only one of the six treatment females actually became pregnant (91 days after pairing with a male), yet all the separated treatment females receiving bedding transfer showed ovarian cyclicity (as evidenced by progesterone profiles). This emphasises the disadvantages of relying on pregnancy as the only clear marker of reproduction in the manuscript under review. Why were hormonal profiling or post-mortem gonadal histology omitted from the study? It seems a major and obvious omission.

It is also not clear how two-queen colonies can form (although uncommon, they do occur) with females sharing the nest and in close body (and chemical) contact. Likewise, how does the queen recruit a breeding male from a non-breeder when characteristically they spend much time in close body/chemical contact?

On lines 285-287 it states "Prolactin levels in subordinates were significantly higher during the queen's ovulatory and pregnancy phases, aligning with peak isopropyl myristate colony-abundance (Fig. 4c)" but the figure legend states this was only in females – this needs clarifying. What about males? Previous work suggests that as the queen approaches the end of gestation she may lose some control of the colony (in terms of suppression) – what stage of pregnancy is reflected in 4c? Higher prolactin would infer increased suppression and be at odds with these earlier observations.

On lines 295-301 it states that "Pairs were randomly assigned to one of three treatment groups", but how were the male-female pairs initially chosen for treatment assignment in terms of social rank, body size, home or different colony? All the aforementioned could affect pairing success. Was it andom as well. As worded, it is not clear.

On lines 305-307 - see comment above regarding measures of activation and plugs, and note on line 309 that mating is very difficult to observe unless the animals are being extensively observed, so not a good marker of reproductive activation.

The statement on lines 346-347 and content on Figure 5 may need rewording in the light of comments above and to follow.

RESPONSE: Thank you for this detailed and important critique. We agree that reliance on pregnancy outcomes and external genital morphology alone is insufficient to capture the subtleties of reproductive activation and suppression in naked mole-rats. In response, we have substantially expanded the experimental readouts and clarified methodological details, as outlined below:

1. Clarification of genital measures and removal of over-interpretation
   We agree that testis "enlargement" cannot be reliably quantified photographically in naked mole-rats, whose testes remain intra-abdominal and do not descend. Accordingly, we have removed claims of testis enlargement as a primary measure and now treat external

genital morphology (including vaginal perforation and genital appearance) as qualitative indicators only, reported conservatively and not used as sole evidence of reproductive activation. We have also clarified that copulatory plug–like material was noted descriptively and do not imply functional plug formation as described in other rodents (Seney et al., 2009). The new texts as follow in:

- Results, lines (888-890): "…, *reproductive activation was assessed using endocrine measures and qualitative external genital observations, including vaginal perforation and copulatory plug formation, which were documented photographically*".

- Methods, lines (884-886): "*Because testes in naked mole-rats remain intra-abdominal, external genital morphology was used only as a qualitative indicator and not as a quantitative measure of gonadal activation*".

- Figure legend, lines (1295-1294): "*Breeding males and females display qualitative changes in external genital appearance consistent with reproductive status.*"

2. **Addition of longitudinal endocrine profiling (new data).** Critically, to address the reviewer's central concern, we have now added longitudinal hormonal profiling using non-invasive fecal steroid measurements, which provide objective, quantitative markers of reproductive activation:

- **Fecal progesterone metabolites (fPMs).** in females demonstrate that ovarian cycling and reproductive activation occurred only in the blank condition, while remaining suppressed in females exposed to colony bedding or isopropyl myristate (**new Fig. 4g, k**).

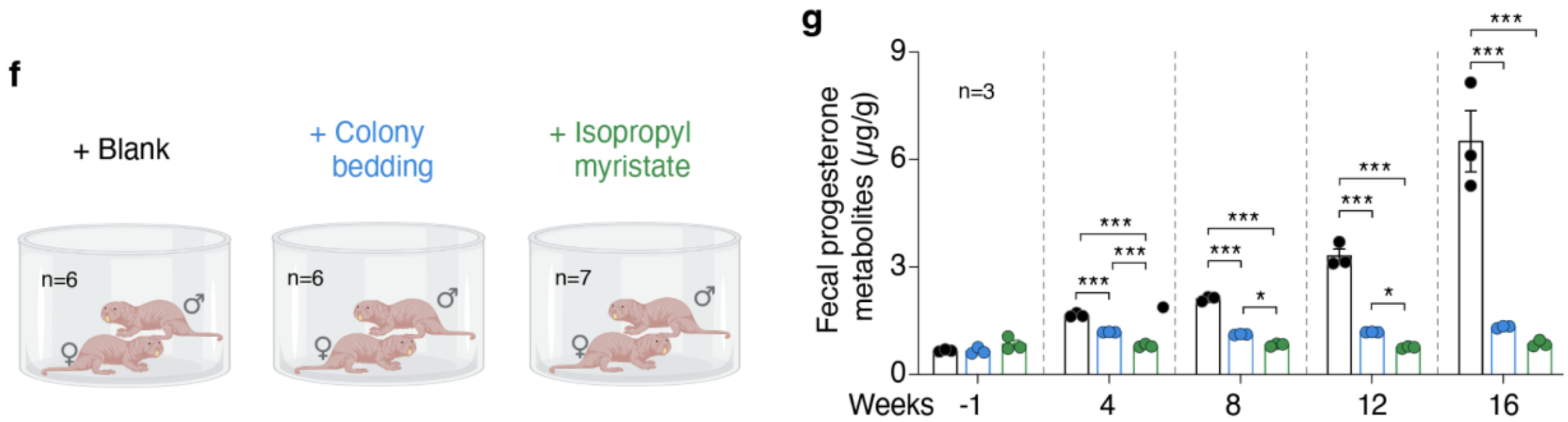

Fig. 4f, Experimental design showing opposite-sex subordinate pairs housed with blank, bedding from their natal colony, or daily exposure to isopropyl myristate (425mg).
g, Longitudinal measurements of fecal progesterone metabolites show a progressive increase only in the blank group, indicating reproductive activation. Each dot represents an individual female; one way ANOVA with Holm–Sidak correction ***$p< 0.001$.

- **Fecal testosterone metabolites (fAMs)** in males were significantly reduced by colony bedding and isopropyl myristate compared with blank controls (**new Extended Data Fig. 6c**); however, this effect may reflect female reproductive state or social context, rather than a male-specific action of isopropyl myristate alone.

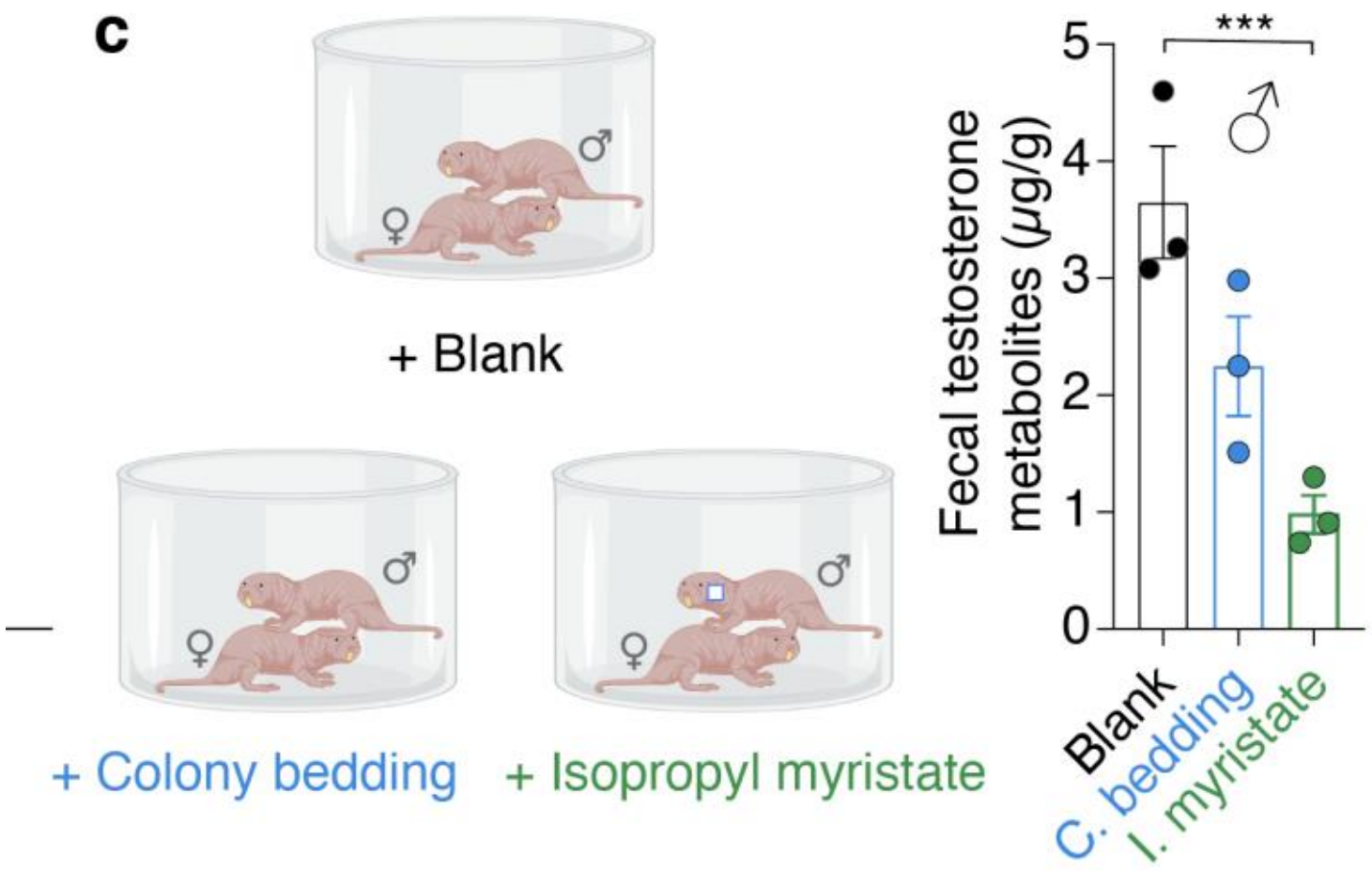

Extended Data Fig. 6c, Fecal testosterone metabolite levels in males housed with blank bedding, colony/queen bedding, or isopropyl myristate. Testosterone metabolites are significantly elevated in the blank condition compared with colony bedding and isopropyl myristate; one way ANOVA with Holm–Sidak correction ***$p < 0.001$.

3. **Pregnancy is no longer treated as the sole endpoint**. We now explicitly frame pregnancy as a downstream outcome, rather than the primary readout, and interpret it alongside endocrine measures (for example, **new Fig.4i-k**), body mass trajectories, and longitudinal hormone changes. This directly addresses the reviewer's comparison to Faulkes et al. (1993), where ovarian cyclicity occurred without pregnancy. We also stated that in the Methods, lines (875-877): "*Pregnancy outcomes were interpreted as a downstream consequence of reproductive activation and were evaluated alongside longitudinal endocrine measures and body mass trajectories.* "

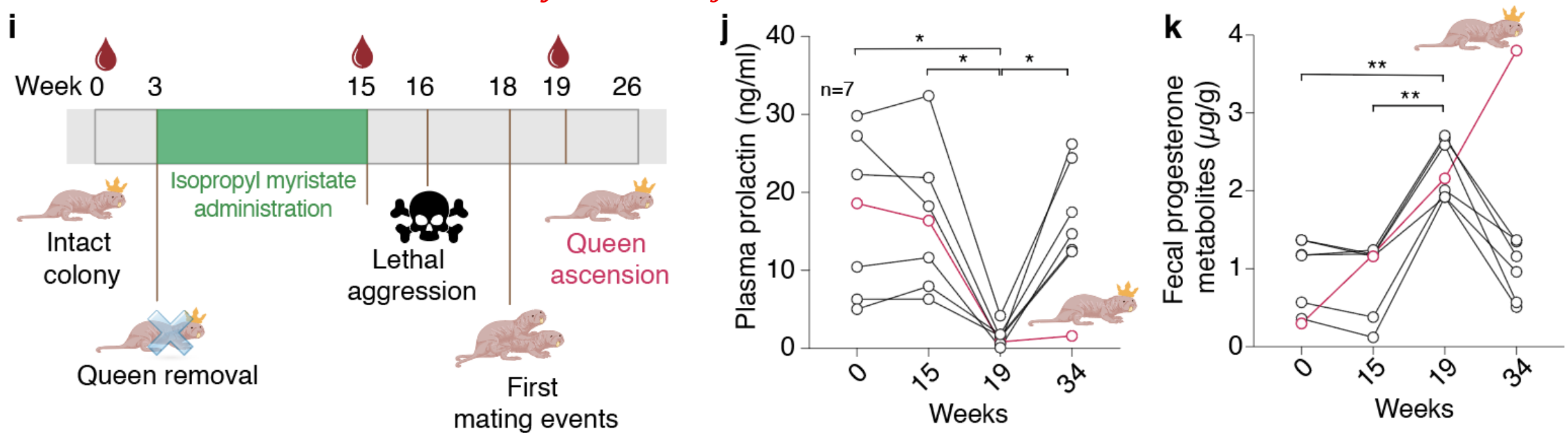

Fig. 4i, Experimental timeline following queen removal at week 3, with daily administration of isopropyl myristate (425mg) for 12 weeks. Key events included lethal aggression at week 16 upon cessation of isopropyl myristate exposure, first mating events at week 18, and queen ascension by week 26. Blood samples were collected at indicated time points (red drops).
j, Plasma prolactin levels dropped significantly by week 19 after isopropyl myristate withdrawal and increased again by week 34, except in one female (raspberry). Repeated measures ANOVA with Holm–Sidak correction, *$p < 0.05$.
k, Longitudinal changes in fecal progesterone metabolites following queen removal. Fecal progesterone metabolite levels increased significantly by week 19 after isopropyl myristate withdrawal and remained elevated in one female (raspberry), coinciding with her ascension to queen status. Repeated-measures ANOVA with Holm–Šidák correction,

4. **Pairing criteria and randomization clarified.** Opposite-sex pairs were formed from low-ranking non-breeders, matched for body size and drawn from the same colonies to avoid confounds related to isolation history. Pairs were then randomly assigned to treatment groups. This information is now stated in the Methods, explicitly to avoid ambiguity.

5. **Sex specificity of prolactin data clarified**. We have clarified that Fig. 4c initially reports prolactin levels in females, and we now include male prolactin measurements (**new Extended Data Fig. 6b**). We also clarify the pregnancy stage represented in Fig. 4c (early–mid gestation) resolving the apparent discrepancy raised by the reviewer.

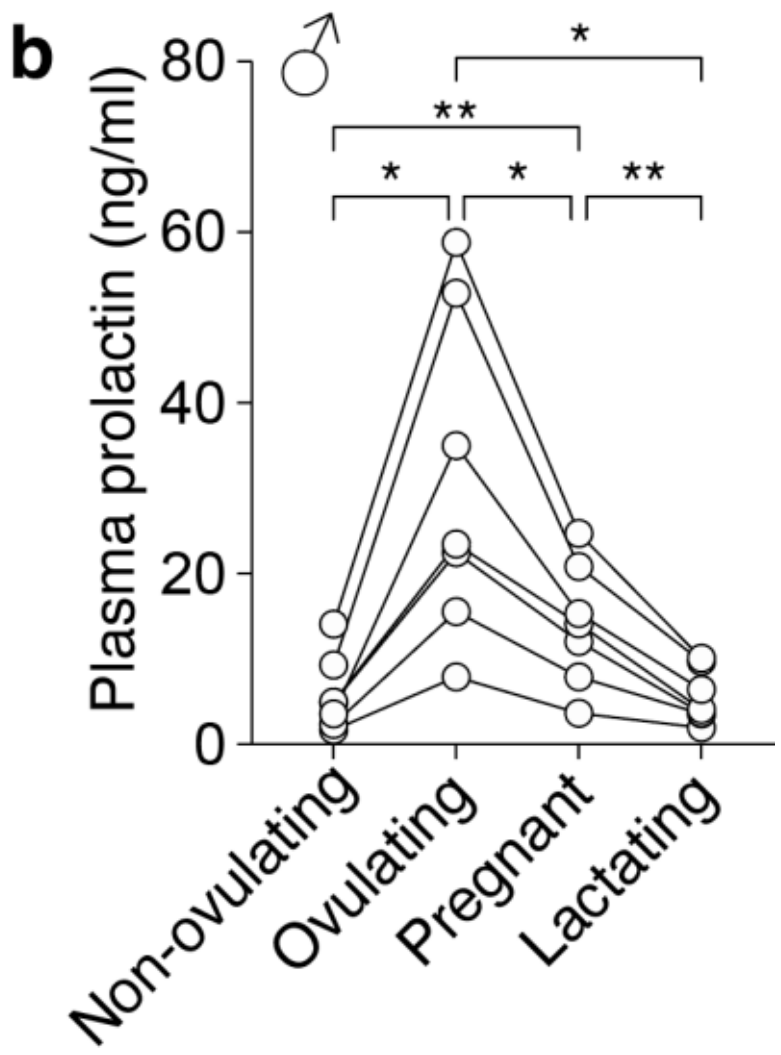

Extended Data Fig. 6b, Plasma prolactin levels in males across the queen's reproductive states. Each line represents an individual; repeated measures ANOVA with Holm–Sidak correction *$p < 0.05$, **$p < 0.01$.

6. **Two-queen colonies and breeder recruitment**. Rare cases of plural breeding in naked mole-rat colonies indicate that reproductive suppression is not rigid but can be relaxed under specific conditions. Recent study (Abeywardena et al., 2026; DOI: 10.64898/2026.01.09.698689, available on bioRxiv) shows that the emergence of two reproductive females occurs when the reproductive capacity of the reigning queen is impaired, for example by environmental stressors. Relocation to a new facility represents a potent environmental stressor that can impair the reproductive function of the reigning queen without disrupting social dominance. However, the presence of Two-queen colonies and whether such environmentally induced, non-aggressive plural breeding occurs under natural conditions remains an open question.

7. **Post-mortem histology**. We agree that gonadal histology would provide valuable additional resolution. However, given the longitudinal nature of the experiments and the ethical constraints associated with colony-level manipulations in naked mole-rats, post-mortem sampling was not feasible in the present study. Importantly, colonies in which breeding occurred in the control groups were required to be maintained intact, as euthanizing reproductive individuals (including the queen) would have permanently terminated the colony and precluded further longitudinal analysis.

8. **Updated Fig. 5 and associated text**. In the revised manuscript, Fig. 5 has been updated from a pregnancy-centered schematic to an integrative model that summarizes reproductive suppression using longitudinal endocrine, physiological, and behavioural data, explicitly avoiding reliance on any single metric to define reproductive activation.

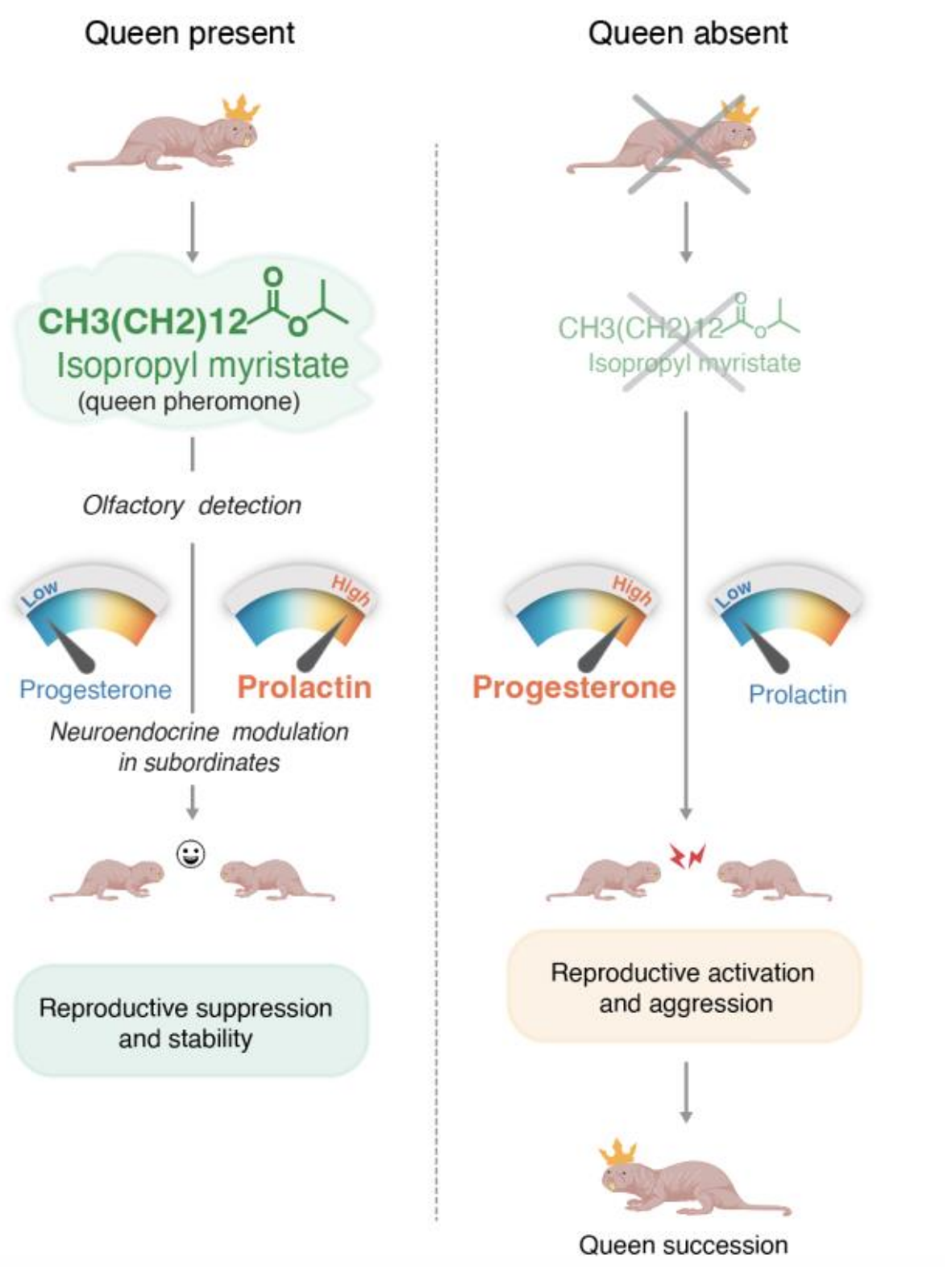

Fig. 5: Isopropyl myristate links queen presence to prolactin elevation and progesterone suppression. Schematic illustrating contrasting social and physiological outcomes in naked mole-rat colonies in the presence (left) and absence (right) of the queen. When the queen is present, she produces isopropyl myristate, which is detected via olfaction and elevates prolactin while suppressing progesterone in subordinates, maintaining reproductive suppression and social stability. Upon queen loss, isopropyl myristate levels decline, prolactin decreases and progesterone increases, triggering reproductive activation and aggression that can result in mortality. Ultimately, one dominant female ascends to reproductive status and succeeds as the new queen.

17. The study needs further nuancing to fully address the relative the effects of queen behavior and odour signalling and a crucial experiment remains to be done. While animals have been separated and kept in odour contact, and in one colony the queen was removed but isopropyl myristate added to maintain suppression, anosmic NBrs (methimazole ablated) should have been investigated (with appropriate profiling) while remaining as subordinates within their colonies in a similar way to the marmoset study of Barrett et al. (1993) – where anosmic subordinate females stayed suppressed because behavioral and other cues over-rode the effect of odour. Only by doing this can the effects of dominance and queen odour be teased apart.

RESPONSE: We agree that such an experiment would further disentangle the relative contributions of queen behaviour and olfactory cues. However, performing methimazole-induced anosmia in subordinate animals while maintaining them within intact colonies would require prolonged colony-level manipulation. Importantly, methimazole-induced olfactory sensory neuron ablation is transient, with OSNs beginning to regenerate within 1–2 weeks, thus for such an experiment animals would have to receive multiple methimazole injections which is likely to be highly stressful and we do not have ethics permission for such an experiment. Multiple methamizole injections could cause stress which would complicate long-term behavioural and endocrine interpretations in a social colony context. For these reasons, this experiment is not feasible within the scope of the present study.

References

Barrett J, Abbott DH, George LM. (1993) Sensory cues and the suppression of reproduction in

subordinate female marmoset monkeys, Callithrix jacchus. J Reprod Fertil. 97(1):301-10. doi: 10.1530/jrf.0.0970301. PMID: 8464022.

Bennett, N.C.; Faulkes, C.G.; Voigt, C. (2022) Socially Induced Infertility in Naked and Damaraland Mole-Rats: A Tale of Two Mechanisms of Social Suppression. Animals 2022, 12, 3039. https://doi.org/10.3390/ani12213039

Clarke F. M. and Faulkes C. G. (1999) Kin discrimination and female mate choice in the naked mole-rat Heterocephalus glaber. Proc. R. Soc. Lond. B.2661995–2002.

Crespi B.J., Yanega D. (1995) The definition of eusociality. Behavioral Ecology 6:109-115
Doty, R. L. (2010). The Great Pheromone Myth. Johns Hopkins University Press.

Gilbert J.D., Rossiter S.J., Faulkes C.G. (2020) The relationship between individual phenotype and the division of labour in naked mole-rats: it's complicated. PeerJ 8:e9891 https://doi.org/10.7717/peerj.9891

Holmes, M.M., Goldman, B.D. (2021). Social Behavior in Naked Mole-Rats: Individual Differences in Phenotype and Proximate Mechanisms of Mammalian Eusociality. In: Buffenstein, R., Park, T.J., Holmes, M.M. (eds) The Extraordinary Biology of the Naked Mole-Rat. Advances in Experimental Medicine and Biology, vol 1319. Springer, Cham.

Janse van Vuuren, A.K., Süess, T., Finn, K., Hagenah, N., Ganswindt, A., Hart, D.W., & Bennett, N.C. (2025). Hormonal lockdown: How mole-rat societies enforce infertility in helpers. Hormones and Behavior, 147, 105836.

Mooney S.J., Filice D.C.S., Douglas N.R., & Holmes M.M. (2015) Task specialization and task switching in eusocial mammals. Animal Behaviour 109:227-233

Seney, M. L., Kelly, D. A., Goldman, B. D., Sumbera, R., and Forger, N. G. (2009). Social structure predicts genital morphology in African mole-rats. PLoS One 4:e7477. doi: 10.1371/journal.pone.0007477

Wyatt, T. D. (2014). Pheromones and animal behavior: Chemical signals and signatures (2nd ed.). Cambridge University Press.

Referee #3 (Remarks to the Author):

Khallaf et al from the Lewin lab report the identification of a compound released by a female naked mole-rat, the queen "odor", that suppresses reproduction in other females in the same colony. This is a spectacular finding, which solves a long-standing mystery of how a single female is able to dominate and control reproduction in this eusocial mammal. The authors first show that naked mole-rats use chemical cues presumably detected by the olfactory epithelium to recognize other individuals, and that aggression between individuals is reduced when the olfactory epithelium is ablated with methimazole, which effectively ablates the olfactory epithelium. They next analyze compounds released by naked mole-rats, and show that Isopropyl myristate is selectively released by queens, and release is higher during ovulation. Most remarkably, they show that isopropyl myristate is sufficient to maintain females in a non-reproductive status. When the queen is removed and isopropyl myristate is introduced, the remaining females stay in a non-reproductive status (smaller, high prolactin levels). Upon removal of isopropyl myristate, prolactin levels in all females drops, the females begin to fight and a single female ascends. They also show that isopropyl myristate is sufficient to prevent females removed from the colony from becoming pregnant when placed with a male naked mole-rat. Overall, notwithstanding some concerns raised below, the authors provide convincing data that they have identified the mechanism that supports reproductive suppression in this eusocial mammal. The manuscript is well written and will be of great interest to a general scientific readership.

RESPONSE: We thank the reviewer for these generous and encouraging comments. We believe that the revisions have further solidified our conclusions and enhanced the clarity and impact of the study for a broad readership.

Concerns:

1. The authors did not in my view adequately address the question of whether the isopropyl myristate could be detected by the vomeronasal rather than olfactory system. Although the author cite evidence that the VNO is likely to be non-functional, based on its smaller size relative to other rodents, this does not rule it out as a possible site of sensory detection. One way to answer this question would be to look at whether methimazole causes cell death in the VNO, or if it spared. If spared, then the evidence would point clearly to detection by the olfactory epithelium. If ablated, then I believe the jury is out as to where the compound is detected.

RESPONSE: We thank the reviewer for this important point. We agree that the reduced size of the vomeronasal organ (VNO) in naked mole-rats does not, on its own, exclude a possible role in chemosensory detection. However, we provide a **new line of evidence** indicating that isopropyl myristate is primarily detected via the **main olfactory system**. whole-brain c-Fos mapping revealed a significant activation in the **main olfactory bulb**, while c-Fos expression in the **accessory olfactory bulb was minimal and did not differ from controls** (**<u>new</u> Fig. 3c**; **<u>new</u> Extended Data Fig. 4e**) (see response to reviewer no. 1; page 12).

We agree that assessing whether methimazole spares or ablates the VNO would further strengthen anatomical resolution of the sensory pathway. However, methimazole induces broad cytotoxicity within the nasal epithelium, and its effects on the VNO epithelium in naked mole-rats have not been systematically characterized. As such, we did not attempt to use methimazole ablation to draw definitive conclusions about VNO involvement.

We believe that the whole-brain functional imaging provides the strongest currently feasible evidence that isopropyl myristate acts predominantly through the main olfactory system, while leaving open the possibility of additional chemosensory contributions to be addressed in future studies.

Similarly, the evidence that the olfactory systems is specifically activated by isopropyl myristate (Fig 3) is not entirely convincing. In fig 3e, the response to Isopropyl myristate appears to encompass much of the olfactory bulb. Extended fig 4 e shows some evidence of higher labeling of some glomeruli, but it is hard to tell if that is just normal variation in staining. As this experiment was presumably repeated multiple times, I suggest the authors examine and show the cFos staining from all animals studied. This would allow them to address whether the activated glomeruli show a stereotyped localization, as expected if the compound is truly acting on a single or small number of odorant receptors.

RESPONSE: We agree that c-Fos–based mapping alone provides limited spatial resolution for determining whether isopropyl myristate activates a single or small number of odorant receptors. In the revised manuscript, we have therefore softened the interpretation of the c-Fos data and no longer use it to argue for stereotyped glomerular activation.

We now show minimal and unchanged activation of the accessory olfactory bulb following isopropyl myrsitate compared to controls (Fig. 3c; **new Extended Data Fig. 4e**) (see response to reviewer no. 1; page 12). As the reviewer notes, c-Fos labeling was sparse and variable across animals. To address this limitation directly, we complemented the c-Fos analysis with functional ultrasound imaging (fUSI), which provides repeated, within-animal, whole-brain functional measurements with high spatiotemporal resolution. fUSI revealed robust, solvent-independent, and reproducible activation in olfactory regions in response to isopropyl myristate, whereas air and DMSO controls were indistinguishable (**new Fig. 3d-g**; **new Extended Data Fig. 5a**) (see response to reviewer no. 1; page 4). Importantly, these effects were consistent across animals and quantified statistically, providing a stronger functional readout of olfactory system activation than c-Fos mapping alone.

Taken together, we now frame the c-Fos data as supportive but not definitive, and rely primarily on the convergent evidence from EOG recordings and fUSI to demonstrate that isopropyl myristate activates the olfactory system.

If the authors cannot provide evidence to distinguish between detection by the olfactory epithelium and the VNO, they should state this. This would in no way detract from the main conclusion of the paper, showing a clear role for a chemical cue, isopropyl myristate, in the maintenance of reproductive suppression in naked mole-rats

RESPONSE: We agree with the reviewer. In the revised manuscript, we now explicitly state that while our data strongly support detection of isopropyl myristate via the main olfactory system, we cannot definitively exclude a contribution of the vomeronasal system, lines (414-418). We emphasize that this limitation does not affect the central conclusion of the study, namely that isopropyl myristate functions as a queen-derived chemical cue that maintains reproductive suppression and social stability in naked mole-rats. "Using several methodological approaches including non-invasive fUSI brain imaging we demonstrate that isopropyl myristate elicits robust activation of olfactory neurons and central circuits providing a neural substrate for its colony-wide effects. Our data are consistent with detection of

isopropyl myristate via the main olfactory epithelium, however, we cannot exclude a contribution of the vomeronasal system."

Minor:
Fig 3a. The cartoon looks like the recording was made in vivo but the text says ex vivo

RESPONSE: We have now replaced the schematic with a representative ex vivo image of the olfactory epithelium preparation (**new Extended Data Fig. 4a**).

Fig 3b. What is the thick black line – is this the average response? What does the DMSO control look like?

RESPONSE: The thick black line indicates the mean EOG response across animals; thin lines show individual traces. DMSO control responses are now shown and do not differ from air controls (**new Extended Data Fig. 4b,c**).

Fig 4e. This result is not well described. Are these females that were removed from the colony (from Fig 4d), who were treated with methimazole? Or just some of the females in the colony. Showing a timeline of the experiment would help.

RESPONSE: We have now added an experimental timeline to improve clarity.

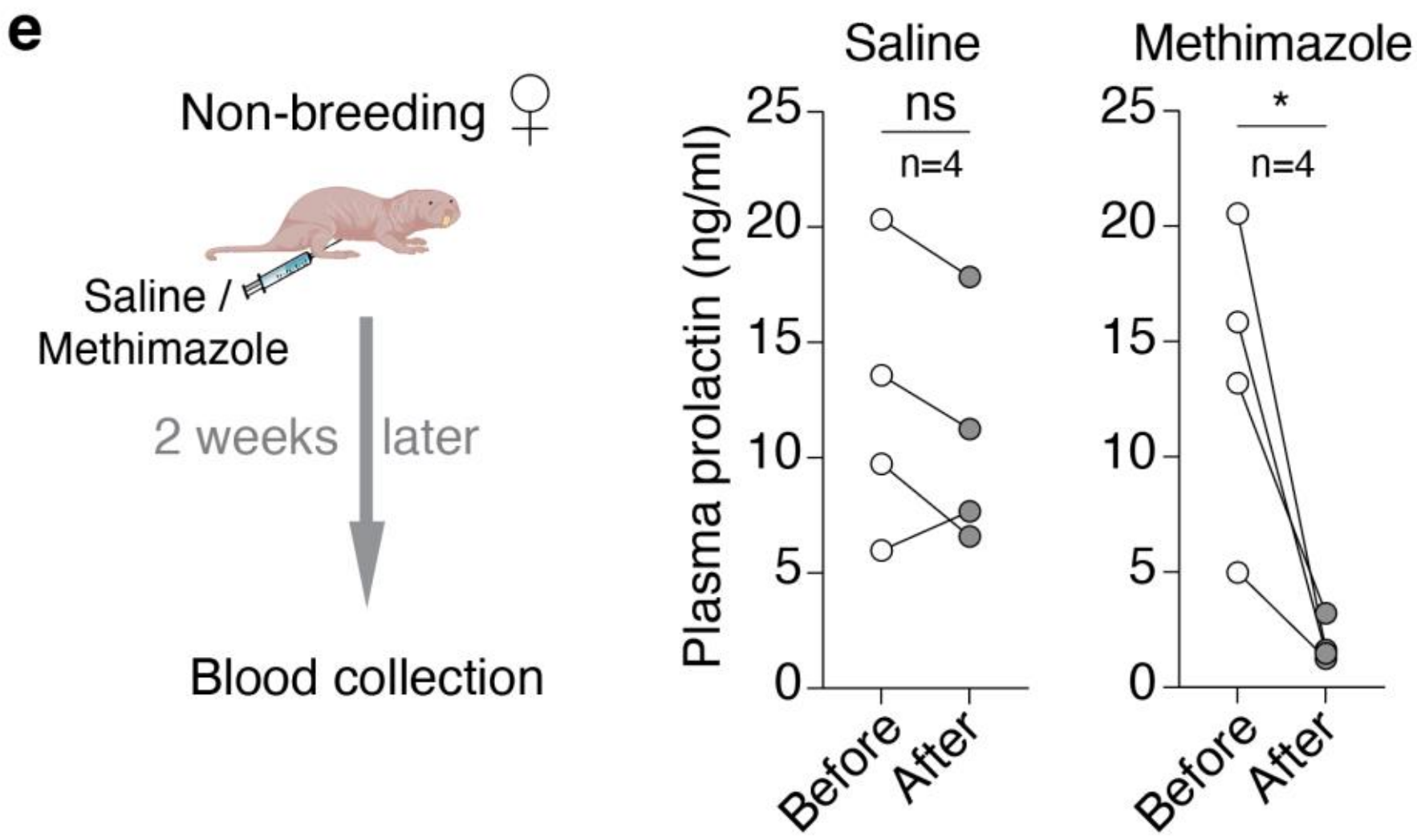

Fig. 4e, Schematic of treatment timeline and plasma prolactin levels before and after saline or methimazole. Methimazole treatment significantly reduced prolactin levels, while saline had no effect. Each line represents one individual. Paired *t*-test $*p < 0.05$.

Nature 2025-09-24315A: RESPONSE TO REVIEWERS

We are grateful to the reviewers for their encouraging and insightful evaluation of our manuscript, "*A queen odour mediates reproductive suppression in a eusocial mammal.*" We are encouraged by their positive assessment of the work.

In particular, Reviewer 1 described the revision as comprehensive and expressed strong enthusiasm for the work, and Reviewer 3 also indicated support for publication. Reviewer 2 further noted that the manuscript has been substantially strengthened by the revisions.

In response to the reviewers' suggestions, we have revised the manuscript to clarify several points, moderate specific interpretations, add new data, and improve the presentation of the results. Below we provide a detailed point-by-point response to all comments.

Referee #1 (Remarks to the Author):

The authors have done a comprehensive and impressive revision of the paper, have addressed all of my prior concerns, and I am enthusiastic about the work. The fUSI is remarkable and very informative, and I appreciate the revised EOG data. I defer to the authors on the other minor recommendations that they chose to not pursue.

Review prepared by Leslie Vosshall

RESPONSE: We thank the reviewer for the positive evaluation and are pleased that the fUSI experiments were found informative.

Referee #2 (Remarks to the Author):

The manuscript is substantially improved and stronger than the original submission. In particular, the expanded comparative analysis across multiple Fukomys species resolves issues with the evolutionary framing. The addition of longitudinal fecal steroid profiling strengthens the interpretation of reproductive activation and suppression (although this is only provided for n=3 pairs), and the functional ultrasound imaging is an interesting inclusion. These additions address several of my primary concerns from the first round of reviewing. However, despite these improvements, the manuscript still overstates the exclusivity and mechanistic sufficiency of isopropyl myristate in a number of places. For a study of this conceptual impact particularly in a journal such as Nature, the strength of the claims must match the strength of the evidence in the absence of the key within colony anosmia experiment that I suggested.

RESPONSE: We thank the reviewer for the positive assessment and for noting that the additional analyses strengthened the study. We have accordingly moderated the language to acknowledge that additional social and chemical cues may contribute to reproductive suppression in intact colonies.

Remaining issues that require revision are detailed below:

1. Overstatement of exclusivity of isopropyl myristate and mechanism
The manuscript repeatedly uses language implying that isopropyl myristate governs, enforces, or orchestrates reproductive suppression in naked mole-rats.
For example:

The Abstract states that the study reveals “a single chemical cue governing reproductive suppression”.
The Discussion refers to “a solitary pheromone that can enforce reproductive hierarchies, prevent conflict, and sustain eusociality.”
The Results state that isopropyl myristate “alone was sufficient” to preserve reproductive suppression.
Other prominent compounds in the queen odour profile were not tested, so there could also be further compounds that reflect the presence of a breeding queen.
The statements above imply that isopropyl myristate alone is both necessary and sufficient for reproductive regulation in intact colonies. However, the key experiment disentangling olfactory cues from behavioural dominance interactions within intact colonies was not performed, the reason being a lack of ethical permission – this remains a major weakness of the study, together with only n=1 for the queen removal experiment. So, while the present data demonstrate that isopropyl myristate is a queen-associated chemosignal capable of maintaining endocrine suppression and colony stability under defined experimental manipulations, it is not possible to establish that it operates independently of other social modalities in intact colonies (or indeed other queen specific compounds). Given that the key experiment (within-colony anosmia) cannot be done, then the causal language must be moderated throughout to reflect this. Therefore, I recommend replacing “governing reproductive suppression” with language such as “capable of mediating reproductive suppression”, removing or modifying statements implying that a single pheromone “enforces eusociality” and adding an explicit limitation paragraph acknowledging that reproductive suppression in naked mole-rats is likely multimodal, with behavioural and chemosensory components operating in parallel. This is not a semantic issue, it is a matter of aligning interpretation with evidence. In terms of explaining the formation of two-queen colonies and breeder males recruitment some brief mention of this should be included in the discussion, e.g. from lines 459. In the rebuttal the authors suggest that it occurs when a reigning queen becomes reproductively impaired but this is not always so. Two queens (or even three) can co-exist and breed in asynchrony, and breeding queens can also be deposed from within by a non-breeding sister or daughter reproductively activating. There have been rare cases of dual queens also reported in the wild (see Braude’s work) so it can also occur in natural colonies too. Granted, these cases are not common, by all point to changes in dominance behaviour causing release from suppression in the presence of the queen odour and cannot be ignored.

RESPONSE: While we agree that reproductive suppression in intact colonies is likely influenced by multiple modalities, our data demonstrate that isopropyl myristate alone is sufficient to maintain endocrine suppression and colony stability across multiple independent experimental contexts (isolated pairs (n=6) and the queen-removal paradigm). The ability of a single compound to recapitulate key features of the queen’s effect, including suppression of reproduction, maintenance of high prolactin levels, and prevention of social destabilization, supports a central and causative role. It should be noted that even though our experiment with the queen removal was just N=1 we have never in 20 years of keeping naked mole-rats experienced a colony in which loss of the queen did not lead to a rapid fight for queen succession (see Barker et al. Science 2021 PMID 33510025, for examples). Therefore, it is justified to describe isopropyl myristate as a key mediator of reproductive suppression. However, we do appreciate the reviewers concerns and as requested, we have moderated our language regarding exclusivity.

In the revised manuscript, we have moderated the language throughout the Abstract, Results, and Discussion to clarify that isopropyl myristate is a queen-associated chemosignal that can

mediate reproductive suppression, rather than a single factor that alone governs eusociality. Specifcally, we changed:

- In Abstract (line in Abstract (line 4) from to "We identify a queen chemical signal that enforces this reproductive hierarchy" to "chemical signal that can enforce this reproductive hierarchy"
-
- Abstract (Line 13) "a single chemical cue governing reproductive suppression" to "a single chemical cue that can mediate reproductive suppression".
- in Introduction (line 47) "and is sufficient to prevent reproduction and maintain social stability even after queen removal." to "and can prevent reproduction and maintain social stability even after queen removal."
- in Results (line 387) "indicated that isopropyl myristate alone was sufficient to preserve reproductive suppression and prevent social destabilization in the queen's absence." to "indicated that isopropyl myristate alone was capable of mediating reproductive suppression and prevented social destabilization in the queen's absence."

2- We have revised the Discussion to explicitly acknowledge that reproductive suppression in naked mole-rats is likely multimodal, involving both behavioural and chemosensory components, and to highlight the need for future work to disentangle their relative contributions. In line 467-470 "of a chemical signal with colony-wide effects in a mammal. "Future work will be required to disentangle how this chemosensory signal, and potentially others, interact with behavioural and social cues, which likely act in a coordinated way to regulate reproductive hierarchy in natural colonies"

3- Regarding the occurrence of dual queens and breeder recruitment, we agree that such cases highlight the complexity of social dynamics in naked mole-rat colonies. We have ourselves had colonies where two queens co-exist in the laboratory. These cases are rare and their occurrence does not directly refute the existence of a chemical signal that mediates sexual suppression. Since it is clear that the Queen that produces isopropyl myristate is herself immune to the sexual suppression effects of this chemical. The problem raised by the occasional existence of dual Queens is how these females emerge as Queens without a violent dispute over the ascension. This tolerance/non-tolerance behavior may have little to do with Queen specific odours that contribute to continued reproductive suppression of non-breeders. However, because these phenomena were not directly addressed in the present study and given the word limits of the manuscript, we did not expand this section.

2. <u>Behavioural versus olfactory contributions</u>
The present study demonstrates a powerful chemosensory component but does not eliminate other mechanisms (or indeed other compounds). Although practical/ethical constraints prevented the within-colony anosmia experiments, this limitation must be more explicitly acknowledged. The current Discussion briefly notes that vomeronasal contributions cannot be excluded (although I agree that the evidence for the primary olfactory epithelium is good), but it does not adequately address the broader issue of multimodal suppression. A clear statement is needed acknowledging that behavioural dominance cues may contribute independently or synergistically.

RESPONSE: In the revised manuscript, we have added a clear statement in the Discussion acknowledging that behavioural dominance interactions and chemosensory cues may contribute together to reproductive suppression. In line 474-476 "…a chemical signal with colony-wide effects in a mammal. "Future work will be required to disentangle how this

chemosensory signal, and potentially others, interact with behavioural and social cues, which likely act in a coordinated way to regulate reproductive hierarchy in natural colonies"

3. <u>Further clarification of endocrine and reproductive measures</u>

The addition of fecal progesterone and testosterone metabolite profiling, although cruder than urinary hormone sampling is an improvement and strengthens the manuscript. However, the main text does not sufficiently clarify the lag time between circulating hormone changes and fecal metabolite detection. In the methods, n=6 pairs were used in the separation studies, yet only n=3 are included in the hormone assays (again a low n). This need to be explained. Were these part of the original study or extra experiments? If the former then the other three colonies we still don't know if lack of pregnancy in the scent transfer colonies wasn't due to deficiencies in the males. In Extended Fig. 6c, it is stated in the legend "Testosterone metabolites are significantly elevated in the blank condition compared with colony bedding and isopropyl myristate". Looking at the figure and the error bars it looks like all three groups are different from one another. Was a post-hoc test done to test for significance among all three groups? As written it implies that the blank is different from the other groups, which are not sig. diff. between each other. Is this so? If not, this needs discussion.

Lines 874-879: "Ovulation was inferred retrospectively using established reproductive staging in naked mole-rats, based on longitudinal behavioural observations, and defined reproductive states (pre-mating, mating, pregnancy, and lactation)", and on lines 882-884 "Breeding females of the four Fukomys spp. were sampled across two reproductive stages (ovulation and pregnancy), as were naked mole-rats. The three Cryptomys spp. females were likewise sampled during ovulation and pregnancy". Given that gestation in the naked mole-rat has been reported to be from 66-74 days, and the follicular phase is on average 6 days, counting back from birth to estimate ovulation could potentially entirely miss the entire follicular phase, let alone the small time window of ovulation within the follicular phase – so how confident can the authors be with their estimates of "ovulation" etc? Were any matings observed?

RESPONSE:

- We thank the reviewer for these helpful comments. We have clarified in the revised manuscript that fecal steroid metabolites reflect circulating hormone changes with a physiological delay, and we now explicitly state this limitation in the Methods. We added the following to the faecal hormone extraction section (line 1102-1104) "It should be noted that faecal steroid metabolites reflect circulating hormone changes with a physiological delay, as they represent metabolized hormone excretion rather than real-time endocrine levels.

- The pairing experiments were conducted across two locations (three replicates in Pretoria and three in Berlin), and due to technical constraints, faecal hormone analyses were performed only on samples collected in South Africa (n = 3 pairs). We have now clarified this in the Methods section (line 1085-1087) "The opposite-sex pairs experiments were conducted across two locations (as stated above). Faecal hormone analyses were performed only on samples collected in South Africa (n = 3 pairs).". Importantly, reproductive activation in the blank condition was confirmed by pregnancy outcomes and body mass gain, indicating successful mating and supporting the interpretation of the results.

- We thank the reviewer for raising this point. We have now re-examined the statistical analysis and clarified it in the revised manuscript. Differences among groups are now analysed using one-way ANOVA with a Kruskal–Wallis test and now clearly reported in the figure legend to avoid ambiguity.

- We agree that precise identification of ovulation based solely on retrospective staging is inherently limited. However, as stated in the revised manuscript, reproductive states were inferred using a combination of longitudinal behavioural observations, colony monitoring, and well-established staging criteria (pre-mating, mating, pregnancy, and lactation).
While direct confirmation of the exact timing of ovulation was not feasible, our classification captures broader reproductive states, and the key comparisons in our study rely on relative differences across these states rather than precise timing of ovulation per se. Importantly, mating events were observed in a subset of cases, providing additional support for the assigned reproductive stages.

To address this concern, we have clarified that in the Methods (line 880-882) "Ovulation was inferred retrospectively using established reproductive staging in naked mole-rats, based on longitudinal behavioural observations, and defined reproductive states (pre-mating, mating, pregnancy, and lactation). As direct determination of the ovulatory window was not feasible, this classification reflects inferred reproductive state rather than precise measurement of ovulation."

4. Copulatory plug terminology
The manuscript continues to use the term "plug formation". Even with contextual clarification, this phrasing risks anatomical inaccuracy, as naked mole-rats do not form canonical rodent copulatory plugs. I recommend replacing this terminology throughout with neutral descriptive language or removing it entirely, particularly now that endocrine data provide a more robust endpoint.

RESPONSE: We have now removed the term "plug formation" from the entire manuscript.

Minor comments/corrections
-Abstract: "These findings reveal a single chemical cue governing reproductive suppression in a mammal, linking insect and mammalian eusociality" This statement has also been indicated above as needing toning down – but also in the best characterised insect system (the honey bee) the suppressing chemosignal emitted by the queen is composed of multiple (specific) compounds, not one.

RESPONSE: As explained above, now this sentence (line 13) reads "a single chemical cue that can mediate reproductive suppression".

-Line 46: "This compound is detected by olfaction, alters prolactin and progesterone levels in non-breeders," - progesterone is only altered in non-breeder females.

RESPONSE: We have now revised the text to specify that changes occur in non-breeding females.

-Line 134: isopropyl myristate wasn't found in urine – this is interesting as urine is the most common source of rodent chemosignals. Maybe emphasise?

RESPONSE: We agree with the reviewer that this is interesting but feel that this is probably self-evident to those in the field and does not need to be explicitly discussed especially considering the strict word limits

-Lines 241-242: state whether primary or accessory olfactory bulb (I assume primary, but be clear as this is important).

RESPONSE: Since the accessory olfactory bulb (AOB) is small and embedded in the olfactory bulb and also due to concerns that we may not be able to properly distinguish the AOB from the main olfactory bulb we chose to stick with the term olfactory bulb. We established an algorithm to segment and count c-fos positive cells in the olfactory bulb and using this method we cannot be certain that cells in the AOB are also counted. This pertains to comments and suggestion made by reviewer 3 who correctly points out that a definitive separation of these two structures in the naked mole-rat would require the use of other cell markers.

-Line 458: damaransis is incorrectly spelt.

RESPONSE: We corrected it now to "*damarensis*".

Referee #3 (Remarks to the Author):

This remains an excellent paper that reports a very important finding, that isopropyl myristate is released by queens and suppresses subordinate reproduction in this eusocial mammal. I therefore, in principle, support publication in Nature.
However, I do not believe the data presented allow the investigators to claim a role for the main olfactory system, rather than the accessory olfactory systems, in mediating effects of isopropyl myristate, not withstanding the diminished size of the VNO in the NMR.

RESPONSE: We thank the reviewer for the very positive evaluation of our work and for endorsing its publication. We agree that our data do not allow us to exclude a contribution of the accessory olfactory system. Accordingly, we have removed the AOB c-Fos quantifications, the corresponding figures, and the glomeruli activation analyses to avoid a mistaken or biased interpretation of our results. We have also strengthened the fUSI section by including a positive control to validate olfactory-driven responses.

Some issues with the data presented related to cFos imaging include: (1) duplication of images in extended data 4e (compare propidium iodide with cFos) (2) uncertainty regarding identification of the AOB (extended data 4e, red circle; different in top and bottom images, and no markers used to identify it) (3) large scatter of data and n=3 on measurement of cFos in AOB (Fig 3) which preclude any conclusion regarding statistical significance (4) concern that methimazole treatment may damage the VNO, or even respiratory epithelium, absent any data indicating otherwise (5) cFos data (Fig 3 and Extended data Fig 4) remains unconvincing, absence replicates and clear cFos activity in a subset of mitral/tufted cells or glomeruli; supplementary movie is useless as it shows widespread cFos and no control.

RESPONSE: We thank the reviewer for these points. Given the concerns raised regarding data interpretation and robustness, we have removed the c-Fos imaging data for the AOB, the corresponding figures, supplementary movie, and the figure of glomeruli activation from the revised manuscript.

Regarding the new data obtained using fUSI imaging, it would be necessary to perform control experiments to confirm that areas identified indeed correspond to olfactory areas. Moreover, unilateral responses need some explanation, and replicate responses (same animal) should be shown.
Thus, for these reasons and others, I believe that the data from cFos IHC and fUSI, as currently presented, could be misinterpreted and the paper will be stronger if it is not included. Similarly, the text should be revised to keep open all possibilities (there are also olfactory subsystems that need to be considered). A deeper dive into the mechanisms by which Isopropyl myristate regulates behavior in this fascinating species certainly merits further investigation.

RESPONSE: We thank the reviewer for these constructive suggestions. To address the need for additional controls, we have now included previously acquired data using ethanol as a stimulus, which serves as a positive control. In our initial experiments, isopropyl myristate was diluted in ethanol; however, we observed that ethanol alone elicited strong activation in olfactory brain regions. For this reason, we excluded these data from the original submission and subsequently switched to DMSO as a solvent.

Ethanol is a highly volatile and ecologically relevant odorant (i.e., fermentation signal), and prior studies in mice have shown that it robustly activates olfactory circuits, including the

olfactory bulb (DOI: 10.1523/eneuro.0285-20.2020). It has also been shown that mice avoid ethanol (DOI: 10.1016/j.applanim.2019.02.003). Similarly, our observations indicate that naked mole-rats also tend to avoid alcohol. Consistent with this, we observed strong activation patterns in response to ethanol in our fUSI recordings. We have now included these data in the revised manuscript (**new Extended Data Fig. 5**) to demonstrate that our imaging approach reliably detects olfactory-driven activity. Moreover, we provide below the brain activation pattern of ethanol overlapped with the regions activated after exposure to isopropyl myristate(**Reviewer Fig. 1**).

Reviewer Fig. 1

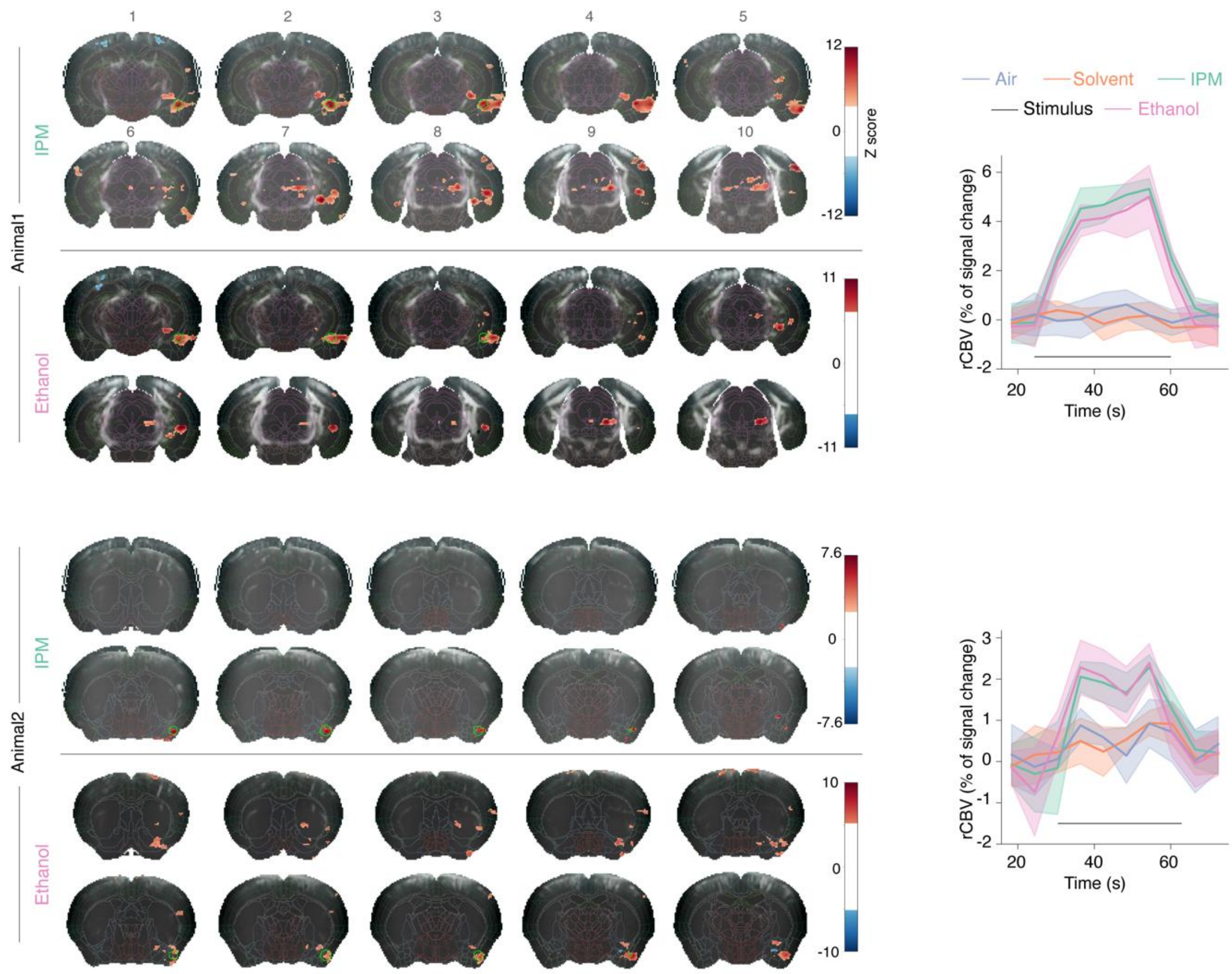

Reviewer Fig. 1. **Functional ultrasound imaging (fUSI) during olfactory stimulation with isopropyl myristate (IPM) and ethanol**. Coronal brain sections showing odour-evoked activity (Z-scores) in response to isopropyl myristate and to ethanol in two animals. Representative coronal planes (1–10, anterior to posterior) display anatomical overlays from the Allen Mouse Brain Atlas on grayscale power Doppler images, with Z-scores indicated in colour. Images represent mean projections across all replicates for each animal. Statistical thresholds ($\alpha = 0.01$, FDR-corrected with the Benjamini-Hochberg procedure) were applied. A green sphere marks the voxel of maximal response and is identically located for both IPM and ethanol conditions. The rCBV from within the green sphere is shown on the right. Traces show mean ± s.d. across imaging sessions, averaged over the stimulus period.

Regarding the unilateral responses, we have elaborated in the text to indicate that these likely reflect asymmetric nasal airflow associated with the nasal cycle, a phenomenon widely

observed in mammals: Results (line 255-256) "Notably, responses were lateralized to one hemisphere of the olfactory cortex (Fig. 3d and Extended Data Fig. 5a), which may reflect asymmetric nasal airflow associated with the nasal cycle, an autonomically regulated alternation in nasal cavity patency observed in mammals[44],[45]."

- In addition, we now explicitly state that fUSI recordings were performed across multiple sessions within the same animals "Images represent mean projections across all replicates for each animal." (line 216-217; and line 1216-1217), (Figure legends of Fig. 3d and Extended Data Fig. 5a).

- Regarding showing the fUSI replicate responses, while we agree that showing replicate responses from the same animal would be valuable, including these data would require the addition of multiple extended figures and substantially increase the length of the manuscript. Instead, we will make all replicate fUSI recordings, together with the full whole-brain c-Fos datasets, publicly available in an open repository (**Zenodo**) to ensure transparency and allow readers to access and evaluate these data in full. We also provide representative examples of these replicate responses below for clarity (**Reviewer Fig. 2**).

Reviewer Fig. 2

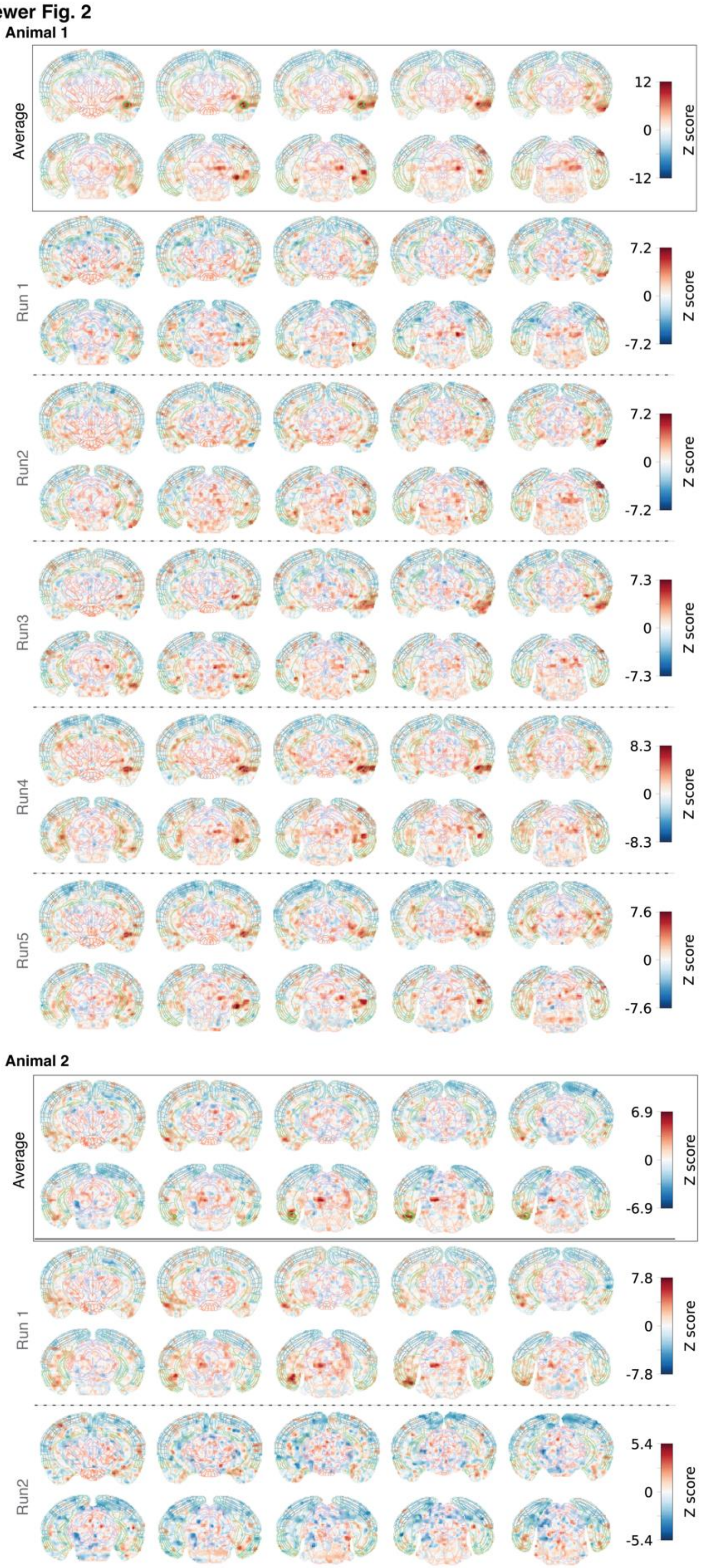

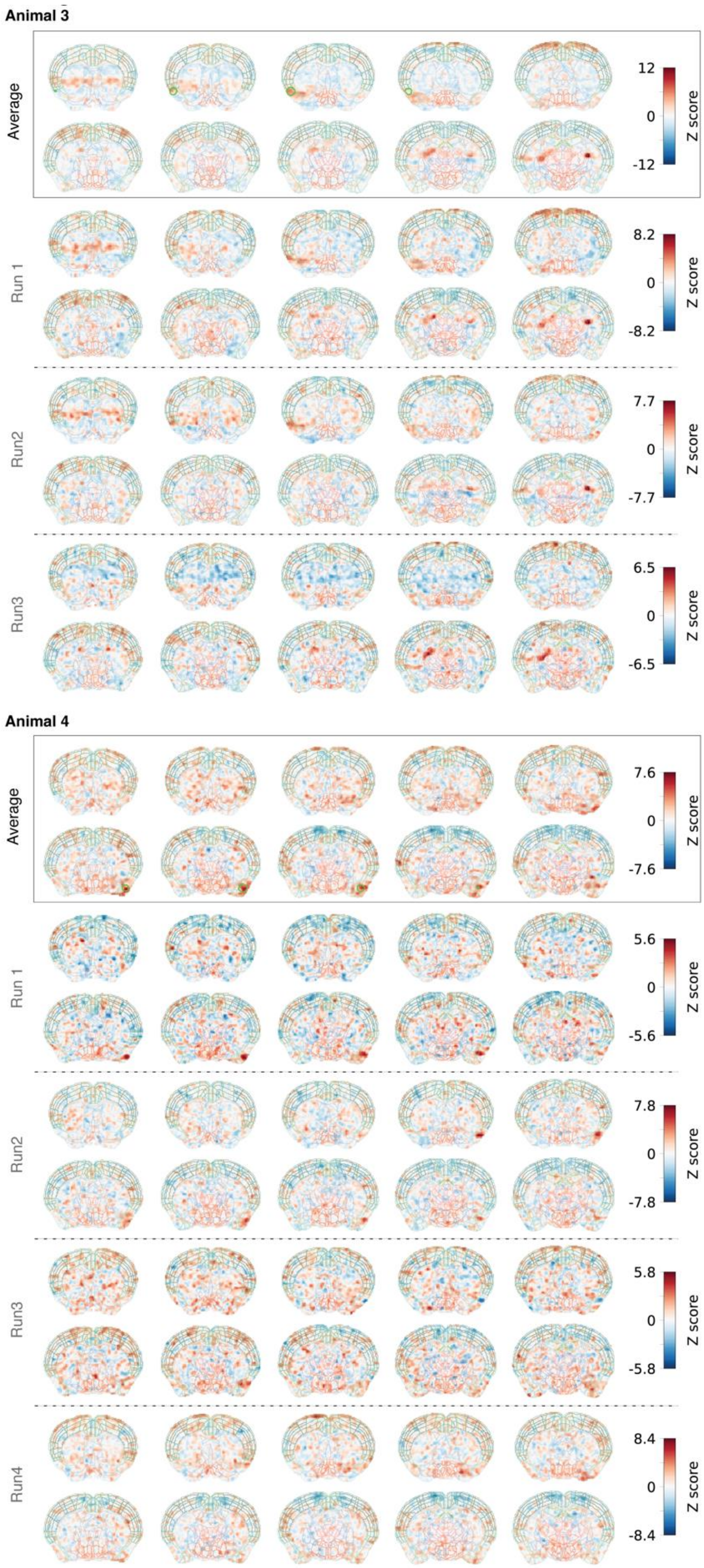

Reviewer Fig. 2 Functional ultrasound imaging (fUSI) activation maps without statistical thresholding. Coronal brain sections showing odour-evoked activity (Z-scores) in response to isopropyl myristate. Anatomical overlays from the Allen Mouse Brain Atlas are displayed on grayscale power Doppler images, with Z-scores indicated in colour. Maps are shown without statistical thresholding. For each animal, the top panels represent the mean activity across all

runs, whereas the panels below show individual runs. Representative coronal planes are arranged from anterior to posterior.

Nature 2025-09-24315B: Response to Reviewer#2

Referee #2 (Remarks to the Author):

The manuscript is substantially improved and several of my previous technical concerns have been addressed. In particular, the authors have moderated aspects of the language, clarified elements of the endocrine analyses, and removed problematic terminology. The addition of a statement acknowledging that behavioural and chemosensory cues may act in concert is also welcome (and important). However, I do not consider my main interpretive concern to be fully resolved.

We thank the reviewer for their continued and constructive comments on our work. We are pleased that the substantive improvements made in the previous revision have been recognized. Below, we address the remaining concerns and outline the changes we have made to the text.

The issue was not solely one of wording, but of ensuring that the strength of the claims is consistently aligned with the strength of the evidence. While the Abstract now uses more appropriate phrasing (e.g. "can mediate"), stronger assertions remain in several key sections of the manuscript. For example, the Introduction and Discussion still describe isopropyl myristate as a "single" or "solitary" compound capable of coordinating colony-wide reproductive suppression and sustaining eusociality.

We have removed all references to "single", "solitary", "alone", and "enforce" where they implied exclusivity or sufficiency in all sections of the MS. The offending sentences have also been changed in the introduction and discussion as follows:

<u>Introduction</u>
Line 47 Original text "These findings reveal a single, naturally occurring chemical capable of orchestrating colony-wide reproductive suppression and social cohesion, uncovering a fundamental mechanism underlying mammalian eusocial system" changed to "These findings reveal a queen-enriched chemical that contributes to reproductive suppression and social cohesion, uncovering a fundamental mechanism underlying mammalian eusocial systems."

<u>Results</u>
- The original sentence (line 382) in the results from "The prolonged nature of this hormonal and behavioural stability (12 weeks) indicated that isopropyl myristate alone was sufficient to preserve reproductive suppression and prevent social destabilization in the queen's absence" has been revised to state more explicitly what we found: "The prolonged nature of this hormonal and behavioural stability (12 weeks) indicated that under these experimental conditions, isopropyl myristate was able to mimic reproductive suppression and social stability normally associated with the presence of a queen."

- We also removed the following sentence which we felt was redundant and may be seen as making too strong a claim from the experiment described: "The emergence of aggression, pregnancy, and prolactin rebound only after isopropyl myristate withdrawal further supports a causal role in enforcing colony quiescence".

Discussion

We added the sentence (Line 469) “However, we cannot exclude the contribution of other sensory or behavioural mechanisms in maintaining reproductive suppression”

This emphasizes that we are not suggesting exclusivity here.

- We changed the sentence (Line 404) “By linking olfactory perception to prolactin-driven infertility, we show that a single compound can coordinate physiology and behaviour across the colony” to “By linking olfactory perception to prolactin-driven infertility, we show that a queen-enriched compound can in principle coordinate physiology and behaviour across the colony”.

- Final sentence (line 480) “The discovery of this molecule being involved in reproductive suppression highlights a fundamental principle of social evolution: a solitary pheromone that can enforce reproductive hierarchies, prevent conflict, and sustain eusociality in mammals.” changed to “The discovery of this molecule being involved in reproductive suppression highlights a fundamental principle of social evolution: a queen pheromone that can regulate reproductive hierarchies, prevent conflict, and sustain eusociality in mammals”

Similarly, the Results continue to frame the compound as sufficient to preserve reproductive suppression at the colony level. The issue of dual queen colonies is avoided “given the word limits of the manuscript” and should be mentioned at least briefly as an unresolved issue requiring future consideration.

We have now added a sentence to the discussion addressing this issue directly.

Line 436 “It remains unclear whether the effects of isopropyl myristate generalize to natural conditions or to rare cases of colonies with more than one breeding female5”.

Note reference 5 refers to a new reference that described multiple breeders in captive and batural colonies.

Taken together, these statements still imply a level of mechanistic sufficiency and generality that is not fully supported by the data. The study convincingly demonstrates that isopropyl myristate is a queen-associated chemosignal capable of mediating endocrine suppression and stabilizing social interactions **under defined experimental conditions**. However, very importantly, it does not establish that this compound alone governs reproductive suppression **in intact colonies independently of other queen-associated compounds or behavioural dominance interactions**. This distinction remains crucially important, particularly given that the within-colony anosmia experiment was not performed and that the queen-removal paradigm is based only on a **single colony**.

We have made several changes (see above) that we believe now align the framing of the study precisely to our findings as suggested.

Although the Discussion now includes a sentence acknowledging that behavioural and chemosensory cues may act in a coordinated manner, this point is not fully integrated into the overall framing of the study. I still think the manuscript would benefit from more consistent and explicit alignment of the central claims with this limitation throughout, particularly in the Introduction and Discussion. One example in the introduction it is stated “a single, naturally

occurring chemical capable of orchestrating colony-wide reproductive suppression and social cohesion" is still very strongly worded in terms of exclusivity when any behavioural component from the queen has not been tested. In the discussion: "a single compound can coordinate physiology and behaviour across the colony" and "a solitary pheromone that can enforce reproductive hierarchies, prevent conflict, and sustain eusociality in mammals" are problematic statements and the language asked to be removed. The title itself (which many people may not read much beyond) "A queen odour mediates reproductive suppression in a eusocial mammal" gives the impression that just a queen odour exclusively causes suppression in all naked mole-rat colonies and I would recommend rewording in some way.

I therefore consider the manuscript to be improved, but recommend further revision to ensure that the conceptual framing is fully consistent with the evidence. Without this the results are in danger of misinterpretation by the casual reader and given that NMRs are of broad and topical interest misinformation is likely to spread.

We have carefully revised the manuscript throughout to ensure that all claims are fully aligned with the experimental evidence. (see above).

Regarding the title, we appreciate this point and carefully considered revising it. However, we respectfully prefer to retain the current title: *"A queen odour mediates reproductive suppression in a eusocial mammal."* The term "mediates" does not imply exclusivity or sufficiency, but rather indicates a causal contribution.

We thank the reviewer again for the precision and engagement with our paper. We hope the revised manuscript will now be considered suitable for publication.

Referee #2 (Remarks to the Author):

The manuscript has been improved through further revision. In particular, the authors have now moderated the strongest claims throughout the manuscript, clarified that the observed effects occur under defined experimental conditions, and explicitly acknowledge that behavioural and additional sensory mechanisms may contribute to reproductive suppression in intact colonies. The inclusion of discussion regarding dual-breeder colonies is also welcomed. The only place where I still think they are stretching slightly is the final sentence: "a queen pheromone that can regulate reproductive hierarchies, prevent conflict, and sustain eusociality in mammals" This is still somewhat grand and broad, but it is much less problematic than: "a solitary pheromone that can enforce…". At this point, I personally would not hold the paper over that sentence alone. So, while some broad framing remains, I now consider the conceptual interpretation to be sufficiently aligned with the evidence presented.

We thank the referee for their considered comments on the final versions of this manuscript and we bare delighted that they now find the paper acceptable for publication. We agree with the reviewer that the final sentence of the manuscript which states ""a queen pheromone that can regulate reproductive hierarchies, prevent conflict, and sustain eusociality in mammals" **could be seen as overly broad. We have thus decided to change this sentence slightly so that it now reads as follows** "a queen pheromone that can regulate reproductive hierarchies, prevent conflict, and sustain eusociality in a mammal". **Thus, we have made the sentence more specific to the naked mole-rat and do not broaden this claim beyond this species.**
